# Interventions to reduce cognitive decline post-stroke: an umbrella review

**DOI:** 10.1093/esj/aakag122

**Published:** 2026-10-16

**Authors:** Eugene Yee Hing Tang, Emma Jackson, Ram Bajpai, Adriana Di Bernardo, Mishal Fatima, Muhammad Hamza, Jennifer Dunne, Paula Grammatikos, Claire V Burley, Rhiannon De Ivey, Khalifa Mohammed, Nele Demeyere, Linda Errington, Blossom Christa Maree Stephan, Louise Robinson, Christopher Price, Terence Quinn

**Affiliations:** Population Health Sciences Institute, Newcastle University, Newcastle upon Tyne, United Kingdom; School of Medicine, Dentistry and Nursing, University of Glasgow, Glasgow, United Kingdom; School of Medicine, Keele University, Keele, United Kingdom; Population Health Sciences Institute, Newcastle University, Newcastle upon Tyne, United Kingdom; Population Health Sciences Institute, Newcastle University, Newcastle upon Tyne, United Kingdom; Durham and Tees Valley GP Training Program, Health Education England North East and Cumbria, Newcastle, United Kingdom; Dementia Centre of Excellence, enAble Institute, Curtin University, Bentley, WA, Australia; Nuffield Department of Clinical Neurosciences, University of Oxford, Oxford, United Kingdom; Dementia Centre of Excellence, enAble Institute, Curtin University, Bentley, WA, Australia; Population Health Sciences Institute, Newcastle University, Newcastle upon Tyne, United Kingdom; Population Health Sciences Institute, Newcastle University, Newcastle upon Tyne, United Kingdom; Nuffield Department of Clinical Neurosciences, University of Oxford, Oxford, United Kingdom; Population Health Sciences Institute, Newcastle University, Newcastle upon Tyne, United Kingdom; Dementia Centre of Excellence, enAble Institute, Curtin University, Bentley, WA, Australia; Population Health Sciences Institute, Newcastle University, Newcastle upon Tyne, United Kingdom; Population Health Sciences Institute, Newcastle University, Newcastle upon Tyne, United Kingdom; Cardiovascular and Metabolic Health, University of Glasgow, Glasgow, United Kingdom

**Keywords:** cognition, intervention, meta-analysis, stroke, systematic review, umbrella review

## Abstract

**Introduction:**

Post-stroke cognitive impairment (PSCI) remains a common problem that can lead to significant disability and dependency. Previous work suggested that methodologically robust trials were lacking and that there is continued uncertainty on the benefits and limitations of pharmacological and non-pharmacological interventions. This umbrella review aims to update and synthesise evidence on pharmacological and non-pharmacological interventions for PSCI.

**Patients and methods:**

This review was pre-registered on PROSPERO (CRD42024611909) with searches from 2019 to 2024. Eligible studies were systematic reviews (SRs) of randomised controlled trials (RCTs) evaluating pharmacological or non-pharmacological interventions in adult stroke survivors (≥18 years) of any severity and reporting change in global cognition (eg, Mini-Mental State Examination). AMSTAR2 was used for risk of bias.

**Results:**

Of 4889 records identified, 69 SRs (global cognition data from 747 unique RCTs; 57,176 participants) met inclusion criteria. Interventions were grouped into 37 categories including both single (eg, acupuncture, exercise, cognitive rehabilitation) and combined modalities and assessed at categorical level. Most intervention types were associated with statistically significant improvements in global cognition. Substantial heterogeneity and wide prediction intervals indicate that effect sizes may vary considerably across populations and settings.

**Discussion:**

This review identifies promising evidence that several interventions may produce clinically meaningful improvements in global cognition post-stroke. However, confidence in this evidence is low: despite a substantial increase in the number of RCTs most included reviews were rated as critically low in quality with methodological limitations.

**Conclusion:**

Current evidence is insufficient to support firm recommendations for the inclusion of specific interventions in guidance.

## Graphical abstract

**Graphical abstract:**
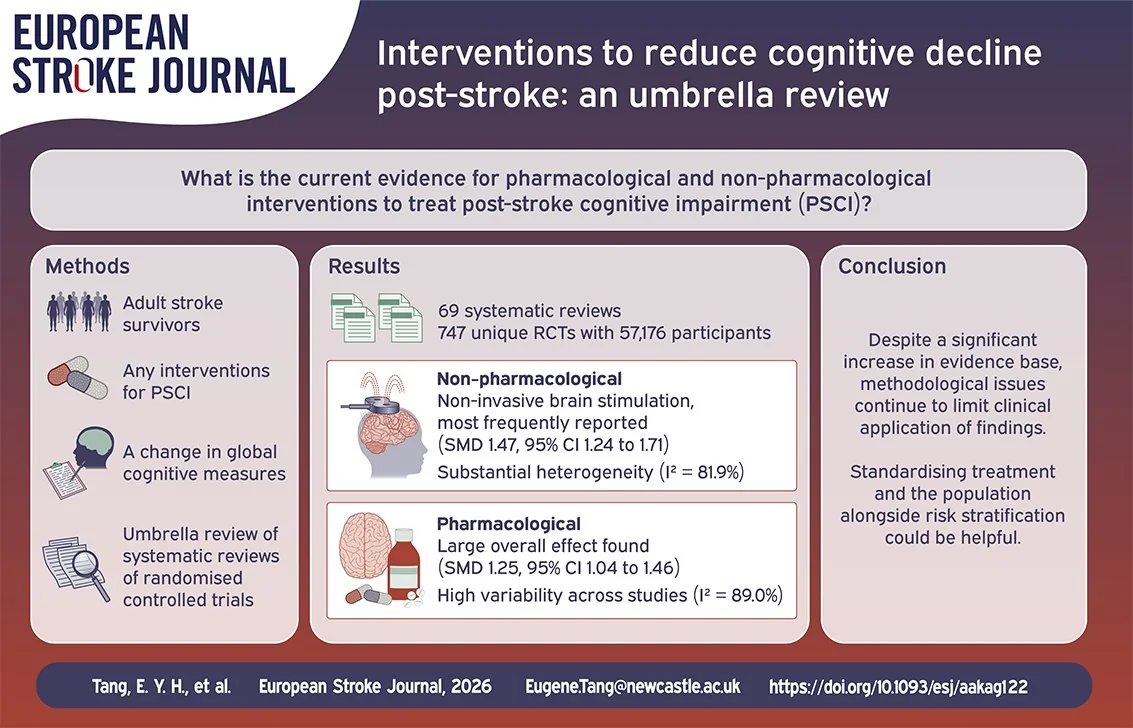

## Introduction

Although access to effective acute physical rehabilitation interventions for stroke has expanded substantially in recent years,^1^^,^^2^ healthcare systems have been slower to provide interventions that support people in the long term and people with hidden deficits. Post-stroke cognitive impairment (PSCI) is particularly common in the first-year post-stroke affecting nearly half of all stroke-survivors,^3–5^ but is often unrecognised. This is despite being associated with disability, dependency and morbidity, which adds to the challenges faced by patients, their family caregivers and care providers.^6^ It should also be recognised that cognitive recovery post-stroke is also influenced by complications during the acute phase such as delirium^7^ and infection.^8^

At present there are no recommended pharmacological treatments for PSCI in clinical guidelines. A Cochrane review did find slight beneficial effects from some anti-dementia drugs, including donepezil and galantamine, on cognition in people with vascular cognitive impairment.^9^ However, the effect size was deemed to be unlikely to be clinically important.^9^ Although perhaps not clinically relevant, given concomitant prevalence of other neurodegenerative diseases that may benefit from these drugs, international guidelines do recommend considering their use in suspected co-existing Alzheimer’s disease or Lewy Body Dementia in the context of stroke.^10^ There is some emerging evidence around the benefits of cognitive rehabilitation, particularly compensatory strategies in the context of individually relevant functional tasks, for people with PSCI.^10^ Though due to a lack of adequately powered and methodologically robust trials, there is continued uncertainty around the benefits and limitations of these interventions.^10^ The variety of potential approaches to PSCI alongside the marked paucity of high-quality data means that there is an urgent need to assess the current evidence base to prioritise which areas future research should be focussing on to address the growing burden of PSCI. An umbrella review is able to harmonise the diverse interventional approaches across settings to enable meaningful comparisons and thorough assessment.

A previous umbrella review of studies published up to 2019 identified 18 systematic reviews (SRs) (129 randomised controlled trials (RCTs); 7985 participants) evaluating treatments to improve post-stroke cognition.^11^ The authors reported that non-pharmacological treatments, including acupuncture, transcranial magnetic stimulation (TMS) and Chinese herbal medicine, improved cognitive function.^11^ Unfortunately, the included RCTs within this review were of poor methodological quality and reported imprecise estimates of treatment effects and no recommendations could be made to change practice.^11^ Since then, there has been significant interest in the development of both single and combined approaches which aim to improve post-stroke cognitive decline, necessitating a further synthesis of the available evidence in this area.

The aim of this umbrella review and meta-analysis is to update the growing evidence base around interventions for PSCI to assess whether recommendations around methodological quality have been addressed. In particular, the focus will be on global cognitive impairment. The definition of PSCI can vary in the literature. To ensure we were inclusive in our evidence synthesis, PSCI was defined as any form of global cognitive impairment that follows a stroke.

## Patients and methods

### Study design and ethics

We conducted an umbrella review, which has been reported in alignment with the Preferred Reporting Items for Systematic Reviews and Meta-Analyses guidelines.^12^ No ethical approval or informed consent for this umbrella review was required, as no primary sources of individual participant data were used.

### Search strategy and selection criteria

This original umbrella review^11^ searched the literature up to 11 June 2019. We retained the eligibility criteria, search strategy and screening procedures of the initial review, but with a focus on global cognition as the primary outcome rather than any cognitive outcome. The protocol was registered on PROSPERO (https://www.crd.york.ac.uk/PROSPERO/view/CRD42024611909).

An information specialist (LE) reviewed and adapted the search terms (see Supplementary material) and searched MEDLINE (Ovid), PsycINFO (Ovid), EMBASE (Ovid), Cochrane Library, PROSPERO and Scopus from June 2019 to November 2024. Given the multicomponent complexity of this review, there is guidance to suggest that this is less likely to be compromised by the end search data being over 12 months old^13^ particularly given that this is an umbrella review. The overlap with the initial review’s end data was retained to capture recodes added to the database after the original search. No language restrictions were applied.

Retrieved records were managed in Covidence SR software (Veritas Health Innovation, Melbourne, Australia). Title and abstract screening were conducted independently by at least 2 authors (MF, MH, JD, ADB, KM). Full text review was also conducted independently by at least 2 authors (ADB, PG, MF, MH, RDI, KM, JD, CB). Conflicts at either stage were resolved by the lead author (EYHT).

SRs of RCTs were eligible if they met the following PICOS criteria: (P) included adult (≥18 years) participants with clinically diagnosed stroke of any severity of any subtype; (I) evaluated pharmacological or non-pharmacological interventions for stroke-survivors with the aim of intervening and improving PSCI (C) compared with either no treatment or other modalities; (O) reported change in global cognition, measured using validated instruments such as the Mini-Mental State Examination (MMSE) or Montreal Cognitive Assessment (MoCA) as the outcome of the study and (S) all studies included needed to be SRs of RCTs. Reviews were excluded if they: (1) included only participants who had subarachnoid haemorrhage; (2) assessed performance in a single cognitive domain only (eg, language) or didn’t have a measure of global cognition; and (3) were not available as full peer-reviewed journal publications (eg, conference abstracts only). No restrictions were placed on study setting or follow-up duration. The primary outcome of interest was a change in global cognitive score. This was as reported by the study authors using widely used instruments such as the MMSE or MOCA. Reported changes in validated measures of global cognitive function and in biomarkers of cognitive processing (eg, P300) were also eligible for inclusion.

### Data extraction and synthesis

One author (EJ) used a pre-specified proforma to independently extract the following data from eligible included studies: study design, participants’ characteristics, intervention investigated and control group, cognitive outcome measure including direction of outcome measured post-intervention, measure of comparison post-intervention mean difference (MD), treatment duration sensitivity analysis, reporting of risk of bias, adverse events, withdrawals and Grading of Recommendations Assessment, Development and Evaluation (GRADE). All data was checked for accuracy and any uncertainty was checked and resolved through discussion with authors EYHT and RB.

### Data synthesis

To synthesise the results, interventions were categorised as pharmacological or non-pharmacological (see Table S1). Non-pharmacological interventions were divided into subgroups based on type, for example physical modality and rehabilitation therapies, as reported by each SR. Data was categorised in further subgroups where possible, but this was limited by how each SR pooled categorical data. Combination therapies were also categorised by the previous types (eg, physical modality plus pharmacological). Where feasible, we presented pooled estimates for the cognition outcomes by intervention types across SRs. We summarised effect estimates either by MD or standardised mean difference (SMD) as reported in the original meta-analysis. Random-effects meta-analysis models were used in estimating pooled effect sizes (where higher scores show better cognition and necessary conversion applied as needed). The DerSimonian and Laird method^14^ was applied to individual SR estimates to obtain a pooled summary estimate for MD or SMD in random-effects model. The 95% prediction interval (PI) was also calculated (where ≥2 studies were available), which further accounts for between-study heterogeneity and estimates the uncertainty around the effect that would be anticipated in a new study evaluating that same association. The *I*-squared statistic was used to measure the proportion of variability due to the between study heterogeneity, and its various thresholds (small, substantial and considerable) were interpreted considering the size and direction of effects, the *P*-value from Cochran’s Q test (*P* < .1 considered as significant), and the tau-squared (*τ*^2^) estimate.^15^ The overall quality of the evidence (GRADE criteria) for global cognition outcomes was extracted by one author (EJ) from each SR as reported and where available. One author (EJ) extracted SRs’ own risk of bias analyses for RCTs included in global cognition outcome from each SR. To assess overlap we calculated the corrected covered area (CCA)^16^^,^^17^ with the following definitions: moderate overlap (5% to <10%), high overlap (10% to < 15%), and very high overlap (>15%). All statistical analyses were conducted on Stata statistical software version 19.5 (StataCorp LLC, College Station, Texas, USA).

### Quality assessment

One author (EJ) used AMSTAR 2 (A MeaSurement Tool to Assess systematic Reviews) to assess risk of bias for the included SRs. This tool consists of 16 questions which are assessed as addressed (Yes or Partial Yes) or not (No), which are used to inform grading as critically low, low, moderate or high, to indicate overall confidence in the results of the SR. Evaluations specific to non-randomised studies were disregarded, as only SRs of RCTs were included in this review. Each item was evaluated and considered separately, with emphasis placed on pre-selected critical domains, as recommended by the authors of this tool.^18^ Any uncertainty was checked and resolved through discussion with EYHT.

## Results

### Summary of included studies

In total, 4889 studies were identified in the literature search as seen in Figure 1, with 358 studies assessed for eligibility after removal of duplicates and title/abstract screening. A total of 69 SRs were included in the narrative synthesis and meta-analysis. Sixteen used network meta-analysis (NMA)^19–34^ and 53 traditional meta-analysis alone.^35–87^ Global cognition data from 747 unique RCTs that included 57,176 participants was reported, 201 (26.9%) of which were included in the meta-analyses of multiple SRs. Paper overlap could not be assessed for 4 SRs that reported on 89 RCTs due to incomplete referencing.^23^^,^^29^^,^^76^^,^^81^ The number of RCTs reporting on global cognition data ranged from 2 to 87, averaging 19, across SRs (see Table 1).

**Figure 1:**
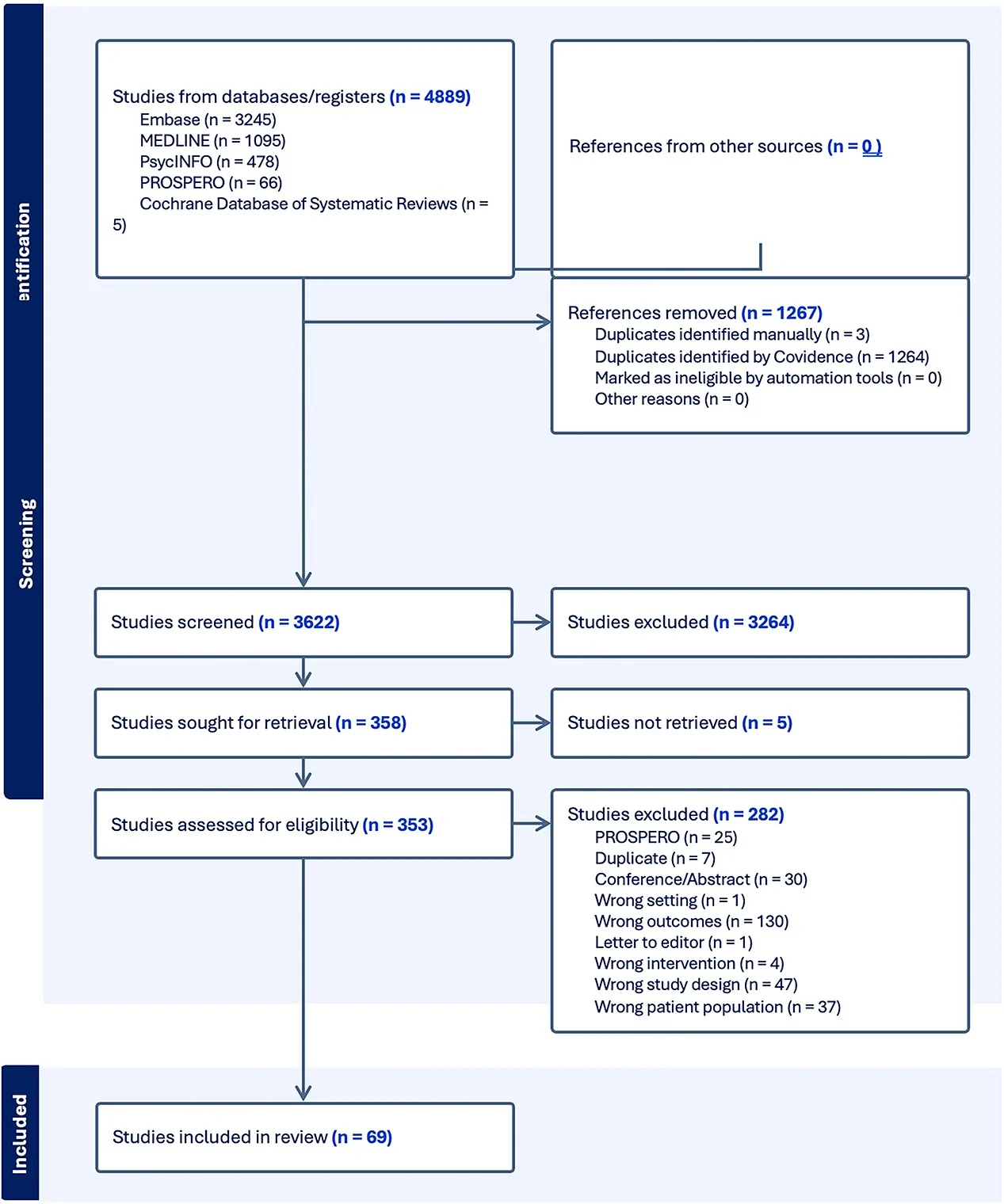
PRISMA flowchart. Preferred reporting items for systematic reviews and meta-analyses (PRISMA).

**Table 1:** Characteristics of included systematic reviews.

Systematic review paper	Location	Design	No of primary studies (RCTs) ^a^	No of participants ^a^			Age (mean range in years unless otherwise specified) ^a^	Sex distribution (% male) ^a^	Other population specifics	Intervention	Control	Time since stroke onset ^a^	Treatment duration range (mean) ^a^	Follow-up range (mean) ^a^	Intervention categories ^a^	Global cognition outcome measures
				I	C	Total										
Chen et al.^35^	China	SR	3	59	54	113	52.6–58.8	58.41%	≥6 months post-stroke	rTMS alone or in conjunction with other therapies	Sham rTMS or no rTMS	6 months–9 years	1–30 days	NR	Non-invasive brain stimulation	MMSE
Chen et al.^36^	China	SR	18	487	494	981	4 studies: 43.7–66.42 14 studies: 35–85 (non-mean range)	NR		Virtual reality (VR) rehabilitation training alongside same conventional treatments as control	Conventional treatments or conventional cognition training (without VR)	NR	2–12 weeks (5.27 weeks)^b^	NR	Virtual reality	MMSE, MoCA, LOTCA, P300 amplitude, P300 latency
Chen et al.^37^	China	SR	51	1669	1661	3330	46.35–71.7	NR		High-frequency rTMS with same conventional treatments as control	Conventional treatments alone or sham rTMS	NR	2–12 weeks (4.7 weeks)	NR	Non-invasive brain stimulation	MoCA, MMSE, P300 amplitude, P300 latency, LOTCA, ADAS-Cog, Total effective rate (difference in MMSE or ADAS-Cog)
Daoud et al.^38^	Egypt, Palestine, Syria, Sudan	SR	6	163	162	325	I: 61.4 (mean), C: 62.2 (mean)	64.45%		iTBS	Sham iTBS or conventional treatment	25 days–38 months (RCT average)	2–6 weeks (4 weeks)	NR	Non-invasive brain stimulation	MMSE, RBANS, LOTCA
Elsner et al.^39^	Germany	SR	2	36	20	56	60–69	41.07%		tDCS	Sham tDCS	Not specified for studies included in global cognition outcome.	18.5 days–3 weeks (19.5 days)	SR sought to report follow-up after 3-12 months but no evidence available—studies only reported immediately after intervention	Non-invasive brain stimulation	Computerised Neuropsychological Test, K-MMSE, SCNT
Fan et al.^40^	China	SR	26	1294	1277	2571	50–80	I: 57.1%, C: 57.68% (21 studies) Overall: 54.28% (4 studies)^b^		DL-3-n-Butylphthalide (NBP) ± same positive control drug as control	Positive control drug	NR	0.5–6 months (2.88 months)	Sought to extract but no studies described follow-up.	DL-3-n-Butylphthalide (NBP) + Oxiracetam, DL-3-n-Butylphthalide (NBP) + Nimodipine, DL-3-n-Butylphthalide (NBP) + Donepezil, DL-3-n-Butylphthalide (NBP) + Citicoline, DL-3-n-Butylphthalide (NBP)	MoCA, MMSE
Gao et al.^42^	China	SR	4	78	75	153	40.1–68.9	54.90%	>6 months post-stroke. Excluded individuals with visual impairment.	Virtual reality-based intervention (standardised computer task-oriented therapies or interactive video games) with traditional rehabilitation	Traditional rehabilitation	>6 months	4–8 weeks (5.5 weeks)	8–18 weeks (11 weeks)	Virtual reality	MoCA, MMSE
Gao et al.^42^	China	SR	6	140	145	285	44.9–66.78	60%	First stroke only	rTMS + cognitive training	Sham rTMS + cognitive training	Not described by RCT but relevant subgroup analysis	2–4 weeks (3.5 weeks)	NR	Non-invasive brain stimulation + Cognitive rehabilitation	MoCA, MMSE
Gibson et al.^43^	Australia, Taiwan	SR	11	275	267	542	43.1–65.2	56.87%^b^		Occupational therapy	Usual care	2 weeks–8 years (majority ≤18 weeks)	6 weeks–31 months (43.18 days)	3 months.^b^Only 1/11 RCTs—rest did not report f/u	Other rehabilitation therapies	MoCA, MMSE, BNIS, LOTCA
Gong et al.^44^	China	SR	10	219	218	437	42.5–69.5	59.04%	Excluded cognitive impairment due to other aetiologies.	rTMS	Sham rTMS or cognitive function training only	19.1 days–38 months (RCT average)	1-6 weeks (3.6 weeks)	NR	Non-invasive brain stimulation	MoCA, MMSE, LOTCA
Guan et al.^45^	China (in Chinese)	SR	4	159	157	316	62.37–68.7^b^	64.73%^b^	Ischaemic stroke	Ginkgo ketone ester (EGB50) + same as control group	Conventional Western medical treatment (including treatments to improve cerebral circulation, satins and neuroprotection)	<1–7 days	14–180 days (132.5 days)	NR	Ginkgo ketone ester (EGB50)	MMSE
Gunawan et al.^46^	Indonesia	SR	6	126	127	253	43.1–74.93	NR		Virtual reality intervention	Not specified	14.88 days–45.93 months^b^ (RCT average)	3–8 weeks (5.8 weeks)^b^	4–8 weeks (6.67 weeks)^b^Only 3/6 RCTs—rest did not report f/u	Virtual reality	MoCA
Han et al.^47^	China	SR	16 (only data for 2 RCTs extracted)	370	370	740	53.50–69.5	61.35%		Excitatory transcranial magnetic stimulation (HF-rTMS or iTBS) over the dorsolateral prefrontal cortex (DLPFC) of different cerebral hemispheres ± conventional rehabilitation	Sham stimulation or placebo or blank control +/- conventional rehabilitation	2.73–33.27 months (RCT average)	2–8 weeks (3.63 weeks)	2–2.5 months (2.25 months).^b^Only 2/16 RCTs—rest did not report f/u	Non-invasive brain stimulation	MMSE, MoCA, P300 latency
Han et al.^48^	China	SR	39	1022	1022	2044	51–73 (non-mean range)	51.15%	Excluded cognitive impairment due to other disease and “PSCI patients combined with other diseases like eczema, nephritis, chronic hepatitis, and connective tissue disease”	Governor vessel acupuncture therapy ± routine therapy	Routine therapy ± sham acupuncture or placebo	15 days–45 months^b^ (RCT average)	12–120 days (41.56 days)	NR	Acupuncture	MoCA, MMSE
Hofmeijer et al.^49^	Netherlands	SR	8	390			42.5–69.5	NR		Repetitive transcranial magnetic stimulation (rTMS)	Sham	5 days–38 months (RCT average)	15 days–4 weeks (23.67 days)^b^	NR	Non-invasive brain stimulation	MoCA, RBANS, MMSE
Kalbouneh 2022^50^	Jordan	SR	4	134	127	261	61.3–68.9^b^	NR		SSRI	Placebo	NR	NR	NR	SSRI	MMSE
Khattab et al.^51^	Canada	SR	6	131	151	282	50.6–79.5^b^	59.87%		Any exercise intervention	Not specified in criteria but included: track walking, usual care, relaxation, robot aided therapy, occupational therapy, stretching, waitlist, balance and flexibility exercise, no training, physical therapy, cognitive training, physiotherapy combined with occupational therapy, physical activity combined with cognitive training, and balance and flexibility exercise combined with unstructured mental activities	17 days–8.8 years (RCT average)	2.4–19 weeks (11.15 weeks)	6–10 months (8 months).^b^Only 2/6 RCTs—rest did not report f/u	Exercise	MoCA, FIM (cognitive domain), ACE-revised
Kuang et al.^52^	China	SR	28	1072	1072	2144	45.87–71.47	59.62%^b^	Excluded cognitive impairment due to other aetiologies.	Acupuncture (only therapies listed in Chinese acupuncture books and commonly used in clinic) or acupuncture-related combined therapies (excluding herbs and moxibustion)	Non-acupuncture single treatment (Western medication, basic treatment, cognitive function training; excluding traditional Chinese medical treatments)	11 days–13.33 months^b^ (RCT average)	4–12 weeks (6.79 weeks)	1–3 months (insufficient data to calculate mean)	Acupuncture	MoCA, MMSE
Li et al.^53^	China	SR	8	167	151	318	42.5–64.53	63%		rTMS	Sham rTMS	9.2–279.9 days (RCT average)	1–4 weeks (3.5 weeks)	Says extracted but NR.	Non-invasive brain stimulation	MoCA, MMSE
Li et al.^19^	China	SR and NMA	16	630	627	1257	55.56–71.55^b^	57.28%		Stimulation of special acupoints	Acupoints commonly used for PSCI	NR	2–8 weeks (3.99 weeks)	Says extracted but not described other than states few studies reported this.	Acupuncture	MoCA, MMSE
Li et al.^20^	China	SR and NMA	13	480	693 (C1: 556, C2: 137)	1173	55.13–68.3	53.03%	Excluded SAH and subdural oedema.	Physical therapy (defined as “use of physical factors such as sound, light, cold, heat, electricity and force (exercise and pressure) to restore the original state of the body by noninvasive and nondrug treatment for local or systemic dysfunction or lesions of the human body”) + acupuncture	Conventional Western medicine treatment or physical therapy alone	NR	2.1–12 weeks (4.84 weeks)	NR	Acupuncture + Non-invasive brain stimulation, Acupuncture + Electrotherapy, Acupuncture + Cognitive rehabilitation, Acupuncture + Hyperbaric oxygen therapy	MoCA, MMSE
Li et al.^21^	China	SR and NMA	29	2241			45.53–70.2	58%		Acupuncture + other complementary therapy	Acupuncture or alternative therapies alone (included HBO, rTMS, cognitive rehabilitation, TCMP and Donepezil)	NR	3–12 weeks (5.03 weeks)	NR	Acupuncture + Non-invasive brain stimulation, Acupuncture + Cognitive rehabilitation, Acupuncture + Traditional Chinese medicine, Acupuncture + Donepezil, Acupuncture + Hyperbaric oxygen therapy	MoCA, MMSE
Lin et al.^54^	China	SR	22	797	813	1610	62.86 (mean)	61.65%	Cerebrovascular disorder. Excluded individuals with other neurodegenerative or serious mental diseases that can cause cognitive impairment.	Physical activity (for ≥ 4 weeks)	No physical activity intervention or can include conventional exercise components (stretching, balance, physiotherapy) or can include if certain type of exercise was only difference between groups	<1 week–81.6 months (RCT average)	4–72 weeks (15 weeks)	Only data immediately after intervention collected. 9/22 studies reported 1-7 months follow-up (insufficient data to calculate mean).	Exercise	BNIS, FIM, ACE-R, MMSE, ADAS-Cog, MoCA, LOTCA, RPMT, SIS, multiple neuropsychological tests that evaluated across multiple domains
Liu et al.^55^	China	SR	17	603	599	1202	NR	NR		Moxibustion (and no additional therapies compared with control)	Conventional treatment and/or acupuncture or medication	NR	4–12 weeks (6.59 weeks)	NR	Moxibustion	MoCA, MMSE
Liu et al.^56^	China	SR	4	71	67	138	42.5–68.3^b^	56.52%	Excluded individuals with cognitive impairment too severe to cooperate actively with treatment or GCS < =8.	rTMS	Sham-rTMS, drug therapy or cognitive training	56–404.4 days (RCT average)	NR	NR	Non-invasive brain stimulation	MMSE, LOTCA
Liu et al.^57^	China	SR	19	1416			52.37–68.88^b^	56%^b^		Acupuncture + another therapy or standard treatment	Standard treatment or some other therapy as intervention group (excluding acupuncture)	NR	3–12 weeks (6.42 weeks)	NR	Acupuncture	MoCA, MMSE
Liu et al.^22^	China	SR and NMA	72	2814	2605	5419	42.5–72.14^b^	NR	Excluded cognitive impairment due to other aetiologies.	LF-rTMS, HF-rTMS, iTBS, cTBS	Sham stimulation or conventional therapy, or comparison among the 4 different rTMS modes	7.41 days–3.19 years (RCT average)	2-8 weeks (4.51 weeks)^b^	NR	Non-invasive brain stimulation	MoCA, MMSE, P300 amplitude, P300 latency
Liu et al.^23^	China	SR and NMA	53	2503	1876	4379	50.6–72.7^b^	56.56%^b^	Excluded individuals with aphasia, unilateral neglect, dementia or other serious mental disorders.	Nonpharmacological therapy (including combinations)	Not specified (examples given include no treatment, waiting list, standard care, routine care)	Not described by RCT but relevant subgroup analysis	10 days–18 months (11.27 weeks)^b^	NR	Exercise, Cognitive rehabilitation, Physical modality therapies, Cognitive rehabilitation/Exercise/Physical modality therapies	MMSE, FAB, MoCA, CDT, Schulte’s table test, K-MMSE, SCNT, FIM-Cog, ACE-R, CASIC-2.0, CFQ, ADAS-Cog-Plus, Simple reaction time, WCST, CogState Computerised Assessment Battery, RBANS, OCS, MBI, Stroop, LOTCA, Auditory Stroop
Liu et al.^58^	China	SR	38	1486	1485	2971	42.29–70.10^b^	55.40%	Excluded cognitive impairment due to other aetiologies.	Manual acupuncture or electro-acupuncture ± control group therapies	Cognitive rehabilitation	5.12 days–5.7 months^b^ (RCT average)	3-12 weeks (6.74 weeks)	NR	Acupuncture	MoCA, MMSE, LOTCA
Liu et al.^24^	China	SR and NMA	62	2593	2480	5073	42.29–72^b^	56.06%	Excluded cognitive impairment due to other aetiologies.	Acupuncture (single or combined use)	Other non-pharmaco-logical treatment	3.75 days–6.05 months^b^	2–12 weeks (6.07 weeks)	NR	Acupuncture, Acupuncture + Cognitive rehabilitation	MoCA, MMSE
Li et al.^59^	China	SR	5	696	695	1391	48.4–70.5	63.12%	Excluded studies that focused on individuals with Parkinson’s disease, Huntington’s disease, epilepsy, multiple sclerosis, diabetes, cognitive impairment, or mental illness.	Exercise intervention (any activity using large muscle groups maintained continuously and typically performed for extended periods on devices or ergometers including progressive resistance programmes)	Routine non-drug intervention	>6 months–2.3 years (median)^b^	<2-12 weeks (9.4 weeks)	NR	Exercise	MoCA, MMSE, ACE
Li et al.^60^	China	SR	6	273	281	554	48.4–72.0	63.90%	Excluded individuals with Parkinson’s disease, Alzheimer’s disease, dementia and other neurological disorders, or pathology/disorders that affect exercise.	Increased aerobic exercise	No increased aerobic exercise	NR	8–72 weeks (26.5 weeks)	NR	Exercise	MoCA, MMSE, ACE-R
Li et al.^61^	China	SR	7	395	391	786	53.1–72.0	81.61%	Excluded individuals with drug use records before or after exercise intervention.	Moderate and vigorous aerobic exercise training	Low intensity routine exercises	<3 months (3 RCTs), > 3 months (4 RCTs)	4–72 weeks (23.71 weeks)	NR	Exercise	MoCA, MMSE
Li et al.^25^	China	SR and NMA	55	3092			42.1–72.2^b^	55.84%^b^		Non pharmacological therapy (TMS or VR or CACR or tDCS or acupuncture)	Normal rehabilitation (traditional rehabilitation +/- cognitive training) or other therapies different from intervention group	NR	2–12 weeks (5.02 weeks)^b^	NR	Virtual reality, Cognitive rehabilitation, Non-invasive brain stimulation, Acupuncture	MoCA, MMSE
Luo et al.^62^	China	SR	27	989	984	1973	51.35–71.27	57.35%		Acupuncture	Other non-acupuncture treatments (included trials used conventional western care, nimodipine, oxiracetam, donepezil, sham acupuncture)	2 days–4.2 months (RCT average)	2–12 weeks (6.31 weeks)	NR	Acupuncture	MoCA, MMSE, P300 amplitude, P300 latency, LOTCA
Lyu et al.^63^	China	SR	18	507	486	993	51.37–68.5	NR		tDCS + control group intervention	Pharmacotherapy, RRT, CFT, computer-assisted cognitive training (CACT), +/- sham tDCS	NR	2–8 weeks (4.35 weeks)^b^	NR	Non-invasive brain stimulation	MoCA, MMSE, P300 latency
Ma et al.^64^	China	SR	14	362	360	722	52.6–74.3	59.14%		VR technology	Other methods	10 days–41.1 months (RCT average)	4–24 weeks (6.35 weeks)^b^	NR (other than “varied”)	Virtual reality	MoCA, MMSE
Mingming et al.^65^	China	SR	6	201	184	385	Not extractable for relevant studies only	NR	Excluded cognitive impairment due to other aetiologies.	Computer-based cognitive training	Routine cognitive rehabilitation	33% acute, 50% chronic^b^	2–12 weeks (5.67 weeks)	NR	Cognitive rehabilitation	MMSE, MoCA, CFQ, LOTCA
Nie et al.^66^	China	SR	18	489	492	981	NR	NR		Computer-assisted cognitive rehabilitation + same conventional treatment as control group	Conventional therapy alone	NR	4–12 weeks (7.18 weeks)^b^	States no studies reported follow-up measurements.	Cognitive rehabilitation	MoCA, MMSE, LOTCA
Rose Sin Yi et al.^67^	USA, Hong Kong, Czech Republic	SR	12	285	281	566	43.7–72.95 (mean range)	50.95%		Virtual reality-based cognitive interventions	Any (including OT/PT motor training, combination of conventional computerised and pencil-and-paper cognitive training, individual-format intervention, recreational activities [video games], computer-assisted cognitive rehabilitation, usual care, waitlist)	NR	4–10 weeks (6.5 weeks)	Mentions “only a few studies” followed participants. Not described in further detail.	Virtual reality	MoCA, MMSE, FIM, ACE, Timed up and go cognition test
Shen et al.^68^	China	SR	34	1356	1355	2711	56.5–72.6^b^	56.92%^b^		Traditional Chinese medicine (prescriptions containing multiple herbs, a single herb, or Chinese patent medicines) + conventional western medicine (featuring expert consensus recommended drugs)	Same conventional western medication	12.48 hours–3.12 years^b^ (RCT average)	0.5–6 months (2.51 months)	“Most studies lacked follow-up”. No further details.	Traditional Chinese medicine	MoCA, MMSE
Toh et al.^69^	Singapore, Norway, Finland	SR	8	214	240	454	24–78.1	NR	Post-stroke sleep-disordered breathing	Non-invasive positive airway pressure therapy	No treatment, conventional treatment or placebo	NR	3 weeks–12 months (11.38 weeks)	6-9 months (7.5 months)^b^Only able to clearly differentiate reported f/u from treatment period for 2/8 RCTs. Others do not appear to differ from treatment durations.	Non-invasive ventilation	MMSE, MoCA, cognitive subscale of Canadian Neurological Scale
Valenzuela-Lopez et al.^26^	Spain	SR and NMA	11	281	288	569	50.71–77.3	58.00%	Hand function deficits post-stroke. Excluded individuals with any other neurologic diseases or dementia.	Hand motor interventions (included studies assessed VR, robot-assisted, hand-grip strength)	Conventional hand rehabilitation and not including any formal cognitive intervention	6.51–389.03 days^b^ (RCT average)	10 days–3 months (6.31 weeks)	NR	Virtual reality, Other rehabilitation therapies, Exercise	MoCA, MMSE
Wang et al.^70^	Taiwan	SR	3	52	50	102	52.6–68.12	NR		Game-based VR upper limb rehabilitation using computers, videogames, mobile apps and VR glasses	Conventional therapy including PT and OT	14.88–180 days (RCT average)	3–6 weeks (4.33 weeks)	NR	Virtual reality	MoCA
Wang et al.^27^	China	SR and NMA	12	460			42.5–68.5	59.88%		NIBS	Sham NIBS ± conventional/computerised cognitive treatment	19.13 days–3.97 years (RCT average)	2–4 weeks (2.92 weeks)	NR	Non-invasive brain stimulation	MoCA, MMSE
Wang et al.^28^	China	SR and NMA	16	386	365	751	42.5–69.5	56.98%		NIBS	Sham NIBS	19.13 days–8.79 months^b^ (RCT average)	2–4 weeks (3.63 weeks)	“Most did not have a long-term follow-up after treatment”	Non-invasive brain stimulation	MoCA, MMSE
Wang et al.^71^	China	SR	38	5417			35–73	56.15%		NBP ± control medications	Drugs such as donepezil, galantamine, memantine, olaxetan and nimodipine as per expert consensus or only medications for the patients’ pre-existing diabetes	NR	0.5–6 months (2.06 months)^b^	NR	DL-3-n-butylphthalide (NBP) + Urinary kallidinogenase, DL-3-n-butylphthalide (NBP) + Piracetam, DL-3-n-butylphthalide (NBP) + Oxiracetam, DL-3-n-butylphthalide (NBP) + Nimodipine, DL-3-n-butylphthalide (NBP) + Idebenone, DL-3-n-butylphthalide (NBP) + Huperzine A, DL-3-n-butylphthalide (NBP) + Galanthamine, DL-3-n-butylphthalide (NBP) + Edavarone, DL-3-n-butylphthalide (NBP) + Donepezil, DL-3-n-Butylphthalide (NBP) + Citicoline, DL-3-n-butylphthalide (NBP)	MoCA, MMSE
Wang et al.^72^	China	SR	14	507	507	1014	46.38–68	NR		Acupuncture + routine therapy	Routine treatment	NR	4–16 weeks (7.49 weeks)	NR	Acupuncture	MoCA, MMSE, LOTCA
Wiley et al.^73^	Canada	SR	5	63	59	122	52.6–68.9^b^	52.46%		Virtual reality-based intervention	Any control intervention accepted (included studies used cognitive rehabilitation through traditional methods (math games, puzzles and conflict resolution and memory tasks), computerised cognitive training, conventional occupational therapy or physiotherapy and wait list)	23 days–41 months (RCT average)	4–6 weeks (4.8 weeks)	1 month (1 month).^b^Only 3/5 RCTs—rest did not report f/u	Virtual reality	MoCA, K-MoCA, MMSE, K-MMSE
Xiao et al.^74^	China	SR	5	165	165	330	56.13–66.63	41.33%		Acupuncture + rTMS	Either rTMS alone or in combination with conventional Western medicine	2.02–4.15 months (RCT average)	4–12 weeks (6.4 weeks)	NR	Acupuncture + Non-invasive brain stimulation	MoCA, MMSE
Xiao et al.^29^	China	SR and NMA	21	543	504	1047	52.6–74.3	NR		Virtual reality or CACR	Traditional treatment	NR	4–24 weeks (6.62 weeks)^b^	NR	Virtual reality, Cognitive rehabilitation	MoCA, MMSE
Xie et al.^75^	China	SR	4	77	77	154	42.5–63.87	62.99%	Post-stroke memory disorder > 2 weeks	rTMS	Sham rTMS or non-rTMS	1.07 months–105.83 days (RCT average)	4 weeks (4 weeks)	NR	Non-invasive brain stimulation	MoCA
Xu et al.^30^	China, Austria	SR and NMA	87	3062	2933	5995	42.5–72.6^b^	58.86%	Post-stroke cognitive impairment and dementia (PSCID)	Scalp stimulation	Placebo or best available therapy	11.42–1244.65 days^b^ (RCT average)	14–90 days (31.66 days)^b^	NR	Non-invasive brain stimulation, Acupuncture	MoCA, MMSE, HDS, LOTCA, P300 amplitude, P300 latency
Xu et al.^76^	China	SR	8	247	247	494	42.5–60.15	60.93%	First stroke only, stable vital signs. Excluded individuals with severe aphasia, disability or cognitive disorder	rTMS	Conventional treatment + sham simulation	1.43 months–132 days (RCT average)	4–8 weeks (4.75 weeks)	NR	Non-invasive brain stimulation	MoCA
Yang et al.^31^	China	SR and NMA	22	1068	1052	2120	52.3–84.2	58.40%		Non-pharmacological interventions (Reminiscence therapy (RT), remote ischemic conditioning (RIC), physical fitness training (PFT), intensive patient care program (IPCP), grip training (GT), cognitive behavioural therapies (CBT), cognitive rehabilitation training (CRT), high-pressure oxygen therapy (HPO), repetitive trans-cranial magnetic stimulation (rTMS), trans-cranial direct current stimulation (tDCS), moxibustion, moderate intensity continuous training (MICT), and moderate-intensity continuous training + high-intensity interval training (MICT + HIIT)) + same rehabilitation training as control group	Standard clinical treatments but no cognition-improving medications	NR	NR	NR	Hyperbaric oxygen therapy, Exercise, Cognitive rehabilitation, Non-invasive brain stimulation, Moxibustion, Acupuncture, Remote ischemic conditioning, Intensive patient care program	MoCA, MMSE
Yang et al.^77^	China	SR	6	147	140	287	52.69–78.1	65.85%	TIA or stroke with OSA	CPAP	Sham or usual care	7 days–6 months	3–24 weeks (7.83 weeks)	2–9 months (5.67 months)^b^Only 3/6 RCTs—rest did not report f/u	Non-invasive ventilation	MMSE, ACE, FIM [cognitive component]
Yang et al.^32^	China	SR and NMA	25	910	794	1704	42.5–72.14	NR	Clear consciousness and stable vital signs.	TMS	Conventional rehabilitation or sham TMS	19.13 days–28.35 months (RCT average)	2–8 weeks (3.83 weeks)^b^	NR	Non-invasive brain stimulation	MoCA, MMSE, P300 latency, P300 amplitude
Yan et al.^33^	China	SR and NMA	12	328	260	588	42.5–68.5	66.67%		Non-invasive brain stimulation	Placebo or sham	6.07 days–28.35 months (RCT average)	NR	NR	Non-invasive brain stimulation	MoCA, MMSE
Yan et al.^78^	China	SR	10	292	290	582	53.14–68.5	62.21%^b^		tDCS	Sham tDCS or no additional intervention	15.1–68.5 days (RCT average)	NR	3–12 weeks (6.33 weeks)^b^Only reported for 9/10 RCTs, but no treatment duration mentioned so not clear if follow-up is beyond treatment duration.	Non-invasive brain stimulation	MoCA, MMSE
Yikun et al.^79^	China	SR	15	424	391	815	42.5–71.7^b^	57.29%^b^		rTMS (high-frequency > 1 Hz or low-frequency ≤ 1 Hz) + conventional rehab, cognitive training, medication or acupuncture	Conventional rehabilitation, cognitive training, medication, acupuncture or sham	NR	10 days–8 weeks (3.88 weeks)^b^	NR	Non-invasive brain stimulation	MoCA, MMSE, P300 amplitude, P300 latency
Zhang et al.^81^	China, USA	SR	7	105	105	210	Not extractable for relevant studies only	NR		Virtual reality rehabilitation therapy	Conventional rehabilitation or placebo	NR	4–12 weeks (5.71 weeks)	NR	Virtual reality	MMSE
Zhang et al.^80^	China, UK	SR	10	162	161	323	52.6–74.33	NR		Virtual reality-based therapies (include either single-component interventions or multiple-component interventions for patients with stroke. VR had to consist of a screen or a head-mounted device, including games with immersive, semi-immersive, and non-immersive systems, simulating virtual environments using computers, video consoles, mobile apps, and VR)	Usual care or non-VR interventions	NR	4–8 weeks (5 weeks)	NR	Virtual reality	MoCA, MMSE, LOTCA
Zhang et al.^82^	China	SR	11	679	703	1382	NR	NR	Cognitive decline within 6 months of stroke	Exercise therapy	Routine non-pharmacological intervention, including a balanced diet, health education, and routine rehabilitation training	NR	4–72 weeks (18 weeks)^b^	NR	Exercise	MoCA, MMSE
Zhao et al.^83^	China	SR	23	505	462	967	42.5–69.5	55.77%^b^	Excluded individuals with language disorders.	Non-invasive brain stimulation	Sham rTMS or tDCS or cognitive function training alone	10.37-999 days^b^ (RCT average)	1–8 weeks (3.55 weeks)^b^	NR	Non-invasive brain stimulation	MMSE, MoCA, LOTCA, may include other measures (not clearly described)
Zhou et al.^34^	China	SR and NMA	46	1424	1359	2783	42.1–74.93	63.35%		Cognitive training (including traditional cognitive training, CBCT, VRCT, and multi-component cognitive training (including EX-CT), and multi-component cognitive training (including exercise combined with cognitive training))—individually or in combination	Exercise, traditional rehabilitation therapy, or alternative forms of cognitive training	NR	10 days–18 weeks (6.54 weeks)^b^	NR.	Virtual reality + Cognitive rehabilitation, Virtual reality, Exercise + Cognitive rehabilitation, Exercise, Cognitive rehabilitation	MoCA, MMSE, LOTCA
Zhou et al.^84^	China, USA	SR	37	1442	1427	2869	35–80 (non-mean range)	Incomplete		Acupuncture	Sham or no treatment	3–1080 days	2–12 weeks (6.30 weeks)	NR	Acupuncture	MoCA, MMSE
Zhou et al.^85^	China	SR	10	211	198	409	43.1–65	NR	Excluded individuals with poor motor function, visual field defect or aphasia, unable to understand instructions, with dementia, with any type of brain injury other than stroke.	Computerised cognitive training	TCR (PT and OT), usual care or any other treatments without intervention tasks (mock or sham training)	70% acute, 30% chronic	10 days–12 weeks (6.34 weeks)	1–12 months (4.67 months)^b^Only 3/10 RCTs—rest did not report f/u	Cognitive rehabilitation	MoCA, MMSE, K-MMSE, LOTCA
Zhu et al.^86^	China	SR	10	414			42.5–69.4	NR	Excluded individuals with severe cognitive decline that impedes cooperation, cognitive impairment or dementia before stroke.	rTMS or iTBS	Sham or no stimulation	0.76-38 months (RCT average)	5–30 days (17.2 days)	NR	Non-invasive brain stimulation	MoCA, MMSE, LOTCA, RBANS
Zhuo et al.^87^	China	SR	15	705	697	1402	42.29–70	NR	Excluded cognitive impairment due to other aetiologies.	Acupuncture + rehabilitation training	Not specified	NR	4–12 weeks (7.22 weeks)	NR	Acupuncture + Rehabilitation therapies	MoCA, MMSE

Abbreviations: NR = not reported; RCT = randomised controlled trial; SR = systematic review.

a Figures for global cognition outcome, as reported in SR.

b From available data. Not all RCTs included in SR reported this information or reported in non-comparable way.

Most SRs (*n* = 57) included meta-analyses for individual intervention categories alone. The remainder (*n* = 12) reported interventions from multiple categories. Physical modality interventions were reported by 38 SRs. This group included non-invasive brain stimulation (*n* = 24),^22^^,^^25^^,^^27^^,^^28^^,^^30–33^^,^^35^^,^^37–39^^,^^44^^,^^47^^,^^49^^,^^53^^,^^56^^,^^63^^,^^75^^,^^76^^,^^78^^,^^79^^,^^83^^,^^86^ acupuncture alone (n-12),^19^^,^^24^^,^^25^^,^^30^^,^^31^^,^^48^^,^^52^^,^^57^^,^^58^^,^^62^^,^^72^^,^^84^ acupuncture combined with non-invasive brain stimulation (*n* = 3),^20^^,^^21^^,^^78^ and moxibustion (*n* = 2).^31^^,^^55^ Rehabilitation therapies were reported by 25 SRs. This included virtual reality (*n* = 13),^25^^,^^26^^,^^29^^,^^34^^,^^36^^,^^41^^,^^46^^,^^64^^,^^67^^,^^70^^,^^73^^,^^80^^,^^81^ exercise (*n* = 10),^23^^,^^26^^,^^31^^,^^34^^,^^51^^,^^54^^,^^59–61^^,^^82^ cognitive rehabilitation (*n* = 8),^23^^,^^25^^,^^29^^,^^31^^,^^34^^,^^65^^,^^66^^,^^85^ and other rehabilitation therapies (n-2).^26^^,^^43^ Pharmacological interventions were reported by 5 SRs including 2 that reported on DL-3-n-butylphthalide (NBP) alone or combined with other medication.^40^^,^^71^ Combined (physical modality combined with rehabilitation) interventions were reported by 5 SRs including 3 that reported on acupuncture combined with cognitive rehabilitation.^20^^,^^21^^,^^58^ Other interventions were reported in 3 SRs including 2 reporting on non-invasive ventilation.^69^^,^^77^ Combined (physical modality combined with other) was reported in 2 SRs that described acupuncture combined with hyperbaric oxygen therapy.^20^^,^^21^ Other categories were only reported by single SRs (see Table 1).

### Participant characteristics

The total number of participants for global cognition outcome ranged from 56 to 5995 and averaged 1309 across included SRs. Sixty-four out of 69 SRs reported mean age or age range of participants for included RCTs. Sex distribution ranged from 41.07% to 81.61% male but was not reported by 22 SRs.^22^^,^^29^^,^^32^^,^^36^^,^^37^^,^^46^^,^^49^^,^^50^^,^^55^^,^^63^^,^^65^^,^^66^^,^^69^^,^^70^^,^^72^^,^^80–82^^,^^84–87^ Forty-five out of 47 SRs that reported sex distribution included more male than female participants. Most SRs did not specify stroke type for inclusion. One study only included participants with ischaemic stroke.^45^ Two SRs only included participants following their first stroke.^42^^,^^76^ In addition to stroke, one only included participants who also had hand function deficits post-stroke,^26^ one only included participants with post-stroke sleep-disordered breathing,^69^ and one only included participants who also had obstructive sleep apnoea.^77^

### Global cognition outcome measures

MoCA (*n* = 61) and MMSE (*n* = 60) were the most used global assessment tools (see Table 1). Others SRs used global cognitive assessments including LOTCA (*n* = 20), P300 latency (*n* = 9), P300 amplitude (*n* = 7), Addenbrooke’s Cognitive Examination (ACE and ACE-R) (*n* = 7), Functional Independence Measure (FIM) cognitive domain (*n* = 5), Repeatable Battery for the Assessment of Neuropsychological Status (RBANS) (*n* = 4), Korean MMSE (K-MMSE) (*n* = 4), Alzheimer Disease Assessment Scale-Cognitive Subscale (ADAS-Cog and ADAS-Cog-Plus) (*n* = 3), Seoul Computerised Neuropsychological Test (SCNT) (*n* = 2), Barrow Neurological Institute Screen (BNIS) (*n* = 2), and Cognitive Failures Questionnaire (CFQ) (*n* = 2). Twenty other tools were described in single reviews, and one review also combined multiple neuropsychological tests that evaluated multiple cognitive domains.

### Treatment duration and follow-up

Treatment duration was described for the majority of included RCTs and ranged from 1 day to 31 months, averaging 6.99 weeks across all RCTs. Five SRs did not describe treatment duration.^31^^,^^33^^,^^50^^,^^56^^,^^78^ Follow-up was described for a limited number of RCTs in 12 SRs.^41^^,^^43^^,^^46^^,^^47^^,^^51^^,^^52^^,^^54^^,^^69^^,^^73^^,^^77^^,^^78^^,^^85^ Treatment duration and follow-up are summarised in Table 1 and by intervention type in Table S2.

### Interventions and meta-analysis results by category

#### Standardised mean difference

When cognitive outcomes were synthesised using SMD, different intervention types demonstrated statistically significant improvements compared with controls, although the magnitude and consistency of effects varied across categories. Overall, most intervention types were associated with statistically significant improvements in cognitive outcomes, although heterogeneity and wide prediction intervals suggest that effect sizes may vary considerably across populations and settings (see Table 2).

**Table 2:** Quantitative synthesis for cognitive outcome by intervention type when reported as standardised mean difference.

Interventions categories by their subtypes	No of SRs	Studies in MA	Participants in MA	SMD (95% CI)	95% PI	*I* ^2^ (%)	*τ* ^2^
**Combined (Physical modality + Rehabilitation)**							
**Non-invasive brain stimulation + Cognitive rehabilitation**	1	6	285	0.78 (0.48–1.08)	–	–	–
**Physical modality**							
**Acupuncture**	2	26	2,002	0.89 (0.78–1.00)	(0.76, 1.02)	0.0	0.000
**Non-invasive brain stimulation**	12	144	9,950	1.47 (1.24–1.71)	(−0.02, 2.96)	81.9	0.541
**Physical modality therapies**	1	17	1,430	0.60 (0.26–0.95)	–	–	–
**Combined**	15	186	13,301	1.34 (1.16–1.52)	(0.15, 2.52)	80.2	0.347
**Rehabilitation**							
**Cognitive rehabilitation**	3	33	2,172	0.63 (0.42–0.85)	(−0.74, 2.01)	0.0	0.000
**Exercise**	7	57	5,915	0.35 (0.17–0.54)	(−0.23, 0.93)	84.0	0.042
**Virtual reality**	5	16	571	0.41 (0.13–0.69)	(−0.05, 0.86)	0.0	0.000
**Other rehabilitation therapies**	1	11	600	0.58 (0.40–0.76)	–	–	–
**Combined**	15	106	8,603	0.44 (0.29–0.59)	(−0.07, 0.95)	78.7	0.052
**Pharmacological**							
**DL-3-n-Butylphthalide (NBP)**	2	21	2,192	0.86 (0.65–1.07)	(−1.52, 3.25)	71.6	0.024
**DL-3-n-Butylphthalide (NBP) + Citicoline**	2	6	520	1.51 (1.24–1.77)	(−0.21, 3.23)	0.0	0.000
**DL-3-n-Butylphthalide (NBP) + Donepezil**	2	2	278	0.97 (0.75–1.18)	–	0.0	0.000
**DL-3-n-Butylphthalide (NBP) + Edavarone**	1	1	100	5.54 (4.67–6.42)	–	–	–
**DL-3-n-Butylphthalide (NBP) + Galanthamine**	1	1	84	1.49 (1.01–1.98)	–	–	–
**DL-3-n-Butylphthalide (NBP) + Huperzine A**	1	1	100	2.03 (1.55–2.52)	–	–	–
**DL-3-n-Butylphthalide (NBP) + Idebenone**	1	1	60	0.44 (−0.07 to 0.95)	–	–	–
**DL-3-n-Butylphthalide (NBP) + Nimodipine**	2	4	362	1.27 (0.59–1.95)	(−6.97, 9.50)	84.4	
**DL-3-n-Butylphthalide (NBP) + Oxiracetam**	2	14	1,568	1.13 (0.73–1.53)	(−0.55, 2.80)	76.4	
**DL-3-n-Butylphthalide (NBP) + Piracetam**	1	3	225	1.00 (0.71–1.30)	–	–	–
**DL-3-n-Butylphthalide (NBP) + Urinary kallidinogenase**	1	3	222	0.77 (0.58–0.97)	–	–	–
**Combined**	2	53	5,382	1.25 (1.04–1.46)	(0.27, 2.22)	89.0	
**Non-pharmacological**							
**Cognitive rehabilitation/Exercise/Physical modality therapies**	1	7	686	1.37 (0.83–1.92)	–	–	–
**Other**							
**Non-invasive ventilation**	2	9	490	0.40 (−0.22 to 1.02)	(−1.52, 1.93)	58.9	0.132

Abbreviations: PI = prediction interval; SMD = standardised mean difference.

### Combined interventions

The combination of physical modalities and rehabilitation showed a large overall effect (SMD 0.78, 95% CI, 0.48–1.08) in a single review evaluating non-invasive brain stimulation plus cognitive rehabilitation.

### Physical modality interventions

Among physical modalities, non-invasive brain stimulation yielded a large, pooled effect (SMD 1.47, 95% CI, 1.24–1.71), but heterogeneity was substantial (*I*^2^ = 81.9%) and the prediction interval crossed the null (−0.02 to 2.96). Acupuncture showed a large and precise effect (SMD 0.89, 95% CI, 0.78–1.00) with no observed heterogeneity (*I*^2^ = 0%). Other physical modality therapies demonstrated a moderate effect (SMD 0.60, 95% CI, 0.26–0.95). The overall pooled estimate across physical modalities remained large (SMD 1.34, 95% CI, 1.16–1.52), though heterogeneity was high (*I*^2^ = 80.2%; τ^2^ = 0.347).

### Rehabilitation interventions

Cognitive rehabilitation was associated with a moderate effect (SMD 0.63, 95% CI, 0.42–0.85) with no statistical heterogeneity, although the prediction interval was wide (−0.74 to 2.01). Exercise showed a small but significant effect (SMD 0.35, 95% CI, 0.17–0.54) with high heterogeneity (*I*^2^ = 84.0%). Virtual reality interventions demonstrated a small to moderate effect (SMD 0.41, 95% CI, 0.13–0.69) with no observed heterogeneity. When pooled, rehabilitation interventions showed a moderate overall effect (SMD 0.44, 95% CI, 0.29–0.59), with substantial heterogeneity (*I*^2^ = 78.7%).

### Pharmacological interventions

Pharmacological therapies generally demonstrated moderate to large effects. NBP alone showed a large effect (SMD 0.86, 95% CI, 0.65–1.07) with substantial heterogeneity (*I*^2^ = 71.6%). Combinations with other agents often yielded larger effects, including NBP plus citicoline (SMD 1.51, 95% CI, 1.24–1.77), NBP plus huperzine A (SMD 2.03, 95% CI, 1.55–2.52), and NBP plus galanthamine (SMD 1.49, 95% CI, 1.01–1.98). One combination (NBP plus edavarone) reported an exceptionally large effect (SMD 5.54, 95% CI, 4.67–6.42), although this was based on a single review. The pooled estimate across pharmacological combinations was large (SMD 1.25, 95% CI, 1.04–1.46), with very high heterogeneity (*I*^2^ = 89.0%).

### Other interventions

Non-invasive ventilation showed a non-significant effect (SMD 0.40, 95% CI, −0.22 to 1.02) with moderate heterogeneity (*I*^2^ = 58.9%). A single nonpharmacological synthesis combining cognitive rehabilitation, exercise and physical modalities reported a large effect (SMD 1.37, 95% CI, 0.83–1.92).

#### Mean difference

When outcomes were synthesised as MDs on specific cognitive scales, most intervention types demonstrated statistically significant improvements, particularly when measured using the MMSE and MoCA scales. In summary, MD analyses confirmed that both physical and rehabilitation-based interventions, as well as several pharmacological therapies, were associated with improvements in cognitive scale scores, although heterogeneity varied widely across intervention types and outcome measures (see Table 3).

**Table 3:** Quantitative synthesis for cognitive outcome by intervention type when reported as mean difference.

Interventions categories by their subtypes	No of SRs	Studies in MA	Participants in MA	Scale used	MD (95% CI)	95% PI	*I* ^2^ (%)	*τ* ^2^
**Combined (Physical modality + Pharmacological)**								
**Acupuncture + Donepezil**	1	6	475	MMSE	1.95 (1.06–2.84)	–	–	–
**Acupuncture + Traditional Chinese medicine**	1	2	244	MMSE	4.27 (3.19–5.34)	–	–	–
**Acupuncture + Traditional Chinese medicine**	1	4	346	MoCA	3.99 (3.09–4.88)	–	–	–
**Combined (Physical modality + Rehabilitation)**								
**Acupuncture + Cognitive rehabilitation**	3	31	2,715	MMSE	3.00 (2.19–3.82)	(0.12, 5.89)	80.8	1.608
**Acupuncture + Cognitive rehabilitation**	2	25	1,950	MoCA	2.86 (2.00–3.72)	(0.20, 5.51)	77.1	0.984
**Acupuncture + Rehabilitation therapies**	1	12	1,224	MMSE	2.85 (2.56, 3.15)	–	–	–
**Acupuncture + Rehabilitation therapies**	1	7	659	MoCA	2.18 (1.38–2.97)	–	–	–
**Combined (Physical modality + Other)**								
**Acupuncture + Hyperbaric oxygen therapy**	2	7	588	MMSE	3.73 (3.03–4.43)	(2.86, 4.60)	0.0	0.000
**Acupuncture + Hyperbaric oxygen therapy**	2	6	607	MoCA	3.26 (2.54–3.98)	(2.07, 4.45)	8.0	0.079
**Physical modality**								
**Acupuncture**	10	149	12,121	MMSE	2.48 (1.93–3.03)	(0.11, 4.85)	87.2	1.192
**Acupuncture**	10	103	8,093	MoCA	2.23 (1.88–2.59)	(1.11, 3.36)	63.9	0.246
**Acupuncture**	2	9	655	LOTCA	12.25 (7.35–17.15)	(−38.45, 62.95)	76.7	9.665
**Acupuncture**	1	8	602	P300 latency	−9.88 (−17.78 to −1.99)	–	–	–
**Acupuncture**	1	8	602	P300 amplitude	1.00 (0.435–1.57)	–	–	–
**Acupuncture + Electrotherapy**	1	2	173	MMSE	4.56 (3.14–5.98)	–	–	–
**Acupuncture + Non-invasive brain stimulation**	2	8	561	MMSE	2.11 (1.09–3.12)	(−0.71, 4.92)	57.3	1.054
**Acupuncture + Non-invasive brain stimulation**	2	10	685	MoCA	2.63 (3.00–3.25)	(1.81, 3.45)	0.0	0.000
**Acupuncture + Non-invasive brain stimulation**	1	5	346	Global cognition (MMSE, MoCA)	3.94 (3.48–4.40)	–	–	–
**Hyperbaric oxygen therapy**	1	1	90	MoCA	2.99 (1.24–4.73)	–	–	–
**Moxibustion**	2	9	686	MMSE	1.95 (1.19–2.70)	(−4.52, 8.42)	23.1	0.111
**Moxibustion**	1	15	1,071	MoCA	2.28 (1.77–2.79)	–	–	–
**Non-invasive brain stimulation**	10	58	3,753	MMSE	2.00 (1.42–2.58)	(−0.17, 4.17)	76.0	0.980
**Non-invasive brain stimulation**	10	93	6,604	MoCA	2.99 (2.77–3.38)	(2.60, 3.38)	9.8	0.023
**Non-invasive brain stimulation**	1	8	520	Total effective rate (difference in MMSE or ADAS-Cog)	4.46 (2.22–6.71)	–	–	–
**Non-invasive brain stimulation**	3	7	343	LOTCA	4.89 (3.15–6.63)	(−9.41, 19.19)	16.4	0.482
**Non-invasive brain stimulation**	3	20	1,584	P300 amplitude	1.35 (1.05–1.64)	(0.98, 1.72)	0.0	0.000
**Non-invasive brain stimulation**	4	22	1,698	P300 latency	19.35 (16.42–22.28)	(15.81, 22.89)	0.0	0.000
**Non-invasive brain stimulation**	1	3	274	ADAS-Cog	2.67 (1.82–3.53)	–	–	–
**Rehabilitation**								
**Cognitive rehabilitation**	5	34	2,351	MMSE	2.54 (1.64–3.44)	(0.08, 5.00)	64.1	0.801
**Cognitive rehabilitation**	5	39	2,900	MoCA	2.94 (2.27–3.64)	(0.86, 5.03)	70.6	0.539
**Cognitive rehabilitation**	2	13	772	LOTCA	18.15 (8.49–27.81)	(−23.62, 59.92)	77.6	69.943
**Exercise**	3	6	515	MoCA	1.78 (0.42–3.13)	(−2.14, 5.69)	66.9	1.508
**Exercise**	1	2	69	ACE	3.91 (1.31–6.51)	–	–	–
**Exercise**	1	1	200	MMSE	1.42 (0.37–2.47)	–	–	–
**Exercise + Cognitive rehabilitation**	1	2	99	MMSE	5.05 (−2.48 to 12.58)	–	–	–
**Exercise + Cognitive rehabilitation**	1	4	167	MoCA	4.95 (2.41–7.48)	–	–	–
**Other rehabilitation therapies**	1	3	99	MMSE	1.8 (−2.65 to 6.34)	–	–	–
**Other rehabilitation therapies**	1	2	67	MoCA	1.5 (0.4–2.6)	–	–	–
**Virtual reality**	6	39	1,833	MMSE	1.50 (0.24–2.75)	(−2.85, 5.84)	87.2	2.039
**Virtual reality**	7	20	905	MoCA	1.58 (0.99–2.17)	(0.51, 2.65)	12.8	0.083
**Virtual reality**	1	5	190	LOTCA	0.25 (0.05–0.45)	–	–	–
**Virtual reality**	1	5	353	P300 amplitude	0.83 (0.41–1.25)	–	–	–
**Virtual reality**	1	5	353	P300 latency	35.29 (27.01–43.57)	–	–	–
**Virtual reality + Cognitive rehabilitation**	1	5	138	MMSE	6.24 (1.02–11.47)	–	–	–
**Virtual reality + Cognitive rehabilitation**	1	3	212	MoCA	5.76 (3.75–7.77)	–	–	–
**Virtual reality + Cognitive rehabilitation**	1	NR	NR	LOTCA	19.46 (0.60–38.31)	–	–	–
**Pharmacological**								
**DL-3-n-Butylphthalide (NBP)**	1	3	228	MMSE	4.89 (4.14–5.63)	–	–	–
**Ginkgo ketone ester (EGB50)**	1	4	316	MMSE	3.26 (1.87–4.64)	–	–	–
**SSRI**	1	4	293	MMSE	1.00 (0.12–1.89)	–	–	–
**Traditional Chinese medicine**	1	20	1,539	MoCA	2.87 (2.11–3.62)	–	–	–
**Traditional Chinese medicine**	1	24	1,903	MMSE	2.56 (2.00–3.12)	–	–	–
**Non-pharmacological**								
**Intensive patient care program**	1	1	242	MMSE	0.80 (−0.14 to 1.7)	–	–	–
**Other**								
**Remote ischemic conditioning**	1	4	153	MMSE	0.58 (0.09–1.06)	–	–	–
**Remote ischemic conditioning**	1	5	201	MoCA	1.73 (1.07–2.39)	–	–	–

Abbreviations: PI = prediction interval; SMD = standardised mean difference.

### Physical modalities alone

Acupuncture alone significantly improved cognition when measured using the MMSE (MD 2.48, 95% CI, 1.93–3.03) and MoCA (MD 2.23, 95% CI, 1.88–2.59) scales, although heterogeneity was high for MMSE (*I*^2^ = 87.2%; τ^2^ = 1.192). Non-invasive brain stimulation demonstrated consistent improvements across MMSE (MD 2.00, 95% CI, 1.42–2.58) and MoCA (MD 2.99, 95% CI, 2.77–3.38) scales with low to moderate heterogeneity. Non-invasive brain stimulation also improved electrophysiological parameters, including P300 amplitude and latency. Moxibustion showed modest but significant improvements when measured on MMSE and MoCA scales.

### Rehabilitation interventions

Cognitive rehabilitation improved cognition when measured using the MMSE (MD 2.54, 95% CI, 1.64–3.44) and MoCA (MD 2.94, 95% CI, 2.27–3.64) scales, with moderate heterogeneity. Exercise demonstrated smaller but significant gains, particularly when measured using the MoCA (MD 1.78, 95% CI, 0.42–3.13). Virtual reality interventions produced modest improvements in cognition when measured using the MMSE (MD 1.50, 95% CI, 0.24–2.75) and MoCA (MD 1.58, 95% CI, 0.99–2.17) scales, although heterogeneity was high for the MMSE. Combined virtual reality and cognitive rehabilitation yielded larger point estimates, though this was based on single reviews.

### Pharmacological interventions

NBP improved cognition by 4.89 points (95% CI, 4.14–5.63) when measured using the MMSE. Traditional Chinese medicine and Ginkgo ketone ester also showed significant improvements on global cognition measures. Selective serotonin reuptake inhibitors demonstrated a modest but significant effect (MD 1.00, 95% CI, 0.12–1.89).

### Combined physical modality and pharmacological interventions

Acupuncture combined with donepezil improved MMSE scores by 1.95 points (95% CI, 1.06–2.84). Acupuncture combined with traditional Chinese medicine showed larger effects on both MMSE (MD 4.27, 95% CI, 3.19–5.34) and MoCA (MD 3.99, 95% CI, 3.09–4.88) scales.

### Combined physical modality and rehabilitation

Acupuncture plus cognitive rehabilitation significantly improved cognition when measured using the MMSE (MD 3.00, 95% CI, 2.19–3.82) and MoCA (MD 2.86, 95% CI, 2.00–3.72) scales, although heterogeneity was substantial (*I*^2^ = 77%-81%). Similar benefits were observed for acupuncture combined with other rehabilitation therapies. Acupuncture plus hyperbaric oxygen therapy showed consistent and precise effects on MMSE (MD 3.73, 95% CI, 3.03–4.43) and MoCA (MD 3.26, 95% CI, 2.54–3.98) scales with minimal heterogeneity.

### Other interventions

Remote ischemic conditioning was associated with small but statistically significant improvements in cognition when measured using the MMSE (MD 0.58, 95% CI, 0.09–1.06) and MoCA (MD 1.73, 95% CI 1.07 to 2.39) scales. An intensive patient care program did not demonstrate a statistically significant effect in improving cognition when measured using the MMSE.

### Adverse events and withdrawals

Thirty-eight SRs reported data on adverse events (see Table S3). Five of these reported no adverse events,^66^^,^^75^^,^^84^^,^^85^^,^^87^ though 3 did not specify whether all included RCTs reported on adverse events.^66^^,^^84^^,^^85^ Thirty-three SRs reported adverse events, though 6 did not specify whether other RCTs found no adverse events or simply did not report data for this outcome.^22^^,^^28^^,^^36^^,^^73^^,^^79^^,^^86^ One SR only reported on pre-specified adverse events of gastrointestinal side effects, bleeding and seizures.^50^ Multiple SRs reported adverse events for acupuncture and non-invasive brain stimulation. Bleeding and syncope were the most reported adverse events following acupuncture. Headache, dizziness, pain and local skin reactions were most reported following non-invasive brain stimulation. Adverse events are further summarised by intervention category in Table S4.

In terms of information on the tolerability of interventions, 17 SRs reported data on withdrawals (see Table S5), though 2 did not specify whether all included RCTs reported on this outcome.^30^^,^^43^ Only 4 SRs reported on both number of withdrawals and reasons.^30^^,^^39^^,^^45^^,^^49^

## Overlap results description

The overlap analysis included 69 SRs and 747 unique primary studies. In total, 1191 study entries were identified when double counting across reviews was allowed. The calculated covered area, defined as N divided by the product of rows and columns, was 2.31%. After adjusting for duplicate counting using the CCA, the value was 0.87%. According to established thresholds, this corresponds to slight overlap. No structural zeros were identified (*X* = 0), and adjustment for structural zeros did not change the CCA (0.87%). At the pairwise level, 2346 possible review pairs were identified, of which 2139 represented actual nodes assessed for overlap. Most review pairs demonstrated slight overlap (<5%), accounting for 1857 comparisons. Moderate overlap (5%–<10%) was observed in 80 pairs, high overlap (10%–< 15%) in 41 pairs, and very high overlap (>15%) in 102 pairs. Overall, these findings indicate that the degree of primary study duplication across included reviews was low, suggesting minimal redundancy and limited risk of bias due to overlapping evidence.

### Risk of bias (AMSTAR 2) of included reviews

Overall confidence in results was high for 2 SRs,^39^^,^^43^ low for 6 SRs,^19^^,^^28^^,^^30^^,^^52^^,^^62^^,^^69^ and critically low for the remaining 61 SRs (see Table 4). Failure to justify language restrictions (search strategy) and to describe excluded studies were the most common reasons for low confidence. In critical domains, 94% of SRs (*n* = 65) used a satisfactory tool to assess risk of bias (judged as yes or partial yes using AMSTAR 2), 74% (*n* = 51) used appropriate statistical methods for meta-analysis, 67% (*n* = 46) conducted satisfactory planning, 65% (*n* = 45) investigated publication bias, 55% (*n* = 38) accounted for individual study risk of bias when discussing results, 35% (*n* = 24) described a comprehensive search strategy, and 6% (*n* = 4) fully described and justified excluded studies. Issues with meta-analytical methods were for studies that did not statistically investigate for sources of heterogeneity (*n* = 18).

**Table 4:** Risk of bias analysis using AMSTAR 2.

SR Ref	AMSTAR 2																
	1. PICO	2. Plann-ing ^a^	3. Study selection	4. Search strat-egy ^a^	5. Duplic-ate study selection	6. Dupli-cate data extraction	7. Exclu-ded studies ^a^	8. Study details	9. Risk of bias ^a^	10. Fund-ing	11. Meta-analy-sis: statis-tics ^a^	12. Meta-analy-sis: RoB	13. Results Interpretation: RoB ^a^	14. Heterogeneity	15. Publication bias ^a^	16. Conflict of Interest	Overall confidence in results of the review (based on critical domains 2, 4, 7, 9, 11, 13, 15)
**Chen et al.^35^**	Y	Y	N	N	N	Y	N	PY	PY	N	Y	Y	Y	Y	N	Y	**Critically low**
**Chen et al.^36^**	Y	N	Y	N	Y	Y	N	PY	Y	N	Y	N	N	Y	Y	N	**Critically low**
**Chen et al.^37^**	Y	N	N	N	Y	N	N	PY	Y	N	Y	N	N	Y	N	Y	**Critically low**
**Daoud et al.^38^**	Y	PY	N	PY	Y	N	N	PY	Y	N	Y	N	Y	Y	N	Y	**Critically low**
**Elsner et al.^39^**	Y	Y	Y	Y	Y	Y	Y	Y	Y	Y	Y	N	Y	Y	Y	Y	**High**
**Fan et al.^40^**	Y	Y	N	N	Y	Y	N	PY	Y	N	Y	N	Y	N	Y	Y	**Critically low**
**Gao et al.^41^**	Y	N	N	N	Y	N	N	Y	Y	N	Y	N	N	N	N	Y	**Critically low**
**Gao et al.^42^**	Y	Y	N	N	Y	Y	N	PY	Y	N	Y	N	Y	Y	N	Y	**Critically low**
**Gibson et al.^43^**	Y	Y	Y	PY	Y	Y	Y	Y	Y	Y	Y	N	Y	Y	Y	Y	**High**
**Gong et al.^44^**	Y	Y	N	N	Y	Y	N	PY	Y	N	N	N	N	N	N	Y	**Critically low**
**Guan et al.^45^**	Y	N	N	PY	Y	Y	N	PY	Y	N	Y	N	Y	Y	Y	N	**Critically low**
**Gunawan et al.^46^**	N	N	N	N	Y	N	N	N	Y	N	N	N	N	N	N	Y	**Critically low**
**Han et al.^47^**	Y	Y	N	N	Y	Y	N	Y	Y	N	Y	N	N	Y	N	Y	**Critically low**
**Han et al.^48^**	Y	N	N	PY	Y	N	N	PY	Y	N	Y	N	Y	Y	Y	Y	**Critically low**
**Hofmeijer et al.^49^**	Y	Y	N	N	Y	N	N	PY	Y	N	Y	N	Y	N	Y	Y	**Critically low**
**Kalbouneh et al.^50^**	Y	PY	Y	N	Y	Y	N	PY	Y	N	Y	N	N	Y	N	Y	**Critically low**
**Khattab et al.^51^**	N	PY	N	N	Y	Y	N	PY	Y	N	Y	N	N	Y	Y	Y	**Critically low**
**Kuang et al.^52^**	Y	Y	N	PY	Y	Y	N	PY	Y	N	Y	N	Y	Y	Y	Y	**Low**
**Li et al.^53^**	Y	Y	N	N	N	Y	N	PY	Y	N	Y	N	N	N	Y	Y	**Critically low**
**Li et al.^19^**	Y	Y	N	PY	Y	Y	N	PY	Y	N	Y	N	Y	Y	Y	Y	**Low**
**Li et al.^20^**	Y	Y	N	PY	Y	Y	N	PY	Y	N	N	N	Y	N	Y	Y	**Critically low**
**Li et al.^21^**	Y	PY	N	N	Y	Y	N	PY	Y	N	N	N	Y	N	Y	Y	**Critically low**
**Lin et al.^54^**	Y	Y	N	PY	Y	Y	N	N	Y	N	Y	N	N	Y	Y	Y	**Critically low**
**Liu et al.^55^**	Y	N	Y	PY	Y	N	N	PY	Y	N	Y	N	N	Y	N	Y	**Critically low**
**Liu et al.^56^**	Y	N	N	PY	Y	Y	N	N	Y	N	N	N	N	N	N	Y	**Critically low**
**Liu et al.^57^**	Y	N	N	N	N	Y	N	PY	Y	N	Y	N	Y	Y	Y	Y	**Critically low**
**Liu et al.^22^**	Y	PY	N	N	Y	Y	N	PY	Y	N	N	N	Y	N	Y	Y	**Critically low**
**Liu et al.^23^**	N	PY	N	PY	Y	Y	N	PY	Y	N	Y	N	N	Y	Y	Y	**Critically low**
**Liu et al.^58^**	Y	Y	N	N	Y	Y	N	PY	Y	N	Y	N	Y	Y	Y	Y	**Critically low**
**Liu et al.^24^**	Y	PY	N	N	Y	Y	N	PY	Y	N	Y	N	Y	Y	Y	Y	**Critically low**
**Li et al.^59^**	Y	PY	N	N	Y	Y	N	N	Y	N	N	N	Y	N	N	Y	**Critically low**
**Li et al.^60^**	Y	N	N	N	N	Y	N	N	Y	N	N	N	N	N	N	Y	**Critically low**
**Li et al.^61^**	Y	PY	N	N	Y	N	N	PY	Y	N	Y	N	N	Y	N	Y	**Critically low**
**Li et al^25^**	Y	PY	N	N	Y	Y	N	PY	Y	N	Y	N	N	Y	Y	Y	**Critically low**
**Luo et al.^62^**	Y	PY	Y	PY	Y	Y	N	PY	Y	N	Y	N	Y	Y	Y	Y	**Low**
**Lyu et al.^63^**	Y	N	Y	PY	Y	Y	N	PY	Y	N	Y	N	N	Y	N	N	**Critically low**
**Ma et al.^64^**	Y	N	N	PY	Y	Y	N	PY	Y	N	Y	N	N	Y	N	N	**Critically low**
**Mingming et al.^65^**	Y	N	N	N	Y	Y	N	N	Y	N	Y	N	Y	Y	N	Y	**Critically low**
**Nie et al.^66^**	Y	N	Y	N	Y	Y	N	PY	Y	N	N	N	N	Y	N	Y	**Critically low**
**Rose Sin Yi et al.^67^**	Y	PY	N	N	Y	Y	N	PY	Y	N	Y	N	Y	Y	Y	Y	**Critically low**
**Shen et al.^68^**	Y	Y	N	N	Y	Y	Y	PY	Y	N	N	N	Y	Y	Y	Y	**Critically low**
**Toh et al.^69^**	Y	Y	N	PY	Y	Y	N	Y	Y	Y	Y	N	Y	Y	Y	Y	**Low**
**Valenzuela-Lopez et al.^26^**	Y	PY	N	N	Y	N	N	PY	Y	N	N	N	N	Y	Y	Y	**Critically low**
**Wang et al.^70^**	Y	N	N	N	Y	Y	N	PY	PY	N	Y	Y	N	Y	N	Y	**Critically low**
**Wang et al.^27^**	Y	Y	Y	N	N	Y	N	PY	Y	N	Y	Y	Y	Y	Y	Y	**Critically low**
**Wang et al.^28^**	Y	PY	N	PY	Y	Y	N	PY	Y	N	Y	N	Y	Y	Y	Y	**Low**
**Wang et al.^71^**	Y	Y	N	PY	Y	N	N	PY	Y	N	Y	N	N	Y	Y	Y	**Critically low**
**Wang et al.^72^**	Y	N	N	N	Y	N	N	PY	Y	N	Y	N	Y	N	Y	Y	**Critically low**
**Wiley et al.^73^**	Y	N	N	N	Y	Y	N	Y	Y	N	N	N	N	N	N	Y	**Critically low**
**Xiao et al.^74^**	Y	PY	N	N	Y	N	N	PY	Y	N	Y	N	Y	N	Y	Y	**Critically low**
**Xiao et al.^29^**	Y	N	N	PY	N	Y	N	PY	Y	N	N	N	Y	N	N	Y	**Critically low**
**Xie et al.^75^**	Y	PY	Y	N	Y	Y	N	PY	Y	N	Y	N	N	Y	Y	Y	**Critically low**
**Xu et al.^30^**	Y	Y	Y	PY	Y	Y	Y	PY	Y	N	Y	N	N	Y	Y	Y	**Low**
**Xu et al.^76^**	Y	N	N	N	N	Y	N	PY	PY	N	N	Y	Y	N	Y	Y	**Critically low**
**Yang et al.^31^**	Y	PY	N	N	Y	Y	N	PY	Y	N	N	N	N	N	Y	Y	**Critically low**
**Yang et al.^77^**	Y	PY	N	N	Y	Y	N	PY	Y	N	Y	N	Y	Y	Y	Y	**Critically low**
**Yang et al.^32^**	Y	PY	N	N	Y	Y	N	PY	Y	N	Y	N	Y	Y	Y	Y	**Critically low**
**Yan et al.^33^**	Y	PY	N	PY	Y	Y	N	PY	Y	N	N	N	N	N	N	Y	**Critically low**
**Yan et al.^78^**	Y	PY	N	N	Y	Y	N	PY	Y	N	Y	N	Y	Y	Y	Y	**Critically low**
**Yikun et al.^79^**	Y	N	N	N	Y	Y	N	PY	Y	N	Y	N	N	N	Y	Y	**Critically low**
**Zhang et al.^81^**	Y	N	Y	N	Y	Y	N	PY	Y	N	Y	N	N	Y	N	Y	**Critically low**
**Zhang et al.^80^**	Y	PY	N	N	Y	Y	N	PY	Y	N	Y	N	Y	Y	Y	Y	**Critically low**
**Zhang et al.^82^**	Y	PY	Y	N	Y	Y	N	N	Y	N	Y	N	Y	Y	Y	Y	**Critically low**
**Zhao et al.^83^**	Y	N	N	PY	N	Y	N	PY	N	N	Y	N	N	Y	Y	Y	**Critically low**
**Zhou et al.^34^**	Y	PY	N	PY	Y	Y	N	PY	Y	N	N	N	N	N	Y	Y	**Critically low**
**Zhou et al.^84^**	Y	N	Y	N	Y	Y	N	PY	Y	N	Y	N	Y	Y	Y	Y	**Critically low**
**Zhou et al.^85^**	Y	Y	N	N	Y	Y	N	Y	Y	N	Y	Y	Y	Y	Y	Y	**Critically low**
**Zhu et al.^86^**	Y	PY	N	N	Y	Y	N	PY	Y	N	Y	N	Y	Y	Y	Y	**Critically low**
**Zhuo et al.^87^**	N	Y	N	PY	Y	Y	N	PY	Y	N	N	N	Y	Y	N	Y	**Critically low**

Abbreviations: N = No; Y = Yes; PY = Partial Yes. ^a^Critical domain. 1. Did the research questions and inclusion criteria for the review include the components of PICO? 2. Did the report of the review contain an explicit statement that the review methods were established prior to the conduct of the review and did the report justify any significant deviations from the protocol? 3. Did the review authors explain their selection of the study designs for inclusion in the review? 4. Did the review authors use a comprehensive literature search strategy? 5. Did the review authors perform study selection in duplicate? 6. Did the review authors perform data extraction in duplicate? 7. Did the review authors provide a list of excluded studies and justify the exclusions? 8. Did the review authors describe the included studies in adequate detail? 9. Did the review authors use a satisfactory technique for assessing the risk of bias (RoB) in individual studies that were included in the review? 10. Did the review authors report on the sources of funding for the studies included in the review? 11. If meta-analysis was performed did the review authors use appropriate methods for statistical combination of results? 12. If meta-analysis was performed, did the review authors assess the potential impact of RoB in individual studies on the results of the meta-analysis or other evidence synthesis? 13. Did the review authors account for RoB in individual studies when interpreting/discussing the results of the review? 14. Did the review authors provide a satisfactory explanation for, and discussion of, any heterogeneity observed in the results of the review? 15. If they performed quantitative synthesis did the review authors carry out an adequate investigation of publication bias (small study bias) and discuss its likely impact on the results of the review? 16. Did the review authors report any potential sources of conflict of interest, including any funding they received for conducting the review? Assessments conducted using the AMSTAR 2 instrument and guidance documents that can be found in the appendices of: Shea BJ, Reeves BC, Wells G, et al. AMSTAR 2: a critical appraisal tool for systematic reviews that include randomised or non-randomised studies of healthcare interventions, or both. BMJ. 2017;358:j4008. doi:10.1136/bmj.j4008.

### SR reported risk of bias for RCTs

55 SRs assessed risk of bias for included RCTs using the Cochrane Collaboration’s Tool for Assessing Risk of Bias (RoB 1) (see Figure S1 and Table S6).^88^ Across RCTs, blinding of participants and personnel and blinding of outcome assessment contributed most to high risk of bias (26% and 11%, respectively). There was limited information leading to predominantly unclear risk of bias in domains of allocation concealment (74%), blinding of outcome assessment (56%), and blinding of participants and personnel (42%). Lower risk of bias was reported for incomplete outcome data (91%), random sequence generation (77%) and selective reporting (76%). RoB 1 data could not be compared for 2 SRs as data were not presented by individual RCT^45^ or domains did not fully align with the RoB 1 tool,^45^^,^^46^^,^^62^^,^^64^ one described different results to presented figures,^86^ one did not include all RCTs in RoB 1 analysis,^62^ and 4 did not report on other bias.^23^^,^^39^^,^^51^^,^^59^ There was significant variability in assessment of the risk of bias for blinding of participants and personnel for single-blinded RCTs. Ten SRs assessed these as being at low risk of bias.^19–22^^,^^25^^,^^28^^,^^32^^,^^48^^,^^58^^,^^80^ Others were split on assessing single-blinded RCTs as unclear or high risk of bias or did not provide this information. One SR graded all RCTs as low risk of bias in this domain as the authors judged blinding to not be possible for the virtual reality intervention.^80^ Nine SRs used RoB 1 to assess overall risk of bias, but there was inconsistency in application of RoB 1 methodology.^38^^,^^46^^,^^52^^,^^53^^,^^56^^,^^62^^,^^69^^,^^71^^,^^77^

Ten SRs used Cochrane Risk of Bias Tool for Randomised Trials (RoB 2) (see Figure S2 and Table S6).^89^ Across RCTs overall risk of bias was low for 20%, some concerns for 62% and high for 18%. For individual domains highest risk of bias was attributed to outcome measurement (11%) and missing outcome data (8%). Some concerns were identified in the randomisation process (50%), selection of the reported result (34%), deviation from intended interventions (31%) and outcome measurement (30%) in particular. Lowest risk of bias was seen in the missing outcome data domain (90%). RoB 2 data could not be compared for one SR that appeared to mix RoB 1 and RoB 2 methodology.^49^ One presented RoB 2 analysis in main figures but described separate RoB 1 analysis in text.^62^

Nine SRs used the Physiotherapy Evidence Database (PEDro) scale to assess RCT quality (see Figure S3 and Table S6).^90^ Seven also used either RoB 1 or RoB 2 to assess risk of bias.^26^^,^^28^^,^^41^^,^^42^^,^^47^^,^^61^^,^^81^ PEDro domain-level data could not be compared for 4 SRs that only reported total scores.^41^^,^^42^^,^^47^^,^^81^ One SR overestimated total scores due to incorrect application of PEDro methodology when calculating this.^61^ Mean total score was 7.4 out of 10 (adjusted for identified SR calculation errors). Issues with blinding of therapists (83%), assessors (60%) and subjects (50%), as well as with allocation concealment (68%) affected methodological quality of RCTs. This may reflect that most of these studies assessed therapy-type interventions where blinding is challenging.

Other tools used included the Review Instrument of Australia’s Joana Briggs Institute (*n* = 1) and Modified Jadad scale scoring system (*n* = 2 and 1 SR used this alone).^74^^,^^76^^,^^91^^,^^92^ One SR did not assess risk of bias.^83^ Of those SRs that assessed publication bias, 19 found evidence that this was possible for some or all global cognition outcomes.

### Reported certainty of evidence

Twenty-four SRs reported GRADE to assess certainty of global cognition outcomes (See Table S7). Ninety-two GRADE assessments were reported for global cognition outcomes for interventions across these SRs. Of these only low-frequency rTMS (a type of non-invasive brain stimulation) was reported by one SR as high certainty,^30^ with the remainder being moderate (*n* = 23), low (*n* = 47) or very low (*n* = 21).

## Discussion

In this synthesis of 69 SRs, including 747 primary studies, most interventions were associated with significant improvements in global cognition although with varying effect sizes. Interventions such as those under the physical modalities, particularly non-invasive brain stimulation and combining interventions seem to show some promise. However, the consistency and certainty of these improvements varied across intervention types and studies suggesting that the magnitude and even the direction of the intervention effects may differ meaningfully across settings or study populations.

In an ageing population with increasing prevalence of stroke, healthcare systems are likely to be significantly impacted by those who experience post-stroke cognitive difficulties. There is therefore an urgent need to identify the most effective strategies to preserve and improve cognitive recovery particularly in this high-risk group. A previous review published in 2020 included 18 SRs of interventions with both global and domain-specific cognitive scores as an outcome (129 RCTs, 7985 participants).^11^ Although we only included SRs with global cognitive outcomes, we still found a large increase in the number of eligible studies (*n* = 69). However, where GRADE was included in the original study, overall confidence in these results were rated as critically low, which was similar to the earlier review where the majority of studies were rated as either low or very low (*n* = 14).^11^ Part of this is down to risk of bias within individual trials for example ensuring blinding participants would be problematic in trials around centred around physical modalities. There may also be variations in comparators/controls or non-standardised doses of the same interventions. Thus, despite the significant increase in the reviews conducted in this area, the degree of primary study duplication was found to be low. This would suggest that despite significant interest in this field the overall quality of the evidence being retrieved is insufficient to be able to influence guidelines and policy at present.

Interventions under our category of physical modalities showed large effects, which translated into meaningful gains in global assessment scores when compared to rehabilitation interventions. Non-invasive brain stimulation (NIBS) was one of the most frequently reported interventions and showed the most precision (in terms of prediction intervals) and consistency by heterogeneity (as measured by *I*^2^). Despite growing evidence for forms of NIBS, such as repetitive TMS, most national clinical guidelines have not recommended their inclusion for either motor or cognitive deficits post-stroke. It is well recognised that stroke affects multiple cognitive domains. However, not all post-stroke patients will develop cognitive impairment. Those who do will likely experience impairment across multiple domains, though the specific domains affected vary between individuals.^93^^,^^94^ A previous umbrella review looking at NIBS in patients with Alzheimer’s Disease and Mild Cognitive Impairment did show that NIBS could significantly improve cognition, including global and executive function, as well as memory.^95^ Therefore, it is perhaps unsurprising that NIBS has also been shown to be effective in people who have experienced stroke when looking at cognitive outcomes, but like other promising therapies, there remains issues around how to standardise treatment, optimise patient selection and challenges in clinical provision.^96^^,^^97^ From a pharmacological perspective NBP alone or when used in combination with other medications seemed to show promising results. NBP is a neuroprotective drug developed in China that has both anti-inflammatory and antioxidant properties.^98^ However, RCTs conducted using NBP are of low methodological quality, small samples and were restricted to China only.^40^ High quality trials conducted in other settings are needed to verify the clinical efficacy for PSCI. Furthermore, any interventions found to be promising need to extend their follow-up to ensure that the gains obtained as part of the intervention period can be maintained in the long-term as evidence for this is currently lacking.

The previous review by Obaid concludes that cognitive rehabilitation improves cognitive function after stroke,^11^ but this conclusion rested on a small evidence based of only 3 reviews, 2 of which assessed^99^^,^^100^ change in a single cognitive domain; only the third that used the MMSE alongside other measures, demonstrated a small improvement in overall cognitive function.^101^ Our review focussed on global cognitive outcomes only. Although we recognise that patients will also experience domain-specific deficits, incident stroke is known to be associated with acute decline in all cognitive domains causing accelerated post-stroke decline in global cognition.^3^ Across all reported global outcome measures (MMSE, MoCA, LOTCA), there was clear and meaningful improvement in cognition across the pooled studies, although reasonable precision was only found in MMSE and MoCA outcomes. Further, across all studies and outcomes, heterogeneity was high. This may be related to the fact that cognitive rehabilitation covers a wide variety of approaches when compared to more standardised protocols, such as for NIBS. The breadth of these interventions will lead to variability in terms of the intensity, content, modality of delivery and likely variable adherence to these interventions. Although we did not separate out the intervention effects on stroke subtype there has been some evidence that cognitive training has a stronger effect in ischaemic stroke patients compared to haemorrhagic stroke patients.^102^ Our study focused on global cognition as a primary outcome. However, there is evidence that innovative approaches to rehabilitation such as dual-task training have been found to enhance cognitive functions, particularly attention and executive function.^103^ Cognitive-motor dual task training for PSCI has also been shown to improve global cognition, which has been supported by improved neural efficiency of associated brain areas.^104^ Further, to measure the true impact of any intervention addressing PSCI, secondary holistic outcome measures of, for example, functional outcomes will also be beneficial and should be included in future RCTs.

Since the previous review, there has been an increase in multicomponent interventions. When combinations of interventions were used, for example, acupuncture plus cognitive rehabilitation, an additive effect was observed through even greater improvements in cognitive scores. At present, there is some more consistent evidence in favour of such non-pharmacological multicomponent interventions compared to for example pharmacological plus non-pharmacological multicomponent interventions based on our review. However, we do note that there were fewer studies of this type and there would be more scope in future to assess different combinations of intervention. This is because although stroke is itself a focal vascular lesion, cognitive impairment post-stroke is not only localised to the focal area of damage. Rather, neuroimaging studies have shown that these impairments can be the result from disturbances in brain network connectivity.^94^ Further, cognitive recovery following a stroke can be compounded by individual factors such as age, ethnicity and pre-morbid cognitive performance^105^ as well as hospital-related complications such as delirium and infection.^7^^,^^8^ As such, it may be that interventions targeting different areas and domains could lead to greater clinical benefit. There is certainly some evidence pointing towards benefits of multicomponent interventions, not only for general cognitive functioning but also domain-specific outcomes such as memory.^106^ The FINGER RCT, which tested a multicomponent intervention (diet, physical activity, cognitive training and social activities and cardiovascular risk factor management) on cognition on those at-risk of developing dementia, did find group differences for executive functioning and processing speed but not for memory.^107^ However, this may depend on the exact nature of the interventions. A previous pooled patient-level analysis to test the efficacy of multidomain interventions targeting risk factors did not have a significant effect on executive function but did find indications for better scores in visual attention and processing speed.^108^ However, it should be noted that only 2 trials of a small number of patients with mild stroke were included. There is broad consensus, however, that any disease modifying strategy for PSCI needs to be multimodal based on both pharmacological and non-pharmacological strategies, and that there also needs to be better stratification of patients.^109^

The strengths of this study include the broad, inclusive search strategy to capture SRs of any type of intervention regardless of language and building on the findings of the previous review.^11^ Recognising that global cognitive difficulties may be readily detected in clinical settings, we focused only on SRs that had any global cognitive outcome as their primary outcome. This is because no single cognitive score at present is recommended above others to detect PSCI.

We do recognise several limitations. Firstly, this study does not include reviews of interventions that included domain-specific cognitive outcomes. This was intentional to allow for a clear synthesis of the findings with one outcome in mind rather than individual domains. Secondly, we recognise that an umbrella review has the potential to generate overlap when synthesising results particularly if the original SRs had overlapping category definitions (in part due to combined treatments), inclusion or exclusion criteria. To account for this, we assessed overlap by calculating the CCA and found that the degree of primary study duplication across included reviews was low. An additional limitation is the use of AMSTAR 2 tool for risk of bias assessment as it is not designed for NMAs, which were included in the meta-analysis. Many of the primary studies included in the SR were conducted in East Asia. This could influence the generalisability of some of the more region-specific interventions depending on the availability of the for example pharmacological intervention as well as the health care systems they are integrated into. Further, we do accept that categorising the interventions into predefined areas can create some of the heterogeneity we are seeing. Finally, we did not stratify the results by stroke subtype nor stroke recurrence. Although this means that our findings should be treated with caution and may not be applicable to every stroke survivor, the overall sample does reflect the significant heterogeneity among the clinical stroke population and the challenges clinical teams will face in deciding how best to treat individuals to reduce the impact of post-stroke cognitive decline.

Despite the increase in primary and evidence synthesis studies in this area, the methodological limitations remain which makes recommendations to guidelines challenging despite some positive results. Future trials should focus on trying to better define their patient population. This could for example be achieved through risk stratification, eg, with risk prediction tools^110^ for PSCI rather than single measures such as stroke severity. Importantly, to avoid bias, pre-stroke cognitive impairment/dementia should be excluded at baseline. As with any trial looking at cognition as an outcome, there needs to be clarity and a well-defined, patient-centred clinically meaningful change in the outcome measure. This may well include qualitative feedback as well as differences in objective scores. Finally, the intervention, particularly if multimodal, needs to be clearly defined in terms of frequency, duration and with appropriate follow-up duration.

In summary, the number of RCTs in post-stroke cognition where global cognition is measured has increased significantly in less than a decade. There continues to be emerging promising evidence around interventions such as NIBS, NBP and combining modalities, which do result in meaningful improvements in global cognitive scores. Unfortunately, methodological limitations continue to predominate across studies. Particularly when the populations these interventions are tested in can vary and be heterogenous in terms of for example stroke severity. To try to better standardise the included populations there could be opportunities for risk stratification in multimodal interventions. It may also be that there could be focus on high-quality RCTs that looks to integrate a variety of different rehabilitation types eg, cognitive, motor and sensorimotor interventions but also accounting for some of the clinical confounders that can affect cognitive recovery.

## Supplementary Material

Supplementary_material_aakag122

## Data Availability

All data for this review are contained within the manuscript.
