# Supplementary material for: Interventions to reduce cognitive decline post-stroke: an umbrella review
Source: Eur Stroke J. 2026 Oct 16;11(10):aakag122. doi: 10.1093/esj/aakag122 (PMC13674605; doi:10.1093/esj/aakag122)
Supplement: Supplementary file 1 [file supplementary_material_aakag122.zip › Supplementary Material_Final.docx]

**Supplementary Material**

**Supplementary Table 1: Definition of Each Intervention Category**

| **Intervention type** | **Definition** |
| --- | --- |
| Acupuncture | Nonpharmacological treatment whereby needles or pressure are applied to specific points on the body. |
| Cognitive rehabilitation | Specialised rehabilitation therapy involving cognitive training that is designed to improve the ability to complete cognitive tasks. |
| Electrotherapy | Non-invasive application of electrical and electromagnetic therapies, generally in physiotherapy practice e.g. ultra short-wave electrotherapy, transcutaneous electrical nerve stimulation (TENS). |
| Exercise | Any physical activity delivered as part of a structured regimen. |
| Hyperbaric oxygen therapy | Delivery of pure oxygen at increased pressures within an enclosed space. |
| Intensive patient care program | Intensive patient care program described by one RCT comprising psychoeducation and psychonursing, cognitive rehabilitation and mobile communication application. |
| Moxibustion | Therapy whereby dried mugwort is burned on specific points on the body. |
| Non-invasive brain stimulation | Non-invasive use of electrical currents (transcranial electrical stimulation), magnetic fields (transcranial magnetic stimulation), light (transcranial photobiomodulation) and/or ultrasound (transcranial ultrasound stimulation) to modulate brain activity. |
| Non-invasive ventilation | Delivery of pressurised gas to aid in respiratory support via an external system (without endotracheal intubation). |
| Non-pharmacological | Intervention not involving drug treatment. |
| Other rehabilitation therapies | Rehabilitation therapies not otherwise categorised including occupational and robot-assisted therapy. |
| Pharmacological | Intervention involving drug treatment. |
| Physical modality | Interventions involving the application of different forms of energy designed to stimulate healing and recovery (e.g. thermal, electrical, etc.). |
| Rehabilitation | Rehabilitation therapies that do not involve an intervention type otherwise described (e.g. cognitive rehabilitation, exercise, virtual reality, etc.). |
| Remote ischemic conditioning | Brief reversible interruption of blood flow to tissue e.g. by inflation and deflation of a blood pressure cuff. |
| SSRI | Selective serotonin reuptake inhibitors |
| Traditional Chinese medicine | Prescriptions containing herb(s), supplement(s) and/or Chinese patent medicines not used in conventional western medicine. |
| Virtual reality | Rehabilitation therapy involving the use of computer-based simulation enabling interaction with artificial reality. |

**Supplementary Table 2. Treatment duration and follow-up by intervention type.**

| **Intervention type group** | **Intervention type** | **RCTs reporting treatment duration** | **Treatment duration (weeks)** | **RCTs reporting follow-up** | **Follow up (weeks)** |
| --- | --- | --- | --- | --- | --- |
|  |  | n (%) | mean (range) | n (%) | mean (range) |
| Combined (Physical modality + Other) | Acupuncture + Hyperbaric oxygen therapy | 10 (100%) | 10.93 (2.1-8) | 0 (0%) | NR |
| Combined (Physical modality + Pharmacological) | Acupuncture + Donepezil | 6 (100%) | 5.67 (4-12) | 0 (0%) | NR |
| Combined (Physical modality + Pharmacological) | Acupuncture + Traditional Chinese medicine | 5 (100%) | 6.2 (3-12) | 0 (0%) | NR |
| Combined (Physical modality + Rehabilitation) | Acupuncture + Cognitive rehabilitation | 44 (100%) | 6.2 (3-12) | 1 (2.27%) | 48 (48-48) |
| Combined (Physical modality + Rehabilitation) | Acupuncture + Rehabilitation therapies | 15 (100%) | 6.67 (4-12) | 1 (6.67%) | 48 (48-48) |
| Combined (Physical modality + Rehabilitation) | Non-invasive brain stimulation + Cognitive rehabilitation | 6 (100%) | 3.5 (2-4) | 0 (0%) | NR |
| Non-pharmacological | Cognitive rehabilitation / Exercise / Physical modality therapies | 7 (100%) | 14 (8-24) | 0 (0%) | NR |
| Non-pharmacological | Intensive patient care program | 0 (0%) | NR | 0 (0%) | NR |
| Other | Non-invasive ventilation | 9 (100%) | 11.89 (3-24) | 3 (33.33%) | 22.67 (8-36) |
| Other | Remote ischemic conditioning | 0 (0%) | NR | 0 (0%) | NR |
| Pharmacological | DL-3-n-Butylphthalide (NBP) | 24 (100%) | 12.21 (2-24) | 0 (0%) | NR |
| Pharmacological | DL-3-n-Butylphthalide (NBP) + Citicoline | 6 (100%) | 5.55 (1.32-24) | 0 (0%) | NR |
| Pharmacological | DL-3-n-Butylphthalide (NBP) + Donepezil | 2 (100%) | 24 (24-24) | 0 (0%) | NR |
| Pharmacological | DL-3-n-butylphthalide (NBP) + Edavarone | 1 (100%) | 2 (2-2) | 0 (0%) | NR |
| Pharmacological | DL-3-n-butylphthalide (NBP) + Galanthamine | 1 (100%) | 12 (12-12) | 0 (0%) | NR |
| Pharmacological | DL-3-n-butylphthalide (NBP) + Huperzine A | 1 (100%) | 2.68 (2.68-2.68) | 0 (0%) | NR |
| Pharmacological | DL-3-n-butylphthalide (NBP) + Idebenone | 1 (100%) | 8 (8-8) | 0 (0%) | NR |
| Pharmacological | DL-3-n-Butylphthalide (NBP) + Nimodipine | 4 (100%) | 9 (4-12) | 0 (0%) | NR |
| Pharmacological | DL-3-n-Butylphthalide (NBP) + Oxiracetam | 14 (100%) | 8.71 (2-12) | 0 (0%) | NR |
| Pharmacological | DL-3-n-butylphthalide (NBP) + Piracetam | 3 (100%) | 6 (2-12) | 0 (0%) | NR |
| Pharmacological | DL-3-n-butylphthalide (NBP) + Urinary kallidinogenase | 3 (100%) | 4 (4-4) | 0 (0%) | NR |
| Pharmacological | Ginkgo ketone ester (EGB50) | 4 (100%) | 18.93 (2-25.71) | 0 (0%) | NR |
| Pharmacological | SSRI | 0 (0%) | NR | 0 (0%) | NR |
| Pharmacological | Traditional Chinese medicine | 34 (100%) | 10.06 (2-24) | 0 (0%) | NR |
| Physical modality | Acupuncture | 209 (98.58%) | 6.12 (1.71-17.14) | 1 (0.47%) | 48 (48-48) |
| Physical modality | Acupuncture + Electrotherapy | 2 (100%) | NR | 0 (0%) | NR |
| Physical modality | Acupuncture + Non-invasive brain stimulation | 15 (100%) | 5.31 (2.9-12) | 1 (6.67%) | 10 (10-10) |
| Physical modality | Hyperbaric oxygen therapy | 0 (0%) | NR | 0 (0%) | NR |
| Physical modality | Moxibustion | 17 (94.44%) | 6.59 (4-12) | 0 (0%) | NR |
| Physical modality | Non-invasive brain stimulation | 184 (92.46%) | 4.45 (1-12) | 11 (5.53%) | 6.45 (3-12) |
| Physical modality | Physical modality therapies | 17 (100%) | 6.53 (1.43-24) | 0 (0%) | NR |
| Rehabilitation | Cognitive rehabilitation | 78 (86.67%) | 7.36 (1.43-48) | 3 (3.33%) | 18.67 (4-48) |
| Rehabilitation | Exercise | 54 (93.1%) | 15.41 (2-72) | 3 (5.17%) | 26.67 (16-40) |
| Rehabilitation | Exercise + Cognitive rehabilitation | 4 (100%) | 10.5 (4-18) | 0 (0%) | NR |
| Rehabilitation | Other rehabilitation therapies | 15 (100%) | 5.42 (1.43-18) | 3 (20%) | 21.33 (4-48) |
| Rehabilitation | Virtual reality | 55 (93.22%) | 5.94 (2-24) | 6 (10.17%) | 7.67 (4-18) |
| Rehabilitation | Virtual reality + Cognitive rehabilitation | 7 (100%) | 5.86 (3-12) | 0 (0%) | NR |

NR = not reported.

**Supplementary Table 3: Details of adverse events (AEs) reported by included systematic reviews.**

| **Systematic review paper** | **Intervention categories** | **No of primary studies (all outcomes)** | | | | **No of participants in RCTs that reported on AEs** | | | **Adverse event details (no of events / no of RCTs)** | | **Meta-analysis** | **Additional information** |
| --- | --- | --- | --- | --- | --- | --- | --- | --- | --- | --- | --- | --- |
|  |  | **Total** | **Reporting on AEs** | **No AEs** | **AEs** | **I** | **C** | **Total** | **I** | **C** |  |  |
| Chen 2022 (1) | Non-invasive brain stimulation | 25 | 21 | 17 | 4 | 232 | 224 | 456 | Mild headache (4 / 2 RCTs); mild neck pain (2 / 1 RCT); anxiety (1 / 1 RCT); sleep disorders (1 / 1 RCT); scalp discomfort (1 / 1 RCT) | Mild headache (2 / 2 RCTs); neck pain (2 / 1 RCT); sleep disorders (1 / 1 RCT); fatigue (1 / 1 RCT) |  |  |
| Chen 2022 (2) | Virtual reality | 21 | NR | NR | 1 | NR | NR | NR | Mild dizziness (1 / RCT) | None |  | Unclear whether all RCTs reported adverse event data. |
| Chen 2024 | Non-invasive brain stimulation | 51 | 15 | 5 | 10 | NR | NR | NR | Mild dizziness and headache (7 RCTs); palpitations (1 / 1 RCT); inattention (1 / 1 RCT); insomnia (1 / 1 RCT); nausea (2 / 2 RCTs); abdominal discomfort (4 / 2 RCTs) | Nausea (1 / 1 RCT); abdominal discomfort (1 / 1 RCT) |  |  |
| Daoud 2024 | Non-invasive brain stimulation | 6 | 6 | 5 | 1 | 163 | 162 | 325 | Sneezing (1 / 1 RCT) | None |  | No SAEs. GRADE for Number of adverse events = moderate certainty (downgraded for imprecision as risk in intervention group is based on the assumed risk in the comparison group and the relative effect of the intervention). |
| Elsner 2020 | Non-invasive brain stimulation | 67 | 36 | NR | NR | NR | NR | NR | Skin redness at site (12 / 2 RCTs); mild headache (8 / 2 RCTs); sleepiness (1 / 1 RCT); migraine (1 / 1 RCT); tingling sensation of unaffected hand (1 / 1 RCT); moderate-severe AEs not fully described (3 / 1 RCT); pain, burning or headache (6 / 1 RCT); mild tingling sensation (3 / 1 RCT) | Skin redness at site (3 / 1 RCT); mild headache (3 / 1 RCT); phosphene (1 / 1 RCT); abdominal pain (1 / 1 RCT); retching (1 / 1 RCT); transient aching or burning sensations (6 / 1 RCT); moderate-severe AEs not fully described (3 / 1 RCT) | "Dropouts, adverse events and deaths during intervention period": Intervention vs passive control: I: 45 events, C: 20 events. RR = 1.25 (0.74, 2.13), I^2^ = 1%. Intervention vs active control: I: 5 events, C: 2 events. RR = 1.76 (0.43, 7.17), I^2^ = 0%. |  |
|  |  |  |  |  |  |  |  |  | Dizziness (1 / 1 RCT); headache (1 / 1 RCT); mild AEs not fully described (10 / 1 RCT) | |  |  |
| Fan 2022 | All (DL-3-n-Butylphthalide (NBP) + Oxiracetam, DL-3-n-Butylphthalide (NBP) + Nimodipine, DL-3-n-Butylphthalide (NBP) + Donepezil, DL-3-n-Butylphthalide (NBP) + Citicoline, DL-3-n-Butylphthalide (NBP)) | 26 | 17 | 3 | 14 | 800 | 792 | 1592 | Unspecified (45 / 14 RCTs) | Unspecified (79 / 14 RCTs) | RR = 0.57 (0.41, 0.80); I^2^ = 0% | Details of individual AEs not reported in SR but included elevated transaminase, gastrointestinal discomfort, nausea and vomiting, anorexia, dizziness, insomnia and decreased blood pressure. |
|  | DL-3-n-Butylphthalide (NBP) | 10 | 7 | 2 | 5 | 269 | 268 | 537 | Unspecified (16 / 3 RCTs) | Unspecified (42 / 5 RCTs) |  |  |
|  | DL-3-n-Butylphthalide (NBP) + Citicoline | 2 | 0 | N/A | N/A | N/A | N/A | N/A | N/A | N/A |  |  |
|  | DL-3-n-Butylphthalide (NBP) + Donepezil | 1 | 0 | N/A | N/A | N/A | N/A | N/A | N/A | N/A |  |  |
|  | DL-3-n-Butylphthalide (NBP) + Nimodipine | 3 | 2 | 0 | 2 | 89 | 88 | 177 | Unspecified (4 / 2 RCTs) | Unspecified (3 / 2 RCTs) |  |  |
|  | DL-3-n-Butylphthalide (NBP) + Oxiracetam | 10 | 8 | 1 | 9 | 442 | 436 | 878 | Unspecified (25 / 7 RCTs) | Unspecified (34 / 7 RCTs) |  |  |
| Gao 2023 | Non-invasive brain stimulation + Cognitive rehabilitation | 8 | 4 | 1 | 3 | 62 | 60 | 122 | Dizziness (2 RCTs); headache (2 RCTs); scalp pain (2 / 1 RCT) | Headache (1 / 1 RCT); dizziness (2 / 1 RCT) |  |  |
| Gong 2023 | Non-invasive brain stimulation | 11 | 10 | 6 | 4 | 202 | 195 | 397 | Dizziness (2 RCTs); headache (2 RCTs); anxiety (1 RCT); tiredness (1 RCT); sneezing (1 RCT) | |  | No withdrawals due to AEs. |
| Guan 2021 | Ginkgo ketone ester (EGB50) | 16 | 8 | 5 | 3 | 520 | 508 | 1028 | Vascular pain, prolonged coagulation time, gastrointestinal AEs (3 RCTs) | |  |  |
| Han 2023 | Non-invasive brain stimulation | 17 (excluding PCTs) | 9 | 3 | 6 | 216 | 219 | 435 | Dizziness or headache (3 RCTs); sneezing (1 RCT); inattention or sleep disorder (1 RCT); seizure (1 RCT) | |  |  |
| Han 2024 | Acupuncture | 39 | 6 | 0 | 6 | 272 | 271 | 543 | Haematoma (9 / 4 RCTs); syncope (3 / 3 RCTs); pain (5 / 2 RCTs); stroke (1 / 1 RCT); pruritus (1 / 1 RCT); nausea (1 / 1 RCT); bleeding (3 / 1 RCT); influenza (1 / 1 RCT) | Nausea (4 / 2 RCTs); pain (1 / 1 RCT) |  | 1 withdrawal from treatment group due to AE of influenza. 1 withdrawal from control group due to AE of nausea. Note text does not match tabular data - tabular data extracted. |
| Kalbouneh 2022 | SSRI | 44 | NR | NR | NR | NR | NR | NR | Gastrointestinal AEs (20 RCTs); bleeding (10 RCTs); seizures (8 RCTs) | | Seizures: RR = 1.44 (1.13, 1.83) I^2^ = 0%. Bleeding: RR = 0.92 (0.76, 1.12) I2 = 0%. Gastrointestinal AEs: 1.29 (1.00, 1.67) I^2^ = 80%. | Specifically reported on SEs judged to be of special interest for SSRIs: gastrointestinal SEs, bleeding and seizures. No information on other AEs. |
| Khattab 2021 | Exercise | 17 | 12 | 8 | 4 | 249 | 233 | 482 | Fall (2 / 2 RCTs); hypertension (1 / 1 RCT); death (2 / 1 RCT) | |  | Unclear whether reported deaths were related to intervention. |
| Kuang 2021 | Acupuncture | 28 | 1 | 0 | 1 | 94 | 94 | 188 | Haematoma (11 / 1 RCT), syncope (3 / 1 RCT) | None |  |  |
| Liu 2023 (1) | Moxibustion | 18 | 3 | 1 | 2 | 94 | 93 | 187 | Mild AEs (7 / 1 RCT); pain (3 / 1 RCT); new-onset epilepsy (8 / 1 RCT) | Mild AEs (4 / 1 RCT) |  | Majority of AEs (pain, epilepsy) were from single RCT which was only one to use scarring moxibustion. 3 withdrew from this trial due to pain AE. |
| Liu 2020 | Acupuncture | 22 | 9 | 6 | 3 | 882 | | | Unspecified AE (1 / 1 RCT); haematoma (3 / 2 RCTs); syncope (1 / 1 RCT) | Syncope (3 / 1 RCT) |  |  |
| Liu 2024 (1) | Non-invasive brain stimulation | 74 | NR | NR | 16 | NR | NR | NR | Mild headache or dizziness (32); scalp discomfort (4); nausea and vomiting (3); sneezing (1); difficulty sleeping (1) | |  | Unclear whether all RCTs reported adverse event data. |
| Liu 2023 (2) | Acupuncture | 38 | 3 | 1 | 2 | 74 | 71 | 145 | Subcutaneous oedema and syncope (3 / 1 RCT); subcutaneous oedema (3 / 1 RCT) | None | RR = 6.83 (0.86, 54.13); I^2^ = 0%. |  |
| Liu 2023 (3) | All (Acupuncture, Acupuncture + Cognitive rehabilitation) | 62 | 6 | 3 | 3 | 344 | 288 | 632 | Haematoma (1 RCT); dizziness, pallor, sleepiness (2 RCTs) | None |  |  |
|  | Acupuncture | 30 | 5 | 3 | 2 | 304 | 250 | 554 | Dizziness, pallor, sleepiness (2 RCTs) | None |  |  |
|  | Acupuncture + Cognitive rehabilitation | 38 | 4 | 1 | 3 | 238 | 182 | 420 | Haematoma (1 RCT); dizziness, pallor, sleepiness (2 RCTs) | None |  |  |
| Li 2022 (3) | All (Virtual reality, Cognitive rehabilitation, Non-invasive brain stimulation, Acupuncture) | 55 | 7 | 3 | 4 | 245 | 245 | 490 | Mild headache and dizziness (4 / 2 RCTs); itching tingling and burning at site (1 RCT); haematoma (3 / 1 RCT) | None |  |  |
|  | Acupuncture | 18 | 1 | 0 | 1 | 30 | 30 | 60 | Haematoma (3 / 1 RCT) | None |  |  |
|  | Cognitive rehabilitation | 12 | 1 | 1 | 0 | 30 | 30 | 60 | None | None |  |  |
|  | Non-invasive brain stimulation | 16 | 4 | 1 | 3 | 175 | 175 | 350 | Mild headache and dizziness (4 / 2 RCTs); itching tingling and burning at site (1 RCT) | None |  |  |
|  | Virtual reality | 12 | 1 | 1 | 0 | 10 | 10 | 20 | None | None |  |  |
| Luo 2024 | Acupuncture | 29 | NR | NR | 2 | NR | NR | NR | Unspecified AE (1 / 1 RCT); erythema and pruritus at site (1 / 1 RCT) | Unspecified AE (2 / 1 RCT) |  | Unclear whether all RCTs reported adverse event data. |
| Lyu 2023 | Non-invasive brain stimulation | 22 | 5 | 2 | 3 | 189 | 101 | 290 | Headache or erythema at site (4 / 1 RCT); mild headache (1 / 1 RCT); pricking (1 / 1 RCT); erythema (2 / 1 RCT); erythema and blistering (1 / 1 RCT); dizziness (1 / 1 RCT) | Headache or erythema at site (2 / 1 RCT); pricking (1 / 1 RCT) |  |  |
| Nie 2021 | Cognitive rehabilitation | 32 | NR | NR | NR | NR | NR | NR | None | None |  | Unclear whether all RCTs reported adverse event data. |
| Shen 2022 | Traditional Chinese medicine | 34 | 23 | 11 | 12 | 910 | 909 | 1819 | All AEs (47 / 12 RCTs); gastrointestinal AEs including nausea, vomiting, constipation, stomach discomfort, loss of appetite (12 RCTs); nervous system AEs including drowsiness, insomnia, dizziness, fatigue (5 RCTs); other AEs including dry mouth, frequent urination, rash, BP fluctuations (4 RCTs) | All AEs (55 / 12 RCTs); gastrointestinal AEs including nausea, vomiting, constipation, stomach discomfort, loss of appetite (8 RCTs); nervous system AEs including drowsiness, insomnia, dizziness, fatigue (6 RCTs); other AEs including dry mouth, frequent urination, rash, BP fluctuations (6 RCTs) | RR = 0.86 (0.60, 1.23); I^2^ = 0% | Funnel plot showed evidence of publication bias for this outcome. |
| Wang 2023 | Non-invasive brain stimulation | 20 | 15 | 9 | 6 | 226 | 231 | 457 | Mild headaches and discomfort (4 / 1 RCT); headache or dizziness (1 RCT); headache (2 / 2 RCTs); dizziness (1 / 1 RCT); anxiety (1 / 1 RCT) | Mild headaches and discomfort (2 / 1 RCT); headache (2 / 2 RCTs); dizziness (2 / 1 RCT); tiredness (1 / 1 RCT); discomfort at site (5 / 1 RCT) |  |  |
|  |  |  |  |  |  |  |  |  | Headache (6 / 1 RCT); aching/burning sensation (6 / 1 RCT); erythema at site (3 / 1 RCT); insomnia (1 / 1 RCT) | |  |  |
| Wang 2022 (2) | Non-invasive brain stimulation | 18 | NR | NR | 6 | NR | NR | NR | Transient dizziness, pain, pins and needles, sneezing (6 RCTs) | |  | Unclear whether all RCTs reported adverse event data. |
| Wiley 2022 | Virtual reality | 8 | NR | NR | 1 | 17 | 14 | 31 | None | Unspecified AE (1 / 1 RCT) |  | Unclear whether all RCTs reported adverse event data. |
| Xie 2023 | Non-invasive brain stimulation | 5 | 1 | 1 | 0 | 12 | 12 | 24 | None | None |  |  |
| Xu 2023 | All (Non-invasive brain stimulation, Acupuncture) | 90 | 32 | NR | NR | 2354 | | | Leading to treatment discontinuation (30): respiratory infection (2); dizziness (1); haemorrhagic cerebral infarction (1); chronic hydrocephalus (1); ischaemic stroke (1); subdural haematoma following fall (1); epilepsy (1); change in condition (4); personal reasons (18). Not leading to treatment discontinuation (87): epilepsy (1); nausea and vomiting (22); abdominal discomfort (6); diarrhoea (11); erythema (5); insomnia (4); fever (4); headache, dizziness (33); lack of concentration (1) | |  | Unable to extract AE data by intervention category. Note "personal reasons" reported as AE leading to treatment discontinuation which would be more in-keeping with reason for withdrawal. |
|  | Acupuncture | 12 | NR | NR | NR | NR | NR | NR | Unclear | Unclear |  |  |
|  | Non-invasive brain stimulation | 78 | NR | NR | NR | NR | NR | NR | Unclear | Unclear |  |  |
| Xu 2022 | Non-invasive brain stimulation | 10 | 5 | 3 | 2 | 194 | 195 | 389 | Unspecified (3 / 2 RCTs) | Unspecified (3 / 2 RCTs) | OR = 1.03 (0.20, 5.32); I^2^ = 0%. | Funnel plot showed evidence of publication bias. |
| Yang 2024 (2) | Non-invasive brain stimulation | 29 | 16 | 6 | 10 | 431 | 426 | 857 | Mild dizziness and headache (7 RCTs); insomnia and nausea (1 RCT); sneezing (1 RCT); anxiety (1 RCT) | |  | No SAEs |
| Yan 2024 | Non-invasive brain stimulation | 26 | 15 | 11 | 4 | 357 | 224 | 581 | Headache (5 / 2 RCTs); tingling (2 / 1 RCT); dizziness (1 / 1 RCT); dizziness/headache (1 RCT); prickling sensation (1 RCT) | |  | No SAEs |
| Yikun 2023 | Non-invasive brain stimulation | 16 | NR | NR | 4 | NR | NR | NR | Mild headache/dizziness (4 RCTs) | |  | Unclear whether all RCTs reported adverse event data. |
| Zhao 2024 | Non-invasive brain stimulation | 23 | 14 | 9 | 5 | 320 | 283 | 603 | Anxiety (1 RCT); fatigue (1 RCT); headache (2 RCT); dizziness (1 RCT); sneezing (1 RCT); unspecified mild AEs (1 RCT) | |  |  |
| Zhou 2020 | Acupuncture | 37 | NR | NR | NR | NR | NR | NR | None | None |  | Unclear whether all RCTs reported adverse event data. |
| Zhou 2022 | Cognitive rehabilitation | 17 | NR | NR | NR | NR | NR | NR | None | None |  | Unclear whether all RCTs reported adverse event data. |
| Zhu 2024 | Non-invasive brain stimulation | 10 | NR | NR | 4 | NR | NR | NR | Discomfort (1 RCT); headaches or dizziness (3 RCTs) | Headaches or dizziness (3 RCTs) |  | Unclear whether all RCTs reported adverse event data. |
| Zhuo 2023 | Acupuncture + Rehabilitation therapies | 18 | 2 | 2 | 0 | 124 | 123 | 247 | None | None |  |  |

**AE = adverse event; RCT = randomised controlled trial; I = intervention group; C = control group; NR = not reported; SAE = serious adverse event.**

**Supplementary Table 4. Details of adverse events (AEs) summarised by intervention type**

| **Intervention type** | **Adverse event** | **Adverse event details ^a^** | **No of SRs** | **No of RCTs ^b^** | **No of events** | | |
| --- | --- | --- | --- | --- | --- | --- | --- |
|  |  |  |  |  | **I** | **C** | **Total across all groups ^c^** |
| **Acupuncture** | | | **7** | **17** | **54 *** | **10 *** | **64 *** |
|  | Bleeding | Bleeding, haematoma | 4 | 9 | 29 | 0 | 29 |
|  | Syncope |  | 3 | 5 | 7 | 3 | 10 |
|  | Pain |  | 1 | 2 | 5 | 1 | 6 |
|  | Nausea |  | 1 | 2 | 1 | 4 | 5 |
|  | Local skin reaction | Erythema and pruritus at site, Subcutaneous oedema | 2 | 2 | 4 | 0 | 4 |
|  | Unspecified |  | 2 | 2 | 2 | 2 | 4 |
|  | Local skin reaction and syncope | Subcutaneous oedema and syncope | 1 | 1 | 3 | 0 | 3 |
|  | Abnormal sensation | Pruritus | 1 | 1 | 1 | 0 | 1 |
|  | Respiratory infection | Influenza | 1 | 1 | 1 | 0 | 1 |
|  | Stroke |  | 1 | 1 | 1 | 0 | 1 |
|  | Dizziness, pallor, sleepiness |  | 1 | 2 | NR | NR | NR |
| **Acupuncture + Cognitive rehabilitation** | | | **1** | **3** | **NR** | **NR** | **NR** |
|  | Dizziness, pallor, sleepiness |  | 1 | 2 | NR | NR | NR |
|  | Bleeding | Haematoma | 1 | 1 | NR | NR | NR |
| **DL-3-n-Butylphthalide (NBP)** | | | **1** | **5** | **16** | **42** | **58** |
|  | Unspecified |  | 1 | 5 | 16 | 42 | 58 |
| **DL-3-n-Butylphthalide (NBP) + Nimodipine** | | | **1** | **2** | **4** | **3** | **7** |
|  | Unspecified |  | 1 | 2 | 4 | 3 | 7 |
| **DL-3-n-Butylphthalide (NBP) + Oxiracetam** | | | **1** | **9** | **25** | **34** | **59** |
|  | Unspecified |  | 1 | 9 | 25 | 34 | 59 |
| **Exercise** | | | **1** | **4** | **NR** | **NR** | **5** |
|  | Fall |  | 1 | 2 | NR | NR | 2 |
|  | Death |  | 1 | 1 | NR | NR | 2 |
|  | Hypertension |  | 1 | 1 | NR | NR | 1 |
| **Ginkgo ketone ester (EGB50)** | | | **1** | **3** | **NR** | **NR** | **NR** |
|  | Vascular pain, prolonged coagulation time, gastrointestinal AEs |  | 1 | 3 | NR | NR | NR |
| **Moxibustion** | | | **1** | **2** | **18** | **4** | **22** |
|  | Unspecified (mild) | Mild AEs (no further details) | 1 | 1 | 7 | 4 | 11 |
|  | Epilepsy | New-onset epilepsy | 1 | 1 | 8 | 0 | 8 |
|  | Pain |  | 1 | 1 | 3 | 0 | 3 |
| **Non-invasive brain stimulation** | | | **17** | **88** | **66 *** | **37 *** | **203 *** |
|  | Headache | Headache, migraine | 7 | 17 | 6 * | 2 * | 41 * |
|  | Dizziness or headache |  | 6 | 12 | 1 * | 0 * | 34 * |
|  | Pain | Aching/burning, discomfort, neck pain, pain/burning/headache | 4 | 6 | 8 * | 13 * | 27 * |
|  | Local skin reaction | Erythema, erythema and blistering | 3 | 5 | 15 * | 3 * | 21 |
|  | Unspecified (mild) | Mild AEs (no further details) | 2 | 2 | 0 * | 0 * | 10 * |
|  | Abnormal sensation | Prickling, pruritus/tingling/burning, tingling | 4 | 6 | 5 * | 1 * | 9 * |
|  | Dizziness |  | 6 | 8 | 2 * | 2 * | 6 * |
|  | Abdominal pain | Abdominal pain, abdominal discomfort | 2 | 3 | 4 | 2 | 6 |
|  | Unspecified |  | 1 | 2 | 3 | 3 | 6 |
|  | Headache or skin reaction | Headache or erythema | 1 | 1 | 4 | 2 | 6 |
|  | Unspecified (moderate - severe) | Moderate - severe AEs (no further details) | 1 | 1 | 3 | 3 | 6 |
|  | Sleep disorder | Insomnia, sleep disorder | 4 | 3 | 2 * | 1 * | 5 |
|  | Scalp pain | Scalp discomfort | 2 | 1 | 1 * | 0 * | 5 |
|  | Dizziness and headache |  | 3 | 16 | 4 * | 0 * | 4 * |
|  | Nausea and vomiting | Nausea and vomiting, retching | 2 | 1 | 0 * | 1 * | 4 |
|  | Fatigue | Fatigue, sleepiness | 5 | 5 | 1 * | 2 * | 3 * |
|  | Nausea |  | 1 | 2 | 2 | 1 | 3 |
|  | Sneezing |  | 6 | 5 | 1 * | 0 * | 2 * |
|  | Anxiety |  | 5 | 5 | 2 * | 0 * | 2 * |
|  | Inattention |  | 1 | 1 | 1 | 0 | 1 |
|  | Palpitations |  | 1 | 1 | 1 | 0 | 1 |
|  | Visual change | Phosphene | 1 | 1 | 0 | 1 | 1 |
|  | Dizziness, pain, abnormal sensation, sneezing | Transient dizziness, pain, pins and needles, sneezing | 1 | 6 | NR | NR | NR |
|  | Sleep disorder and nausea | Insomnia and nausea | 1 | 1 | NR | NR | NR |
|  | Inattention or sleep disorder |  | 1 | 1 | NR | NR | NR |
|  | Seizure |  | 1 | 1 | NR | NR | NR |
| **Non-invasive brain stimulation + Cognitive rehabilitation** | | | **1** | **3** | **2 *** | **0 *** | **2 *** |
|  | Dizziness |  | 1 | 2 | NR | NR | NR |
|  | Headache |  | 1 | 2 | NR | NR | NR |
|  | Scalp pain |  | 1 | 1 | 2 | 0 | 2 |
| **Non-invasive brain stimulation, Acupuncture ^d^** | | | **1** | **NR** | **NR** | **NR** | **117** |
|  | Dizziness and headache |  | 1 | NR | NR | NR | 33 |
|  | Nausea and vomiting |  | 1 | NR | NR | NR | 22 |
|  | Personal reasons |  | 1 | NR | NR | NR | 18 |
|  | Diarrhoea |  | 1 | NR | NR | NR | 11 |
|  | Abdominal pain | Abdominal discomfort | 1 | NR | NR | NR | 6 |
|  | Local skin reaction | Erythema | 1 | NR | NR | NR | 5 |
|  | Change in condition |  | 1 | NR | NR | NR | 4 |
|  | Sleep disorder | Insomnia | 1 | NR | NR | NR | 4 |
|  | Fever |  | 1 | NR | NR | NR | 4 |
|  | Respiratory infection |  | 1 | NR | NR | NR | 2 |
|  | Epilepsy |  | 1 | NR | NR | NR | 2 |
|  | Ischaemic stroke |  | 1 | NR | NR | NR | 1 |
|  | Chronic hydrocephalus |  | 1 | NR | NR | NR | 1 |
|  | Dizziness |  | 1 | NR | NR | NR | 1 |
|  | Inattention | Lack of concentration | 1 | NR | NR | NR | 1 |
|  | Subdural haematoma following fall |  | 1 | NR | NR | NR | 1 |
|  | Haemorrhagic transformation |  | 1 | NR | NR | NR | 1 |
| **SSRI** | | | **1** | **NR** | **NR** | **NR** | **NR** |
|  | Gastrointestinal AEs |  | 1 | 20 | NR | NR | NR |
|  | Bleeding |  | 1 | 10 | NR | NR | NR |
|  | Seizure |  | 1 | 8 | NR | NR | NR |
| **Traditional Chinese medicine** | | | **1** | **12** | **NR** | **NR** | **NR** |
|  | Gastrointestinal AEs | Including nausea, vomiting, constipation, stomach discomfort, loss of appetite | 1 | 12 | NR | NR | NR |
|  | Nervous system AEs | Including drowsiness, insomnia, dizziness, fatigue | 1 | 9 | NR | NR | NR |
|  | Unspecified | Other AEs including dry mouth, frequent urination, rash, BP fluctuations | 1 | 8 | NR | NR | NR |
| **Virtual reality** | | | **2** | **2** | **0 *** | **1 *** | **1 *** |
|  | Unspecified |  | 1 | 1 | 0 | 1 | 1 |
|  | Dizziness |  | 1 | 1 | NR |  | NR |

AE = adverse event; SR = systematic review; RCT = randomised controlled trial; I = intervention group; C = control group; NR = not reported.

^a^ Details before further grouping, as reported in SRs.

^b^ Number of RCTs and events not adjusted for paper overlap.

^c^ Total may not match intervention + control group totals as some SRs did not report number of events by group.

^d^ Unable to separate intervention categories in one SR for this outcome as not reported at RCT or intervention category level in SR. Also note that this SR reported AEs and withdrawals together.

^*^ Total likely understated as some SRs only reported number of RCTs and did not comment on number of events at this level.

**Supplementary Table 5. Details of withdrawals reported by included systematic reviews.**

| **Systematic review paper** | **Intervention categories** | **No of primary studies (all outcomes)** | | | | **No of participants in RCTs that reported on withdrawals** | | | **Dropouts/withdrawals (no of events / no of RCTs)** | | **Meta-analysis** | **Additional information** |
| --- | --- | --- | --- | --- | --- | --- | --- | --- | --- | --- | --- | --- |
|  |  | **Total** | **Reporting on withdrawals** | **No withdrawals** | **Withdrawals** | **I** | **C** | **Total** | **I** | **C** |  |  |
| Elsner 2020 | Non-invasive brain stimulation | 67 | 48 | NR | NR | NR | NR | NR | Dropouts (16 / 8 RCTs) due to: unspecified reasons (9), organisational issues (2), medical problems (2), pneumonia (1), refusal (1), skin reaction (1); Dropouts in follow-up (10 / 3 RCTs) due to: unspecified (4), unavailable (3), death from MI (2), refusal (1) | Dropouts (10 / 8 RCTs) due to: unspecified (4), knee pain and contractures (1), emergency hospital transfer (1), refusal (1), early discharge (1), robot failure (1), worsening of hand function (1); Dropouts during follow-up (5 / 2 RCT) due to: unavailable (3), refusal (1), unspecified (1) | "Dropouts, adverse events and deaths during intervention period": Intervention vs passive control: I: 45 events, C: 20 events. RR = 1.25 (0.74, 2.13), I^2^ = 1%. Intervention vs active control: I: 5 events, C: 2 events. RR = 1.76 (0.43, 7.17), I^2^ = 0%. | Dropout details differ between tabular data and forest plot/meta-analysis (perhaps because some studies did not also report on AEs which were included in the meta-analysis outcome). Details extracted from the tabular data. |
|  |  |  |  |  |  |  |  |  | Dropouts (11 / 3 RCTs) due to: frustration/tiredness during assessments (7), dizziness (1), headache (1), unspecified (2) | |  |  |
| Gibson 2022 | Other rehabilitation therapies | 24 | NR | NR | 1 | NR | NR | NR | Refusal (1 / 1 RCT); Withdrawal (2 / 1 RCT); Loss to follow-up (3 / 1 RCT) | |  | Loss to follow-up mentioned for further 3 RCTs but no further data or figures provided. 1 RCT reported no loss to follow-up. Unclear whether all RCTs reported withdrawal data. |
| Guan 2021 | Ginkgo ketone ester (EGB50) | 16 | 2 | 0 | 2 | 204 | 190 | 394 | Withdrawals (8 / 1 RCT) due to: health reasons (8); Excluded (8 / 1 RCT) due to: invalid samples (8); Loss to follow-up (10 / 1 RCT) | |  |  |
| Han 2023 | Non-invasive brain stimulation | 17 (excluding PCTs) | 17 | 12 | 5 | 381 | 385 | 766 | Dropouts (9 / 5 RCTs) | Dropouts (3 / 2 RCTs) |  | No information on reasons for dropouts reported in SR. |
| Han 2024 | Acupuncture | 39 | 39 | 28 | 11 | 1354 | 1349 | 2703 | 11 RCTs | |  | Number of withdrawals and reasons not reported in SR. |
| Hofmeijer 2023 | Non-invasive brain stimulation | 57 | 41 | 11 | 30 | 1005 | 860 | 1865 | Withdrawals (15 / 3 RCTs) due to: unspecified (11), commuting difficulties (3), surgery (1); Lost to follow-up (19 / 7 RCTs) due to: unspecified (17), unavailability (1), early termination (1) | Lost to follow-up (15 / 5 RCTs) due to: unspecified (15) |  | Details not reported for further 9 RCTs (not included in figures) that were described as reporting withdrawals/loss to follow-up. Reasons not reported for all RCTs. |
|  |  |  |  |  |  |  |  |  | Withdrawals before starting treatment (4 / 1 RCT); Withdrawals (60 / 7 RCTs) due to: unspecified (51), low motivation (4), respiratory tract infection (2), personal reasons (2), hospital transfer (1); Dropouts (10 / 2 RCTs); Lost to follow-up (57 / 11 RCTs) due to: unspecified (53), family reasons/loss of interest/time conflict (4); Excluded from analysis (3 / 1 RCT) | |  |  |
| Liu 2023 (2) | Acupuncture | 38 | 8 | 0 | 8 | 290 | 291 | 581 | Dropouts (33 / 8 RCTs) | Dropouts (32 / 8 RCTs) |  | No information on reasons for withdrawals reported in SR, though does state RCTs gave reasons. |
| Liu 2023 (3) | Acupuncture, Acupuncture + Cognitive rehabilitation | 62 | 62 | 59 | 3 | 2593 | 2480 | 5073 | Dropouts (9 / 3 RCTs) | Dropouts (10 / 3 RCTs) |  | , No information on reasons for withdrawals reported in SR, though does state RCTs gave reasons. |
|  | Acupuncture | 30 | 30 | 29 | 1 | 1385 | 1264 | 2649 | Dropouts (1 / 1 RCT) | Dropouts (2 / 1 RCT) |  |  |
|  | Acupuncture + Cognitive rehabilitation | 38 | 38 | 36 | 2 | 1560 | 1445 | 3005 | Dropouts (8 / 2 RCTs) | Dropouts (8 / 2 RCTs) |  |  |
| Li 2023 (3) | Exercise | 12 | 11 | 3 | 8 | 919 | 937 | 1856 | Dropout rate 0-33.5% (11 RCTs) | |  | No information on reasons for withdrawals reported in SR |
| Li 2024 | Exercise | 15 | 13 | 3 | 10 | 482 | 547 | 1029 | Dropouts (97 / 10 RCTs) | Dropouts (55 / 8 RCTs) |  | No information on reasons for withdrawals reported in SR. |
| Luo 2024 | Acupuncture | 29 | 13 | 4 | 9 | 593 | 588 | 1181 | 22 | 18 |  | No information on reasons for withdrawals reported in SR. |
| Ma 2021 | Virtual reality | 25 | 25 | 20 | 5 | 560 | 556 | 1116 | Withdrawal (3 / 2 RCTs); Death (1 / 1 RCT); Loss to follow-up (3 /2 RCTs) | Withdrawal (2 / 2 RCTs); Refusal (1 / 1 RCT); Loss to follow-up (2 / 2 RCTs) |  | No information on reasons for withdrawals reported in SR. |
| Toh 2023 | Non-invasive ventilation | 24 | 13 | 0 | 13 | 368 | 395 | 763 | Withdrawals (72 / 13 RCTs) | Withdrawals (20 / 7 RCTs) |  | Reasons for withdrawals not reported in SR. |
| Xu 2023 | Non-invasive brain stimulation, Acupuncture | 90 | NR | NR | NR | NR | NR | NR | AE leading to treatment discontinuation (30) including "personal reasons" (18) | |  | Unable to extract withdrawal data by intervention category. Note "personal reasons" is included for 18/30 subjects in list of AEs leading to treatment discontinuation. Unclear whether all RCTs reported withdrawal data. |
|  | Acupuncture | 12 | NR | NR | NR | NR | NR | NR | Unclear | Unclear |  |  |
|  | Non-invasive brain stimulation | 78 | NR | NR | NR | NR | NR | NR | Unclear | Unclear |  |  |
| Yang 2024 (1) | High-pressure oxygen, Exercise, Cognitive rehabilitation, Non-invasive brain stimulation, Moxibustion, Acupuncture, Remote ischemic conditioning, Intensive patient care program | 22 | 4 | NR | NR | NR | NR | NR | 4 RCTs | |  | Number of withdrawals and reasons not reported in SR. |
| Yang 2023 | Non-invasive ventilation | 7 | 7 | 3 | 4 | 98 | 89 | 187 | Dropouts (12 / 3 RCTs) | Dropouts (12 / 4 RCTs) |  | Dropout rate varied from 4.35 - 35% from studies that had dropouts. No information on reasons for dropouts reported in SR. |
| Yan 2024 | Non-invasive brain stimulation | 26 | 12 | 3 | 9 | 226 | 180 | 406 | Dropouts in baseline (1 / 1 RCT); Dropouts in study (3 / 2 RCTs); Dropouts in follow-up (7 / 4 RCTs); "Some" in follow-up (1 RCT) | Dropouts in baseline (1 / 1 RCT); Dropouts in study (1 / 1 RCT); Dropouts in follow-up (7 / 4 RCTs) |  | No information on reasons for withdrawals reported in SR. |
|  |  |  |  |  |  |  |  |  | Dropouts in follow-up (17 / 3 RCTs) | |  |  |

SR = systematic review; RCT = randomised controlled trial; I = intervention group; C = control group; NR = not reported; AE = adverse event.

**Supplementary Figure 1. Summary of risk of bias of RCTs as reported by systematic reviews using the Cochrane Collaboration’s Tool for Assessing Risk of Bias (RoB 1).**

* Not reported by all SRs.

**Supplementary Table 6. Summary of risk of bias analyses for RCTs as reported by systematic reviews.**

| **SR Ref** | **SRs' own assessment of Risk of Bias in RCTs included in global cognition outcome*** | | | | | | |
| --- | --- | --- | --- | --- | --- | --- | --- |
|  | **Cochrane RoB 1** | **Cochrane RoB 2** | **PEDro** | **Modified Jadad scale scoring system** | **Review instrument of Australia’s Joanna Briggs Institute (JBI) (Institute(JBI) TJB** | **GRADE** | **Publication bias** |
| Chen 2022 (1) |  |  | Totals: Askin - 7/10 (0 for concealed allocation, blinding subjects, blinding therapists), Fregni 9/10 (0 for concealed allocation), Liu 9/10 (0 for blinding therapists). Mean total (global cognition) = 8.33. |  |  | Moderate certainty |  |
| Chen 2022 (2) | Random sequence generation: 13/19 low, 6/19 unclear Allocation concealment: 17/19 low, 2/19 unclear Blinding of participants/personnel: 2/19 low, 17/19 unclear Blinding of outcome assessment: 4/19 low, 15/19 unclear Incomplete outcome data: 19/19 low Selective reporting bias: 19/19 low Other bias: 19/19 low |  |  |  |  |  | Funnel plot - publication bias unlikely. |
| Chen 2024 | Random sequence generation: 35/61 low, 25/61 unclear, 1/61 high Allocation concealment: 3/61 low, 58/61 unclear Blinding of participants/personnel: 16/61 low, 45/61 unclear Blinding of outcome assessment: 21/61 low, 40/61 unclear Incomplete outcome data: 60/61 low, 1/61 high Selective reporting bias: 60/61 low, 1/61 high Other bias: 58/61 low, 3/61 high |  |  |  |  |  |  |
| Daoud 2024 | Overall: 1 low, 4 unclear, 1 high Random sequence generation: 6/6 low Allocation concealment: 2/6 low, 4/6 unclear Blinding of participants/personnel: 3/6 low, 3/6 unclear Blinding of outcome assessment: 2/6 low, 3/6 unclear, 1/6 high Incomplete outcome data: 6/6 low Selective reporting bias: 4/6 low, 2/6 unclear Other bias: 2/6 low, 4/6 unclear |  |  |  |  | Low certainty | Not conducted due to limited number of studies. |
| Elsner 2020 | Random sequence generation: 2/2 unclear Allocation concealment: 2/2 unclear Blinding of participants/personnel: 1/2 low, 1/2 unclear Blinding of outcome assessment: 1/2 low, 1/2 unclear Incomplete outcome data: 1/2 unclear, 1/2 high Selective reporting bias: 2/2 unclear Other bias: |  |  |  |  | Low certainty | Funnel plot - publication bias suspected. |
| Fan 2022 |  | Overall: 9/26 low, 4/26 moderate, 13/26 high Randomisation process: 19/26 low, 6/26 some concerns, 1/26 high Deviations from the intended interventions: 24/26 low, 2/26 some concerns Missing outcome data: 24/26 low, 2/26 high Measurement of the outcome: 16 low, 10/26 high Selection of the reported result: 26/26 low |  |  |  | Very low certainty for MoCA outcome for NBP monotherapy and MMSE outcome for NBP combination therapy. Low certainty for MMSE outcome for NBP monotherapy and MoCA outcome for NBP combination therapy. | Funnel plot - publication bias strongly suspected. |
| Gao 2021 | Random sequence generation: 4/4 low Allocation concealment: 4/4 low Blinding of participants/personnel: 4/4 unclear Blinding of outcome assessment: 4/4 low Incomplete outcome data: 3/4 low, 1/4 high Selective reporting bias: 4/4 low Other bias: 4/4 low |  | Only included studies that scored >=6/10 total. Only overall scores provided (6, 7, 7, 9). Mean total (global cognition) = 7.25. |  |  |  |  |
| Gao 2023 |  | Overall: 1/6 some concerns, 5/6 high Randomisation process: 3/6 low, 3/6 some concerns Deviations from the intended interventions: 1/6 low, 5/6 high Missing outcome data: 2/6 low, 1/6 some concerns, 3/6 high Measurement of the outcome: 4/6 low, 2/6 high Selection of the reported result: 1/6 low, 5/6 some concerns | "Studies with PEDro Scale scores 6–10 points were considered good quality, 5 points or lower represented low quality." Excluded RCTs that scored <6/10 points total from combined analysis. Only overall scores provided (6, 7, 8, 8, 8, 9). Mean total (global cognition) = 7.67. |  |  | Low certainty |  |
| Gibson 2022 | Random sequence generation: 8/11 low, 3/11 unclear Allocation concealment: 1/11 low, 10/11 unclear Blinding of participants/personnel: 11/11 high Blinding of outcome assessment: 4/11 low, 6/11 unclear, 1/11 high Incomplete outcome data: 4/11 low, 6/11 unclear, 1/11 high Selective reporting bias: 10/11 low, 1/11 high Other bias: 11/11 low |  |  |  |  | Low certainty. Note GRADE does not include LOTCA. | States used funnel charts to assess publication bias but figures not included for global cognition outcome. |
| Gong 2023 | Random sequence generation: 10/10 low Allocation concealment: 8/10 low, 2/10 unclear Blinding of participants/personnel: 9/10 low, 1/10 unclear Blinding of outcome assessment: 9/10 low, 1/10 high Incomplete outcome data: 10/10 low Selective reporting bias: 10/10 low Other bias: 7/10 low, 3/10 unclear |  |  |  |  | Moderate certainty for MMSE, MoCA and LOTCA outcomes | No concerns from GRADE table but not commented on. |
| Guan 2021 | Only presented in graph format and unable to separate RCTs included in global cognition outcome from others. Narratively ~75% low/25% unclear RoB for random sequence generation; <25% low/>75% high RoB for allocation concealment; 100% high RoB for blinding of participants and personnel, and outcome assessment; <25% low/>75% unclear RoB for incomplete outcome data; 100% low RoB for selective reporting. |  |  |  |  |  | Funnel plot - asymmetry suggesting possible publication bias, and that long-term efficacy outcomes may have been underestimated. |
| Gunawan 2024 | States RoB 2 in methods but tool used appears to be RoB 1. Overall: 3/6 low, 2/6 unclear, 1/6 high Random sequence generation: 6/6 low Allocation concealment: 6/6 low Blinding of participants/personnel: 6/6 high Blinding of outcome assessment: 3/6 low, 2/6 low, 1/6 high Incomplete outcome data: 6/6 low Selective reporting bias: 6/6 low Other bias: 6/6 low |  |  |  |  |  |  |
| Han 2023 | Random sequence generation: 16/16 low Allocation concealment: 2/16 low, 14/16 unclear Blinding of participants/personnel: 11/16 low, 5/16 unclear Blinding of outcome assessment: 11/16 low, 5/16 unclear Incomplete outcome data: 15/16 low, 1/16 unclear Selective reporting bias: 16/16 low Other bias: 15/16 low, 1/16 unclear  Note RoB assessment includes PCTs, data for which was not otherwise extracted as part of this umbrella review. |  | "studies with a score of <6 were considered low-quality". Only overall scores provided (7, 7, 8, 8, 8, 9, 9, 9, 9, 9, 9, 9, 9, 10, 10, 10). Mean total (global cognition excluding PCTs) = 8.75.  Note RoB assessment includes PCTs, data for which was not otherwise extracted as part of this umbrella review. |  |  | Moderate certainty for MMSE and MoCA outcomes. Note GRADE assessment includes PCTs, data for which was not otherwise extracted as part of this umbrella review. |  |
| Han 2024 | Random sequence generation: 29/39 low, 10/39 unclear Allocation concealment: 12/39 low, 27/39 unclear Blinding of participants/personnel: 6/39 low, 31/39 unclear, 2/39 high Blinding of outcome assessment: 8/39 low, 29/39 unclear, 2/39 high Incomplete outcome data: 35/39 low, 4/39 high Selective reporting bias: 34/39 low, 5/39 high Other bias: 39/39 unclear |  |  |  |  |  | Funnel plot - "excellent symmetry" and no significant publication bias for MMSE and non-global cognition outcomes reported (MoCA not mentioned). |
| Hofmeijer 2023 |  | Overall: 5/8 low, 2/8 some concerns, 1/8 high. Tool used appears to be mix of RoB 1 and RoB 2. States RoB 2 but RoB 1 domains used with some signalling questions taken from RoB 2. No score by domain given, only overall. |  |  |  |  | Funnel plots showed symmetrical distribution suggesting no evidence for publication bias. |
| Kalbouneh 2022 |  | Overall: 4/4 low Randomisation process: 4/4 low Deviations from intended intervention: 4/4 low Missing outcome data: 4/4 low Measurement of the outcome: 4/4 low Selection of the reported result: 4/4 low |  |  |  |  |  |
| Khattab 2021 | Random sequence generation: 5/6 low, 1/6 NR Allocation concealment: 3/6 low, 3/6 NR Blinding of participants/personnel: 4/6 low, 2/6 NR Blinding of outcome assessment: 5/6 low, 1/6 NR Incomplete outcome data: 5/6 low, 1/6 high Selective reporting bias: 6/6 low |  |  |  |  | Low certainty. | Publication bias = "none" as part of GRADE but no funnel charts or data on this. |
| Kuang 2021 | Overall: 4/28 low, 24/28 high Random sequence generation: 16/28 low, 12/28 unclear Allocation concealment: 3/28 low, 25/28 unclear Blinding of participants/personnel: 28/28 high Blinding of outcome assessment: 5/28 low, 22/28 unclear, 1/28 high Incomplete outcome data: 27/28 low, 1/28 unclear Selective reporting bias: 28/28 unclear Other bias: 28/28 low |  |  |  |  | Very low certainty for MoCA and MMSE outcomes. | Funnel plot/Egger's - no obvious publication bias for MoCA (not assessed for other outcomes). |
| Li 2023 (1) | Overall: 5/8 low, 1/8 unclear, 2/8 high Random sequence generation: 8/8 low Allocation concealment: 1/8 low, 7/8 unclear Blinding of participants/personnel: 8/8 low Blinding of outcome assessment: 8/8 low Incomplete outcome data: 5/8 low, 3/8 high Selective reporting bias: 8/8 low Other bias: 8/8 unclear |  |  |  |  |  | Funnel plot - no significant publication bias. |
| Li 2022 (1) | Random sequence generation: 12/16 low, 4/16 unclear Allocation concealment: 5/16 low, 11/16 unclear Blinding of participants/personnel: 4/16 low, 12/16 unclear Blinding of outcome assessment: 4/16 low, 12/16 unclear Incomplete outcome data: 16/16 low Selective reporting bias: 16/16 low Other bias: 6/16 low, 9/16 unclear, 1/16 high |  |  |  |  |  | Funnel plot - may be degree of publication bias toward right side with 3 studies outside the 95% CI suggesting small-sample effect. |
| Li 2021 | Random sequence generation: 6/13 low, 7/13 unclear Allocation concealment: 13/13 unclear Blinding of participants/personnel: 12/13 low, 1/13 unclear Blinding of outcome assessment: 13/13 low Incomplete outcome data: 13/13 low Selective reporting bias: 12/13 low, 1/13 unclear Other bias: 3/13 low, 10/13 unclear |  |  |  |  | Very low certainty for MMSE outcome for Acupuncture + TMS vs Conventional treatment (Direct), Acupuncture + HBO vs Conventional treatment (Direct), Acupuncture + HBO vs Acupuncture (Direct), Acupuncture + TMS vs HBO (Indirect), Acupuncture + UWE vs HBO (Indirect), Acupuncture + RehaCom vs HBO (Indirect). Low certainty for MMSE outcome for Acupuncture + UWE vs Conventional treatment (Indirect), Acupuncture + RehaCom vs Conventional treatment (Direct), Acupuncture + RehaCom vs Acupuncture (Direct). Moderate certainty for MMSE outcome for Acupuncture + TMS vs Acupuncture (Direct), Acupuncture + UWE vs Acupuncture (Direct), Acupuncture + HBO vs HBO (Direct). No GRADE assessment for MoCA outcome. The above only reflects GRADE assessments for data extracted and included in this umbrella review. | Funnel charts - mostly symmetrical but may still be degree of publication bias. No bias analysis performed for direct comparisons as not enough data. |
| Li 2023 (2) | Random sequence generation: 24/29 low, 4/29 unclear, 1/29 high Allocation concealment: 29/29 unclear Blinding of participants/personnel: 1/29 low, 28/29 unclear Blinding of outcome assessment: 1/29 low, 28/29 unclear Incomplete outcome data: 29/29 low Selective reporting bias: 29/29 low Other bias: 29/29 unclear |  |  |  |  |  | Funnel plot for MMSE showed small possibility of publication bias and small sample effects. |
| Lin 2022 | Random sequence generation: 19/22 low, 2/22 NR, 1/22 high Allocation concealment: 14/22 low, 8/22 NR Blinding of participants/personnel: 4/22 low, 15/22 NR, 3/22 high Blinding of outcome assessment: 13/22 low, 7/22 NR, 2/22 high Incomplete outcome data: 22/22 low Selective reporting bias: 22/22 low Other bias: 22/22 NR |  |  |  |  |  | Funnel plot/Egger's reported as no publication bias. |
| Liu 2023 (1) | Random sequence generation: 14/17 low, 3/17 unclear Allocation concealment: 17/17 unclear Blinding of participants/personnel: 17/17 unclear Blinding of outcome assessment: 1/17 low, 16/17 unclear Incomplete outcome data: 17/17 low Selective reporting bias: 17/17 low Other bias: 17/17 low |  |  |  |  |  |  |
| Liu 2021 | Random sequence generation: 3/4 low, 1/4 unclear Allocation concealment: 4/4 unclear Blinding of participants/personnel: 4/4 low Blinding of outcome assessment: 3/4 low, 1/4 unclear Incomplete outcome data: 2/4 low, 2/4 high Selective reporting bias: 4/4 low Other bias: 4/4 low |  |  |  |  | Moderate certainty for MMSE and LOTCA outcomes. | "Due to the limited number of studies included for each result, there may be publication biases." |
| Liu 2020 |  | Overall: 1/19 low, 14/19 some concerns, 4/19 high Randomisation process: 3/19 low, 16/19 some concerns Deviations from intended intervention: 19/19 low Missing outcome data: 14/19 low, 5/19 high Measurement of the outcome: 3/19 low, 16/19 some concerns Selection of the reported result: 1/19 low, 18/19 some concerns |  |  |  |  | Funnel plot/Begg's/Egger's assessed for MMSE - no significant publication bias. Couldn't be assessed for MoCA or other outcomes due to small number of studies. |
| Liu 2024 (1) | Random sequence generation: 57/72 low, 13/72 unclear, 2/72 high Allocation concealment: 5/72 low, 66/72 unclear, 1/72 high Blinding of participants/personnel: 30/72 low, 40/72 unclear, 2/72 high Blinding of outcome assessment: 25/72 low, 46/72 unclear, 1/72 high Incomplete outcome data: 69/72 low, 3/72 high Selective reporting bias: 70/72 low, 2/72 unclear Other bias: 67/72 low, 5/72 unclear |  |  |  |  |  | Funnel plots - "potential" publication bias. |
| Liu 2024 (2) | Overall as too difficult to extract for global cognition papers only: Random sequence generation: 41/53 low, 11/53 unclear, 1/53 high Allocation concealment: 30/53 low, 23/53 unclear Blinding of participants/personnel: 11/53 low, 25/53 unclear, 17/53 high Blinding of outcome assessment: 33/53 low, 16/53 unclear, 4/53 high Incomplete outcome data: 51/53 low, 2/53 high Selective reporting bias: 51/53 low, 2/53 high |  |  |  |  |  | "Funnel plot analyses did not provide strong evidences of asymmetry across all analyses." |
| Liu 2023 (2) | Random sequence generation: 28/38 low, 10/38 unclear Allocation concealment: 38/38 unclear Blinding of participants/personnel: 5/38 low, 33/38 high Blinding of outcome assessment: 7/38 low, 31/38 high Incomplete outcome data: 30/38 low, 8/38 high Selective reporting bias: 12/38 low, 26 unclear Other bias: 38/38 low |  |  |  |  | Low certainty for MMSE, MoCA and LOTCA outcomes. | Funnel plots/Egger's indicated possible publication bias. |
| Liu 2023 (3) |  | Overall: 2/62 low, 57/62 unclear, 3/62 high Randomisation process: 41/62 low, 21/62 unclear Deviations from the intended intervention: 6/62 low, 56/62 unclear Missing outcome data: 57/62 low, 2/62 unclear, 3/62 high Measurement of the outcome: 9/62 low, 53/62 unclear Selection of the reported result: 12/62 low, 50/62 unclear |  |  |  | Very low certainty for MMSE outcome for Body acupuncture vs Cognitive training, Electro-acupuncture + Cognitive training vs Cognitive training, Abdominal acupuncture + Body acupuncture vs Cognitive training. Very low certainty for MoCA outcome for Scalp acupuncture vs Cognitive training, Warm acupuncture + Scalp acupuncture vs Cognitive training. Low certainty for MMSE outcome for SA + BA vs Cognitive training, Scalp acupuncture + Cognitive training vs Cognitive training, Body acupuncture + Cognitive training vs Cognitive training, Ophthalmic acupuncture + Cognitive training vs Cognitive training, Warm acupuncture + Cognitive training vs Cognitive training, Auricular bloodletting + Cognitive training vs Cognitive training, Scalp acupuncture + Auricular bloodletting vs Cognitive training, Scalp acupuncture vs Cognitive training. Low certainty for MoCA outcome for Scalp acupuncture + Cognitive training vs Cognitive training, Warm acupuncture + Cognitive training vs Cognitive training, Electro-acupuncture + Cognitive training vs Cognitive training, Abdominal acupuncture + Cognitive training vs Cognitive training. Moderate certainty for MoCA outcome for Scalp acupuncture + Body acupuncture vs Cognitive training. GRADE assessments not reported for MMSE outcome for Abdominal acupuncture + Cognitive training vs Cognitive training, Body acupuncture + Cognitive training vs Cognitive training, Body acupuncture vs Cognitive training (likely due to being indirect data only). | Funnel plots: MoCA indicated "moderate sample size" with "low likelihood of publication bias". MMSE was "more dispersed" indicating "possible publication bias". |
| Li 2023 (3) | Random sequence generation: 3/5 low, 2/5 unclear Allocation concealment: 2/5 low, 2/5 unclear, 1/5 high Blinding of participants/personnel: 1/5 low, 4/5 unclear Blinding of outcome assessment: 3/5 low, 2/5 unclear Incomplete outcome data: 4/5 low, 1/5 high Selective reporting bias: 4/5 low, 1/5 unclear Methods advised blinding of participants and personnel domain would not be assessed but has been included in results/figures. |  |  |  |  |  |  |
| Li 2022 (2) | Random sequence generation: 5/6 low, 1/6 unclear Allocation concealment: 5/6 low, 1/6 unclear Blinding of participants/personnel: 2/6 low, 2/6 unclear, 2/6 high Blinding of outcome assessment: 3/6 low, 2/6 unclear, 1/6 high Incomplete outcome data: 4/6 low, 1/6 unclear, 1/6 high Selective reporting bias: 6/6 low Other bias: 6/6 low |  |  |  |  |  |  |
| Li 2024 | Random sequence generation: 11/13 low, 2/13 unclear Allocation concealment: 10/13 low, 1/13 unclear, 2/13 high Blinding of participants/personnel: 6/13 low, 4/13 unclear, 3/13 high Blinding of outcome assessment: 4/13 low, 3/13 unclear, 6/13 high Incomplete outcome data: 9/13 low, 3/13 unclear, 1/13 high Selective reporting bias: 11/13 low, 2/13 unclear Other bias: 8/13 low, 5/13 unclear |  | Note totals presented in table differ from text and all overestimated by 1 (apart from for Koch 2020) as authors included item 1 (eligibility criteria) when totalling so /11 instead of /10. Totals as reported: Deijle 9/11 (0 for blinding participants, blinding therapists); Fang 8/11 (0 for blinding participants, blinding therapists, blinding assessors); Hsu 10/11 (0 for blinding therapists); Ihle-Hansen 8/11 (0 for allocation concealment, blinding therapists, blinding assessors); Koch 6/10 (0 for allocation concealment, blinding participants, blinding therapists, blinding assessors); Lapointe 7/11 (0 for allocation concealment, blinding participants, blinding therapists, blinding assessors); Yeh 9/11 (0 for blinding participants, blinding therapists). Mean total (as reported) = 8.14. Mean total (corrected) = 7.29. |  |  |  |  |
| Li 2022 (3) | Random sequence generation: 38/55 low, 17/55 unclear Allocation concealment: 9/55 low, 46/55 unclear Blinding of participants/personnel: 2/55 low, 31/55 unclear, 22/55 high Blinding of outcome assessment: 12/55 low, 43/55 unclear Incomplete outcome data: 55/55 low Selective reporting bias: 5/55 low, 50/55 unclear Other bias: 11/55 low, 44/55 unclear |  |  |  |  | Very low certainty for MMSE outcome for VR vs Normal rehabilitation. Low certainty for MoCA outcome for TMS vs Normal rehabilitation, tDCS vs Normal rehabilitation, VR vs Normal rehabilitation, Computer-assisted cognitive rehabilitation vs Normal rehabilitation, Acupuncture vs Normal rehabilitation. Low certainty for MMSE outcome for TMS vs Normal rehabilitation, tDCS vs Normal rehabilitation, Computer-assisted cognitive rehabilitation vs Normal rehabilitation, Acupuncture vs Normal rehabilitation. | Funnel plots/Egger's - MMSE - "little publication bias likely". MoCA - "suggested a publication bias". |
| Luo 2024 | Random sequence generation: 23/26 low, 3/26 unclear Allocation concealment: 9/26 low, 17/26 unclear Blinding of participants/personnel: 6/26 low, 20/26 unclear Blinding of outcome assessment: 11/26 low, 15/26 unclear Incomplete outcome data: 23/26 low, 3/26 high Selective reporting bias: 26/26 low Other bias: 26/26 unclear Overall: 23/26 unclear, 3/26 high RoB 2 analysis presented in main figures but supplementary material contains RoB 1 analysis which is also described in the text. RoB 1 analysis for 1 paper is missing (paper also missing from study characteristics table). | Overall: 13/27 low, 12/27 unclear, 2/27 high Randomisation process: 19/27 low, 8/27 unclear Deviations from intended intervention: 24/27 low, 2/27 unclear, 1/27 high Missing outcome data: 25/27 low, 1/27 unclear, 1/27 high Measurement of the outcome: 26/27 low, 1/27 unclear Selection of the reported result: 25/27 low, 2/27 unclear RoB 2 analysis presented in main figures but supplementary material contains RoB 1 analysis which is also described in the text. RoB 1 analysis for 1 paper is missing (paper also missing from study characteristics table). |  |  |  |  | Funnel plots/Egger's indicated no publication bias for MoCA, but that there was for MMSE. |
| Lyu 2023 | Random sequence generation: 12/18 low, 6/18 unclear Allocation concealment: 1/18 low, 17/18 unclear Blinding of participants/personnel: 2/18 low, 16/18 unclear Blinding of outcome assessment: 2/18 low, 16/18 unclear Incomplete outcome data: 18/18 low Selective reporting bias: 18/18 low Other bias: 17/18 low, 1/18 high |  |  |  |  |  |  |
| Ma 2021 | Provided information about RCT methodology where not "unclear" but did not assess as low/high. Domains also do not fully line up with RoB 1 tool. |  |  |  |  |  |  |
| Mingming 2022 |  |  |  |  | Overall: A=1 (high quality), B=5 (moderate quality) True randomisation: 1/6 yes, 5/6 unclear Allocation concealment: 1/6 yes, 5/6 unclear Similar baseline: 6/6 yes Participant blindness: 1/6 yes, 5/6 unclear Blindness of intervention implement: 1/6 yes, 4/6 unclear, 1/6 no Blindness of outcome evaluator: 3/6 yes, 3/6 unclear Treatment groups treated identically: 6/6 yes Complete follow-up or strategies to address incomplete follow-up: 6/6 yes Participants analysed in groups they were randomised: 6/6 yes Outcomes measured in same way: 6/6 yes Outcomes measured reliably: 6/6 yes Appropriate statistical analysis: 6/6 yes Proper trial: 6/6 yes |  |  |
| Nie 2021 | Random sequence generation: 8/18 low, 9/18 unclear, 1/18 high Allocation concealment: 1/18 low, 17/18 unclear Blinding of participants/personnel: 1/18 low, 17/18 unclear Blinding of outcome assessment: 1/18 low, 17/18 unclear Incomplete outcome data: 18/18 low Selective reporting bias: 17/18 low, 1/18 high Other bias: 16/18 low, 2/18 high |  |  |  |  |  |  |
| Lin 2024 | Random sequence generation: 11/12 low, 1/12 unclear Allocation concealment: 8/12 low, 4/12 unclear Blinding of participants/personnel: 1/12 low, 6/12 unclear, 5/12 high Blinding of outcome assessment: 5/12 low, 2/12 unclear, 5/12 high Incomplete outcome data: 9/12 low, 3/12 unclear Selective reporting bias: 12/12 low Other bias: 12/12 low |  |  |  |  | Low certainty | Funnel plot asymmetrical - possible publication bias. |
| Shen 2022 |  | Overall: 11/34 low, 7/34 unclear, 16/34 high Randomisation process: 21/34 low, 13/34 unclear Deviations from the intended intervention: 29/34 low, 5/34 unclear Missing outcome data: 30/34 low, 4/34 high Measurement of the outcome: 20/34 low, 14/34 high Selection of the reported result: 34/34 low |  |  |  | Very low certainty for MoCA and MMSE outcomes. | Funnel plot asymmetry - strongly suspected publication bias. |
| Toh 2023 | Overall: 8/8 high Random sequence generation: 7/8 low, 1/8 unclear Allocation concealment: 4/8 low, 4/8 unclear Blinding of participants/personnel: 8/8 high Blinding of outcome assessment: 4/8 low, 4/8 unclear Incomplete outcome data: 4/8 low, 1/8 unclear, 3/8 high Selective reporting bias: 3/8 low, 5/8 unclear Other bias: 7/8 low, 1/8 unclear |  |  |  |  | Very low certainty | Funnel plots/Egger's used for other outcomes indicating no publication bias but insufficient global cognition studies. |
| Valenzuela-Lopez 2024 |  | Overall: 6/11 unclear, 5/11 high Randomisation process: 5/11 low, 6/11 unclear Deviations from the intended intervention: 6/11 unclear, 5/11 high Missing outcome data: 11/11 low Measurement of the outcome: 10/11 low, 1/11 unclear Selection of the reported result: 7/11 low, 4/11 unclear | Totals: Adomaviciene 4/10 (0 for allocation concealment, blinding subjects, blinding therapists, blinding assessors, outcome measures, intention-to-treat); Li 5/10 (0 for allocation concealment, blinding subjects, blinding therapists, blinding assessors, intention-to-treat); Ranzani 5/10 (0 for allocation concealment, blinding subjects, blinding therapists, outcome measures, intention-to-treat); Shang 5/10 (0 for allocation concealment, blinding subjects, blinding therapists, blinding assessors, intention-to-treat); Taravati 5/10 (0 for allocation concealment, blinding subjects, blinding therapists, blinding assessors, intention-to-treat); Oh 6/10 (0 for blinding subjects, blinding therapists, blinding assessors, intention-to-treat); Kim 6/10 (0 for blinding subjects, blinding therapists, blinding assessors, intention-to-treat); Choi 6/10 (0 for allocation concealment, blinding subjects, blinding therapists, blinding assessors); Ozen 6/10 (0 for blinding subjects, blinding therapists, blinding assessors, intention-to-treat); Wilson 6/10 (0 for blinding subjects, blinding therapists, blinding assessors, intention-to-treat); Mao 7/10 (0 for blinding subjects, blinding therapists, blinding assessors). Mean total = 5.55. |  |  |  | D measure: Publication bias "considered negligible" |
| Wang 2022 (1) |  |  | "Trial quality ratings are as follows: 0–4 points for low quality, 4–5 points for general quality, 5–8 points for good quality, and 9–10 points for high quality." Item 1 (eligibility criteria) not reported. Totals: Gueye 6/10 (0 for allocation concealment, blinding subjects, blinding therapists, blinding assessors); Oh 7/10 (0 for blinding subjects, blinding therapists, blinding assessors); Rogers 7/10 (0 for blinding subjects, blinding therapists, blinding assessors). Mean total = 6.67. |  |  |  |  |
| Wang 2023 | Random sequence generation: 7/12 low, 5/12 unclear Allocation concealment: 5/12 low, 7/12 unclear Blinding of participants/personnel: 9/12 low, 3/12 high Blinding of outcome assessment: 10/12 low, 2/12 high Incomplete outcome data: 12/12 low Selective reporting bias: 12/12 low Other bias: 5/12 low, 7/12 unclear |  |  |  |  | Moderate certainty | Funnel plots/Egger's - no bias in global cognitive function, but is for other outcomes. |
| Wang 2022 (2) | Random sequence generation: 10/16 low, 6/16 unclear Allocation concealment: 1/16 low, 15/16 unclear Blinding of participants/personnel: 16/16 low Blinding of outcome assessment: 9/16 low, 7/16 unclear Incomplete outcome data: 16/16 low Selective reporting bias: 16/16 low Other bias: 16/16 low |  | "A score out of 10 was assigned, with 7 being high quality, 5-6 being moderate quality, and 4 being low quality." Totals: Yun 9/10 (0 for allocation concealment); Zeng 7/10 (0 for allocation concealment, blinding therapists, blinding assessors); Ai 8/10 (0 for allocation concealment, blinding therapists); Liu 2021 8/10 (0 for allocation concealment, blinding therapists); Chen 7/10 (0 for allocation concealment, blinding therapists, blinding assessors); Yan 7/10 (0 for allocation concealment, blinding therapists, blinding assessors); Ko 9/10 (0 for allocation concealment); Liu 2020 10/10; Yin 8/10 (0 for allocation concealment, blinding therapists); Ma 7/10 (0 for allocation concealment, blinding therapists, blinding assessors); Zhang and Zou 8/10 (0 for allocation concealment, blinding therapists); Li 2020 9/10 (0 for allocation concealment); Lu 8/10 (0 for allocation concealment, blinding assessors); Li 2021 7/10 (0 for allocation concealment, blinding therapists, blinding assessors); Zhang 7/10 (0 for allocation concealment, blinding therapists, blinding assessors); Li 2022 9/10 (0 for allocation concealment). Mean total = 8. |  |  |  | Funnel plots - "generally symmetrical". |
| Wang 2024 | Overall: 1/38 low, 27/38 moderate, 10/38 high Random sequence generation: 28/38 low, 8/38 unclear, 2/38 high Allocation concealment: 36/38 unclear, 2/38 high Blinding of participants/personnel: 2/38 low, 36/38 high Blinding of outcome assessment: 2/38 low, 36/38 high Incomplete outcome data: 38/38 low Selective reporting bias: 38/38 low Other bias: 34/38 low, 4/38 unclear |  |  |  |  |  | Funnel plot for MMSE showed slight asymmetry indicating possible publication bias. Not mentioned for MoCA. |
| Wang 2022 (3) | Random sequence generation: 14/14 low Allocation concealment: 14/14 unclear Blinding of participants/personnel: 14/14 unclear Blinding of outcome assessment: 14/14 unclear Incomplete outcome data: 14/14 low Selective reporting bias: 14/14 low Other bias: 6/14 low, 8/14 high |  |  |  |  |  | Funnel charts "roughly symmetrical" but figures not included in article. Egger/Begg's indicated "no potential publication bias". |
| Wiley 2022 | Random sequence generation: 1/5 NR, 4/5 low Allocation concealment: 1/5 NR, 4/5 low Blinding of participants/personnel: 1/5 NR, 3/5 unclear 1/5 high Blinding of outcome assessment: 1/5 NR, 1/5 low, 3/5 high  Incomplete outcome data: 5/5 low Selective reporting bias: 5/5 low Other bias: 5/5 low |  |  |  |  |  |  |
| Xiao 2024 | Random sequence generation: 5/5 low Allocation concealment: 1/5 low, 4/5 unclear Blinding of participants/personnel: 1/5 low, 4/5 unclear Blinding of outcome assessment: 1/5 low, 4/5 unclear Incomplete outcome data: 5/5 low Selective reporting bias: 5/5 low Other bias: 5/5 low |  |  | "Studies were scored on a scale from 0 to 7, with scores of 3 or lower indicating low quality and scores of 4 or higher indicating high quality." Low scores for randomisation and blinding. Overall: 2/5 low quality, 3/5 high quality. |  | Low certainty | Funnel plot but not for cognitive outcomes - "approximate symmetry, indicating minimal publication bias". "None found" for GRADE analysis. |
| Xiao 2022 | Random sequence generation: 21/21 low Allocation concealment: 8/21 low, 13/21 unclear Blinding of participants/personnel: 14/21 low, 6/21 unclear, 1/21 high Blinding of outcome assessment: 11/21 low, 7/21 unclear, 3/21 high Incomplete outcome data: 19/21 low, 2/21 high Selective reporting bias: 19/21 low, 2/21 unclear Other bias: 19/21 low, 2/21 unclear |  |  |  |  |  |  |
| Xie 2023 | Random sequence generation: 4/4 low Allocation concealment: 1/4 low, 3/4 unclear Blinding of participants/personnel: 3/4 low, 1/4 high Blinding of outcome assessment: 3/4 low, 1/4 high Incomplete outcome data: 4/4 low Selective reporting bias: 4/4 low Other bias: 4/4 low |  |  |  |  | Moderate certainty | Egger's/Begg's tests - reported neither revealed evidence of publication bias. |
| Xu 2023 | Random sequence generation: 84/90 low, 5/90 unclear, 1/90 high Allocation concealment: 11/90 low, 78/90 unclear, 1/90 high Blinding of participants/personnel: 36/90 low, 54/90 high Blinding of outcome assessment: 19/90 low, 71/90 unclear Incomplete outcome data: 82/90 low, 6/90 unclear, 2/90 high Selective reporting bias: 90/90 unclear Other bias: 90/90 unclear |  |  |  |  | Low certainty for global cognition outcome for a_tDCS, dual_tDCS, Disperse-dense wave electroacupuncture, Continuous wave electroacupuncture. Low certainty for p300 latency and amplitude outcomes for LF_rTMS, Continuous wave electroacupuncture, Disperse-dense wave electroacupuncture. Moderate certainty for global cognition outcome for HF_rTMS, Cerebellar fastigial nucleus stimulation, iTBS. Moderate certainty for p300 latency outcome for HF_rTMS and iTBS. Moderate certainty for p300 amplitude outcome for HF_rTMS. High certainty for global cognition outcome for LF_rTMS. | Funnel plots - "no apparent asymmetry". Egger's - "insignificant" publication bias. |
| Xu 2022 |  |  |  | Overall: 1/8 = 7 (high quality), 6/8 = 4, 1/8 = 3 (low quality) Random method: 7/8 = 2, 1/8 = 1 Blind method: 1/8 = 2, 7/8 = 1 Allocation concealment: 1/8 = 2, 7/8 = 1 Lost to follow-up: 1/8 = 1, 7/8 = 0 |  |  | Funnel plots - all asymmetrical indicating possible publication bias (due to limited quantities of studies and huge variations in score changes). |
| Yang 2024 (1) | Random sequence generation: 15/22 low, 7/22 unclear Allocation concealment: 5/22 low, 17/22 unclear Blinding of participants/personnel: 4/22 low, 18/22 unclear Blinding of outcome assessment: 5/22 low, 17/22 unclear Incomplete outcome data: 17/22 low, 5/22 unclear Selective reporting bias: 22/22 low Other bias: 22/22 low |  |  |  |  |  | Funnel plots: not symmetrical and some points fall outside suggesting small sample effect and publication bias. |
| Yang 2023 | Overall: 6/6 high Random sequence generation: 5/6 low, 1/6 unclear Allocation concealment: 4/6 low, 2/6 unclear Blinding of participants/personnel: 1/6 low, 5/6 high Blinding of outcome assessment: 5/6 low, 1/6 high Incomplete outcome data: 2/6 low, 3/6 unclear, 1/6 high Selective reporting bias: 5/6 low, 1/6 unclear Other bias: 3/6 low, 3/6 unclear |  |  |  |  |  | Egger's - no significant publication bias. Funnel chart included but not commented on by authors. |
| Yang 2024 (2) | Random sequence generation: 14/25 low, 8/25 unclear, 3/25 high Allocation concealment: 25/25 unclear Blinding of participants/personnel: 17/25 low, 8/25 unclear Blinding of outcome assessment: 8/25 low, 17/25 unclear Incomplete outcome data: 25/25 low Selective reporting bias: 25/25 low Other bias: 25/25 unclear |  |  |  |  |  |  |
| Yan 2024 | Random sequence generation: 8/12 low, 4/12 unclear Allocation concealment: 11/12 low, 1/12 unclear Blinding of participants/personnel: 9/12 low, 3/12 unclear Blinding of outcome assessment: 9/12 low, 3/12 unclear Incomplete outcome data: 9/12 low, 2/12 unclear, 1/12 high Selective reporting bias: 5/12 low, 7/12 unclear Other bias: 5/12 low, 7/12 unclear |  |  |  |  |  |  |
| Yan 2020 | Random sequence generation: 5/10 low, 5/10 unclear Allocation concealment: 1/10 low, 9/10 unclear Blinding of participants/personnel: 2/10 low, 8/10 high Blinding of outcome assessment: 2/10 low, 8/10 unclear Incomplete outcome data: 1/10 low, 9/10 unclear Selective reporting bias: 10/10 unclear Other bias: 10/10 unclear |  |  |  |  |  | Funnel plot/Egger's - unlikely/no significantly small study effects. |
| Yikun 2023 | Overall "quality": 1/15 low, 11/15 medium, 3/15 high Random sequence generation: 13/15 low, 2/15 high Allocation concealment: 3/15 low, 12/15 unclear Blinding of participants/personnel: 3/15 low, 12/15 unclear Blinding of outcome assessment: 3/15 low, 12/15 unclear Incomplete outcome data: 15/15 low Selective reporting bias: 15/15 low Other bias: 15/15 unclear |  |  |  |  | Very low certainty for P300 latency and amplitude outcomes. Low certainty for MMSE outcome. Moderate certainty for MoCA outcome. | Funnel plot slight asymmetry but Egger's indicated no significant publication bias. |
| Zhang 2021 (1) | Random sequence generation: 4/7 low, 3/7 unclear Allocation concealment: 3/7 low, 2/7 unclear, 2/7 high Blinding of participants/personnel: 2/7 unclear, 5/7 high Blinding of outcome assessment: 2/7 low, 5/7 high Incomplete outcome data: 4/7 low, 3/7 unclear Selective reporting bias: 5/7 low, 2/7 unclear Other bias: 5/7 low, 2/7 unclear |  | "The total score was divided into three level: (1) high quality (score 6–10), (2) fair quality (score 4–5) and (3) poor quality (score ≤ 3)." Only overall scores provided (5, 5, 5, 5, 5, 7, 8). Mean total = 5.71. |  |  |  |  |
| Zhang 2021 (2) | Random sequence generation: 7/10 low, 3/10 unclear Allocation concealment: 5/10 low, 5/10 unclear Blinding of participants/personnel: 10/10 low Blinding of outcome assessment: 5/10 low, 4/10 unclear, 1 high Incomplete outcome data: 10/10 low Selective reporting bias: 7/10 low, 3/10 unclear Other bias: 10/10 low Note all graded as low RoB for blinding of participants/personnel because the authors judged blinding to not be possible. |  |  |  |  |  | Funnel plots/Egger - no significant publication bias for global cognitive outcome. |
| Zhang 2023 | Random sequence generation: 5/11 low, 6/11 unclear Allocation concealment: 3/11 low, 8/11 unclear Blinding of participants/personnel: 2/11 low, 9/11 unclear Blinding of outcome assessment: 3/11 low, 8/11 unclear Incomplete outcome data: 10/11 low, 1/11 unclear Selective reporting bias: 10/11 low, 1/11 unclear Other bias: 11/11 low |  |  |  |  |  | Funnel plot - "generally symmetric" but later described inevitable publication bias. |
| Zhao 2024 |  |  |  |  |  |  | Funnel plot/Egger/Begg's demonstrated significant publication bias for tDCS effect on general cognitive function, but not for rTMS. |
| Zhou 2024 |  | Overall: 1/46 low, 45/46 some concerns Randomisation process: 1/46 low, 45/46 some concerns Deviations from the intended intervention: 44/46 low, 2/46 some concerns Missing outcome data: 45/46 low, 1/46 some concerns Measurement of the outcome: 46/46 low Selection of the reported result: 46/46 low |  |  |  |  | Funnel plots - demonstrated "basic symmetry" suggesting "low risk of publication bias" |
| Zhou 2020 | Random sequence generation: 37/37 low Allocation concealment: 8/37 low, 20/37 unclear, 9/37 high Blinding of participants/personnel: 1/37 low, 36/37 high Blinding of outcome assessment: 4/37 low, 30/37 unclear, 3/37 high Incomplete outcome data: 37/37 low Selective reporting bias: 37/37 low Other bias: 4/37 low, 32/37 unclear, 1/37 high |  |  |  |  | Moderate certainty for MMSE and MoCA outcomes. No explanation of downgrading decisions for MMSE outcome. | Funnel plots/Begg's - symmetrical with no significant publication bias. |
| Zhou 2022 | Random sequence generation: 9/10 low, 1/10 unclear Allocation concealment: 4/10 low, 6/10 unclear Blinding of participants/personnel: 6/10 low, 3/10 unclear, 1/10 high Blinding of outcome assessment: 4/10 low, 6/10 unclear Incomplete outcome data: 9/10 low, 1/10 unclear Selective reporting bias: 8/10 low, 2/10 high Other bias: 10/10 low |  |  |  |  |  | Funnel plot - probably publication bias. |
| Zhu 2024 | Overall: Described as all "mild risk of bias" but 4 were high risk in 1 category Random sequence generation: 8/10 low, 2/10 unclear Allocation concealment: 5/10 low, 5/10 unclear Blinding of participants/personnel: 9/10 low, 1/10 high Blinding of outcome assessment: 5/10 low, 5/10 unclear Incomplete outcome data: 7/10 low, 3/10 high Selective reporting bias: 10/10 low Other bias: 10/10 low  Descriptions do not align with RoB table. |  |  |  |  |  | Funnel plot/Begg - asymmetrical. Egger's - does not support existence of publication bias. |
| Zhuo 2023 | Random sequence generation: 13/14 low, 1/14 unclear Allocation concealment: 2/14 low, 12/14 unclear Blinding of participants/personnel: 5/14 low, 9/14 unclear Blinding of outcome assessment: 5/14 low, 9/14 unclear Incomplete outcome data: 14/14 low Selective reporting bias: 12/14 low, 2/14 unclear Other bias: 6/14 low, 8/14 unclear Descriptions do not align with RoB table. |  |  |  |  |  |  |

*Where possible risk of bias findings have been extracted for RCTs included in SRs’ global cognition outcome only.

**Supplementary Figure 2. Summary of risk of bias of RCTs as reported by systematic reviews using the Cochrane Risk of Bias Tool for Randomized Trials (RoB 2).**

**Supplementary Figure 3. Summary of RCT quality as reported by systematic reviews using the Physiotherapy Evidence Database (PEDro) Scale.**

* Not reported by all SRs.

**Supplementary Table 7. Summary of GRADE analyses for individual outcomes and interventions as reported by Systematic Reviews.**

| **SR Ref** | **Outcome / Intervention** | | | **GRADE assessment** | | | | | | |
| --- | --- | --- | --- | --- | --- | --- | --- | --- | --- | --- |
|  | **Global cognition tool(s)/outcome** | **Intervention/ comparator subgroup** | **Intervention category** | **Certainty** | **Risk of bias** | **Inconsistency of results** | **Indirectness of evidence** | **Imprecision of results** | **Publication bias** | **Other considerations** |
| Chen 2022 (1) | Global cognition (MMSE) | All | Non-invasive brain stimulation | Moderate | NR | NR | NR | Very serious imprecision due to the small sample size (< 400 individuals) and wide confidence interval | NR |  |
| Daoud 2024 | Global cognition (MMSE, RBANS, LOTCA) | All | Non-invasive brain stimulation | Low | One study had a low ROB, four studies had an overall unclear ROB, and one study (Chu [21]) had a high risk of detection bias. However, it was downgraded for other reasons. | Downgraded by one level for inconsistency (I2 = 79%). | NR | Downgraded by one level for imprecision. The total sample size population was smaller than The optimal information size(OIS) (< 800). | NR |  |
| Elsner 2020 | Global cognition (Computerized Neuropsychological Test, K-MMSE, SCNT) | tDCS vs placebo or passive control | Non-invasive brain stimulation | Low | NR | NR | NR | Downgraded one level because the total sample size is less than 400 (as a rule of thumb for implementing GRADE 'optimal information size' criteria). | Publication bias strongly suspected by visual inspection of funnel plot. Downgraded one level. |  |
| Fan 2022 | MoCA | NBP monotherapy | DL-3-n-Butylphthalide (NBP) | Very low | Serious (most studies are at high RoB) | Serious (50% < I2 < 75%) | Not serious | Not serious | Strongly suspected (too few studies) |  |
|  |  | NBP combination therapy | DL-3-n-Butylphthalide (NBP) + Oxiracetam, DL-3-n-Butylphthalide (NBP) + Nimodipine, DL-3-n-Butylphthalide (NBP) + Donepezil, DL-3-n-Butylphthalide (NBP) + Citicoline | Low | Not serious | Serious (50% < I2 < 75%) | Not serious | Not serious | Strongly suspected (apparent asymmetry in funnel plot). |  |
|  | MMSE | NBP monotherapy | DL-3-n-Butylphthalide (NBP) | Low | Serious (most studies are at high RoB) | Not serious | Not serious | Not serious | Strongly suspected (too few studies) |  |
|  |  | NBP combination therapy | DL-3-n-Butylphthalide (NBP) + Oxiracetam, DL-3-n-Butylphthalide (NBP) + Nimodipine, DL-3-n-Butylphthalide (NBP) + Donepezil, DL-3-n-Butylphthalide (NBP) + Citicoline | Very low | Serious (most studies are at high RoB) | Serious (50% < I2 < 75%) | Not serious | Not serious | Strongly suspected (apparent asymmetry in funnel plot). |  |
| Gao 2023 | Global cognition (MMSE, MoCA) | All | Non-invasive brain stimulation + Cognitive rehabilitation | Low | Very serious. Downgraded one level due to risk of bias: the overall risk of bias for all the included trials assessed by ROB 2 showed high risk or some concerns due to the bias in the randomization process, deviations from intended interventions, missing outcome data, selection of the reported result or no blinding of participants or investigators. | Not serious | Not serious | Not serious. Downgraded one level due to imprecision: total sample size < 400 (adequate sample size). | NR |  |
| Gibson 2022 | Global cognition (MoCA, MMSE, BNIS) | All | Other rehabilitation therapies | Low | Downgraded two levels due to very serious concerns about risk of bias: all were unclear for allocation concealment except Jiang 2016; Jiang 2016, the highest weighted study, had potential for high risk for incomplete outcome data. | NR | NR | NR | NR |  |
| Gong 2023 | MMSE | All | Non-invasive brain stimulation | Moderate | Not serious | Not serious | Not serious | Downgraded (n < 200) | Not serious | Effect size = Not serious. |
|  | MoCA | All | Non-invasive brain stimulation | Moderate | Not serious | Not serious | Not serious | Downgraded (n < 200) | Not serious | Effect size = Not serious. |
|  | LOTCA | All | Non-invasive brain stimulation | Moderate | Not serious | Not serious | Not serious | Downgraded (n < 200) | Not serious | Effect size = Not serious. |
| Han 2023 | MMSE | All | Non-invasive brain stimulation | Moderate | Most of the RCTs were low quality with an inadequate level of blinding and unclear risk of concealment of allocation. | NR | NR | NR | NR |  |
|  | MoCA | All | Non-invasive brain stimulation | Moderate | NR | The statistical test for heterogeneity showed that large variation (I2 > 50%) existed in point estimates due to the among study differences. | NR | NR | NR |  |
| Khattab 2021 | MoCA, FIM (cognitive domain), ACE-revised | All | Exercise | Low | Not serious | Not serious | Not serious | Very serious (values were displayed with small sample sizes (n < 300)). | None |  |
| Kuang 2021 | MoCA | All | Acupuncture | Very low | Very serious (insufficient allocation concealment, and blinding of participants and personnel are not adopted or described, insufficient blinding of outcome assessment, unclear selective reporting). | Serious (p = 0.0007, I2 = 63%). | Not serious | Not serious | NR |  |
|  | MMSE | All | Acupuncture | Very low | Very serious (insufficient allocation concealment, and blinding of participants and personnel are not adopted or described, insufficient blinding of outcome assessment, unclear selective reporting). | Serious (p < 0.00001, I2 = 92%). | Not serious | Not serious | NR |  |
| Li 2021 | MMSE | Acupuncture + TMS vs Conventional treatment (Direct) | Acupuncture + Non-invasive brain stimulation | Very low | Downgraded | NR | NR | Downgraded | Downgraded |  |
|  |  | Acupuncture + HBO vs Conventional treatment (Direct) | Acupuncture + Hyperbaric oxygen therapy | Very low | Downgraded | NR | NR | Downgraded | Downgraded |  |
|  |  | Acupuncture + UWE vs Conventional treatment (Indirect) | Acupuncture + Electrotherapy | Low | NR | NR | Downgraded | Downgraded | NR |  |
|  |  | Acupuncture + RehaCom vs Conventional treatment (Direct) | Acupuncture + Cognitive rehabilitation | Low | NR | NR | NR | Downgraded | Downgraded |  |
|  |  | Acupuncture + TMS vs Acupuncture (Direct) | Acupuncture + Non-invasive brain stimulation | Moderate | NR | NR | NR | NR | Downgraded |  |
|  |  | Acupuncture + HBO vs Acupuncture (Direct) | Acupuncture + Hyperbaric oxygen therapy | Very low | Downgraded | NR | NR | Downgraded | Downgraded |  |
|  |  | Acupuncture + UWE vs Acupuncture (Direct) | Acupuncture + Electrotherapy | Moderate | NR | NR | NR | NR | Downgraded |  |
|  |  | Acupuncture + RehaCom vs Acupuncture (Direct) | Acupuncture + Cognitive rehabilitation | Low | NR | Downgraded | NR | NR | Downgraded |  |
|  |  | Acupuncture + TMS vs HBO (Indirect) | Acupuncture + Non-invasive brain stimulation | Very low | Downgraded | NR | Downgraded | NR | Downgraded |  |
|  |  | Acupuncture + HBO vs HBO (Direct) | Acupuncture + Hyperbaric oxygen therapy | Moderate | NR | NR | NR | Downgraded | NR |  |
|  |  | Acupuncture + UWE vs HBO (Indirect) | Acupuncture + Electrotherapy | Very low | Downgraded | NR | Downgraded | NR | Downgraded |  |
|  |  | Acupuncture + RehaCom vs HBO (Indirect) | Acupuncture + Cognitive rehabilitation | Very low | Downgraded | NR | Downgraded | NR | Downgraded |  |
| Liu 2021 | MMSE | All | Non-invasive brain stimulation | Moderate | NR | NR | NR | The inaccuracy of the results was reflected in the inclusion of less than 100 samples for each outcome | NR |  |
|  | LOTCA | All | Non-invasive brain stimulation | Moderate | NR | NR | NR | The inaccuracy of the results was reflected in the inclusion of less than 100 samples for each outcome | NR |  |
| Liu 2023 (2) | MMSE | All | Acupuncture | Low | Serious | Serious | Not serious | Not serious | NR |  |
|  | MoCA | All | Acupuncture | Low | Serious | Serious | Not serious | Not serious | NR |  |
|  | LOTCA | All | Acupuncture | Low | Serious | Serious | Not serious | Not serious | NR |  |
| Liu 2023 (3) | MMSE | SA + BA vs Cognitive training | Acupuncture | Low | Serious | Serious | Not serious | Not serious | Not serious |  |
|  |  | Scalp acupuncture + Cognitive training vs Cognitive training | Acupuncture + Cognitive rehabilitation | Low | Serious | Serious | Not serious | Not serious | Not serious |  |
|  |  | Body acupuncture + Cognitive training vs Cognitive training | Acupuncture + Cognitive rehabilitation | Low | Serious | Not serious | Not serious | Serious | Not serious |  |
|  |  | Ophthalmic acupuncture + Cognitive training vs Cognitive training | Acupuncture + Cognitive rehabilitation | Low | Serious | Not serious | Not serious | Serious | Not serious |  |
|  |  | Warm acupuncture + Cognitive training vs Cognitive training | Acupuncture + Cognitive rehabilitation | Low | Serious | Not serious | Not serious | Serious | Not serious |  |
|  |  | Auricular bloodletting + Cognitive training vs Cognitive training | Acupuncture + Cognitive rehabilitation | Low | Serious | Not serious | Not serious | Serious | Not serious |  |
|  |  | Scalp acupuncture + Auricular bloodletting vs Cognitive training | Acupuncture | Low | Serious | Not serious | Not serious | Serious | Not serious |  |
|  |  | Scalp acupuncture vs Cognitive training | Acupuncture | Low | Serious | Not serious | Not serious | Serious | Not serious |  |
|  |  | Body acupuncture vs Cognitive training | Acupuncture | Very low | Serious | Not serious | Not serious | Very serious | Not serious |  |
|  |  | Electro-acupuncture + Cognitive training vs Cognitive training | Acupuncture + Cognitive rehabilitation | Very low | Serious | Serious | Not serious | Very serious | Not serious |  |
|  |  | Abdominal acupuncture + Body acupuncture vs Cognitive training | Acupuncture | Very low | Serious | Not serious | Not serious | Very serious | Not serious |  |
|  |  | Abdominal acupuncture + Cognitive training vs Cognitive training | Acupuncture + Cognitive rehabilitation | NR | NR | NR | NR | NR | NR |  |
|  | MoCA | Scalp acupuncture + Cognitive training vs Cognitive training | Acupuncture + Cognitive rehabilitation | Low | Serious | Serious | Not serious | Not serious | Not serious |  |
|  |  | Scalp acupuncture + Body acupuncture vs Cognitive training | Acupuncture | Moderate | Serious | Not serious | Not serious | Not serious | Not serious |  |
|  |  | Warm acupuncture + Cognitive training vs Cognitive training | Acupuncture + Cognitive rehabilitation | Low | Serious | Serious | Not serious | Not serious | Not serious |  |
|  |  | Electro-acupuncture + Cognitive training vs Cognitive training | Acupuncture + Cognitive rehabilitation | Low | Serious | Not serious | Not serious | Serious | Not serious |  |
|  |  | Abdominal acupuncture + Cognitive training vs Cognitive training | Acupuncture + Cognitive rehabilitation | Low | Serious | Not serious | Not serious | Serious | Not serious |  |
|  |  | Scalp acupuncture vs Cognitive training | Acupuncture | Very low | Serious | Not serious | Not serious | Very serious | Not serious |  |
|  |  | Warm acupuncture + Scalp acupuncture vs Cognitive training | Acupuncture | Very low | Serious | Not serious | Not serious | Very serious | Not serious |  |
|  |  | Body acupuncture + Cognitive training vs Cognitive training | Acupuncture + Cognitive rehabilitation | NR | NR | NR | NR | NR | NR |  |
|  |  | Body acupuncture vs Cognitive training | Acupuncture | NR | NR | NR | NR | NR | NR |  |
| Li 2022 (3) | MoCA | TMS vs Normal rehabilitation | Non-invasive brain stimulation | Low | Serious | Serious | Not serious | Not serious | Not serious |  |
|  |  | tDCS vs Normal rehabilitation | Non-invasive brain stimulation | Low | Serious | Serious | Not serious | Not serious | Not serious |  |
|  |  | VR vs Normal rehabilitation | Virtual reality | Low | Serious | Not serious | Not serious | Serious | Not serious |  |
|  |  | CACR vs Normal rehabilitation | Cognitive rehabilitation | Low | Serious | Serious | Not serious | Not serious | Not serious |  |
|  |  | Acupuncture vs Normal rehabilitation | Acupuncture | Low | Serious | Serious | Not serious | Not serious | Not serious |  |
|  | MMSE | TMS vs Normal rehabilitation | Non-invasive brain stimulation | Low | Serious | Serious | Not serious | Not serious | Not serious |  |
|  |  | tDCS vs Normal rehabilitation | Non-invasive brain stimulation | Low | Serious | Not serious | Not serious | Serious | Not serious |  |
|  |  | VR vs Normal rehabilitation | Virtual reality | Very low | Very serious | Not serious | Not serious | Serious | Not serious |  |
|  |  | CACR vs Normal rehabilitation | Cognitive rehabilitation | Low | Serious | Not serious | Not serious | Serious | Not serious |  |
|  |  | Acupuncture vs Normal rehabilitation | Acupuncture | Low | Serious | Serious | Not serious | Not serious | Not serious |  |
| Lin 2024 | Global cognition (MoCA, MMSE, FIM, ACE, Timed up and go cognition test) | All | Virtual reality | Low | Serious (High risk of performance bias due to the absence of blinding to participants, personnel, and of detection bias due to the absence of outcome assessment) | Serious (substantial heterogeneity was detected) | Not serious | Serious (most studies involved small effect size <30) | NR | Strong association |
| Shen 2022 | MoCA | All | Traditional Chinese medicine | Very low | Serious (most studies are at high RoB) | Very serious (I2 ≥ 75%) | Not serious | Not serious | Strongly suspected (based on the publication bias test, there is apparent asymmetry in the funnel plot) |  |
|  | MMSE | All | Traditional Chinese medicine | Very low | Serious (most studies are at high RoB) | Very serious (I2 ≥ 75%) | Not serious | Not serious | Strongly suspected (based on the publication bias test, there is apparent asymmetry in the funnel plot) |  |
| Toh 2023 | Global cognition (MMSE, MoCA, cognitive subscale of Canadian Neurological Scale) | All | Non-invasive ventilation | Very low | Very serious (may be influenced by lack of personnel and outcome assessor blinding, high risk for attrition bias) | Very serious (considerable heterogeneity (I² = ≥ 75%)) | Serious (Patients all had ischemic/hemorrhagic stroke and were only treated with relevant non-invasive PAP devices. Treatment initiated at different post-stroke phases, and the compliance rate towards PAP treatment can vary. Subgroup analyses could not be performed to stratify the duration of PAP use and stroke phase. Outcomes are measured using relevant scales. However, more studies are required) | Not serious (95% CI excludes line of null effect, and OIS criterion was met. The sample size (n = 454) exceeded the calculated minimum OIS of 46) | NR | "-All plausible residual confounding would reduce the demonstrated effect -dose response gradient" |
| Wang 2023 | Global cognition (MMSE, MoCA) | All | Non-invasive brain stimulation | Moderate | Not serious | Serious (I-squared >50%) | Not serious | Not serious | NR | "None" |
| Xiao 2024 | Global cognition (MMSE, MoCA) | All | Acupuncture + Non-invasive brain stimulation | Low | Serious (the risk of bias is reduced by one level: Some studies have unclear reports on randomized methods, allocation concealment, and blind methods) | Serious (reduce the risk of inconsistency by one level: there is inconsistency in the results without a reasonable explanation) | Not serious | Not serious | Not found |  |
| Xie 2023 | Global cognition (MoCA) | All | Non-invasive brain stimulation | Moderate | Allocation concealment is unclear | NR | NR | NR | NR |  |
| Xu 2023 | Global cognition (MMSE, MoCA, LOTCA, HDS) | LF_rTMS | Non-invasive brain stimulation | High | NR | NR | NR | NR | NR |  |
|  |  | HF_rTMS | Non-invasive brain stimulation | Moderate | Downgraded | NR | NR | NR | NR |  |
|  |  | a_tDCS | Non-invasive brain stimulation | Low | Downgraded | NR | NR | Downgraded | NR |  |
|  |  | dual_tDCS | Non-invasive brain stimulation | Low | Downgraded | NR | NR | Downgraded | NR |  |
|  |  | Disperse-dense wave electroacupuncture | Acupuncture | Low | Downgraded | NR | NR | Downgraded | NR |  |
|  |  | Continuous wave electroacupuncture | Acupuncture | Low | Downgraded | NR | NR | Downgraded | NR |  |
|  |  | Cerebellar fastigial nucleus stimulation | Non-invasive brain stimulation | Moderate | NR | NR | NR | Downgraded | NR |  |
|  |  | iTBS | Non-invasive brain stimulation | Moderate | NR | NR | NR | Downgraded | NR |  |
|  | P300 latency | LF_rTMS | Non-invasive brain stimulation | Low | Downgraded | NR | NR | Downgraded | NR |  |
|  |  | HF_rTMS | Non-invasive brain stimulation | Moderate | Downgraded | NR | NR | NR | NR |  |
|  |  | Continuous wave electroacupuncture | Acupuncture | Low | Downgraded | NR | NR | Downgraded | NR |  |
|  |  | Disperse-dense wave electroacupuncture | Acupuncture | Low | Downgraded | NR | NR | Downgraded | NR |  |
|  |  | iTBS | Non-invasive brain stimulation | Moderate | NR | NR | NR | Downgraded | NR |  |
|  | P300 amplitude | LF_rTMS | Non-invasive brain stimulation | Low | Downgraded | NR | NR | Downgraded | NR |  |
|  |  | HF_rTMS | Non-invasive brain stimulation | Moderate | Downgraded | NR | NR | NR | NR |  |
|  |  | Continuous wave electroacupuncture | Acupuncture | Low | Downgraded | NR | NR | Downgraded | NR |  |
|  |  | Disperse-dense wave electroacupuncture | Acupuncture | Low | Downgraded | NR | NR | Downgraded | NR |  |
| Yikun 2023 | MoCA | All | Non-invasive brain stimulation | Moderate | - | - | - | Downgraded (small sample size, wide confidence intervals) | - |  |
|  | MMSE | All | Non-invasive brain stimulation | Low | - | Downgraded (small p-value, large I2 value) | - | Downgraded (small sample size, wide confidence intervals) | - |  |
|  | P300 latency | All | Non-invasive brain stimulation | Very low | Downgraded (included studies have significant RoB in randomisation, allocation concealment and blinding) | Downgraded (small p-value, large I2 value) | - | Downgraded (small sample size, wide confidence intervals) | - |  |
|  | P300 amplitude | All | Non-invasive brain stimulation | Very low | Downgraded (included studies have significant RoB in randomisation, allocation concealment and blinding) | Downgraded (small p-value, large I2 value) | - | Downgraded (small sample size, wide confidence intervals) | - |  |
| Zhou 2020 | MMSE | All | Acupuncture | Moderate | NR | NR | NR | NR | NR |  |
|  | MoCA | All | Acupuncture | Moderate | Downgraded (many of the RCTs were low quality with an inadequate level of blinding because blindings in the acupuncture therapy were difficult for the therapists and patients) | NR | NR | NR | NR |  |
